# Determination of piperaquine concentration in human plasma and the correlation of capillary versus venous plasma concentrations

**DOI:** 10.1371/journal.pone.0233893

**Published:** 2020-05-29

**Authors:** Norah Mwebaza, Vincent Cheah, Camilla Forsman, Richard Kajubi, Florence Marzan, Erika Wallender, Grant Dorsey, Philip J. Rosenthal, Francesca Aweeka, Liusheng Huang

**Affiliations:** 1 Infectious Disease Research Collaboration, Makerere University College of Health Sciences, Kampala, Uganda; 2 Drug Research Unit, Department of Clinical Pharmacy, University of California San Francisco, San Francisco, CA, United States of America; 3 Department of Medicine, University of California San Francisco, San Francisco, CA, United States of America; Academic Medical Centre, NETHERLANDS

## Abstract

**Background:**

A considerable challenge in quantification of the antimalarial piperaquine in plasma is carryover of analyte signal between assays. Current intensive pharmacokinetic studies often rely on the merging of venous and capillary sampling. Drug levels in capillary plasma may be different from those in venous plasma, Thus, correlation between capillary and venous drug levels needs to be established.

**Methods:**

Liquid chromatography coupled with tandem mass spectrometry (LC-MS/MS) was used to develop the method. Piperaquine was measured in 205 pairs of capillary and venous plasma samples collected simultaneously at ≥24hr post dose in children, pregnant women and non-pregnant women receiving dihydroartemisinin-piperaquine as malaria chemoprevention. Standard three-dose regimen over three days applied to all participants with three 40mg dihydroartemisinin/320mg PQ tablets per dose for adults and weight-based dose for children. Correlation analysis was performed using the program Stata® SE12.1. Linear regression models were built using concentrations or logarithm transformed concentrations and the final models were selected based on maximal coefficient of determination (R^2^) and visual check.

**Results:**

An LC-MS/MS method was developed and validated, utilizing methanol as a protein precipitation agent, a Gemini C_18_ column (50x2.0mm, 5μm) eluted with basic mobile phase solvents (ammonium hydroxide as the additive), and ESI^+^ as the ion source. This method had a calibration range of 10–1000 ng/mL and carryover was negligible. Correlation analysis revealed a linear relationship: C_cap_ = 1.04×C_ven_+4.20 (R^2^ = 0.832) without transformation of data, and lnC_cap_ = 1.01×lnC_ven_+0.0125, (R^2^ = 0.945) with natural logarithm transformation. The mean ratio (±SD) of C_cap_/C_ven_ was 1.13±0.42, and median (IQR) was 1.08 (0.917, 1.33).

**Conclusions:**

Capillary and venous plasma PQ measures are nearly identical overall, but not readily exchangeable due to large variation. Further correlation study accounting for disposition phases may be necessary.

## Introduction

**Fig 1:**
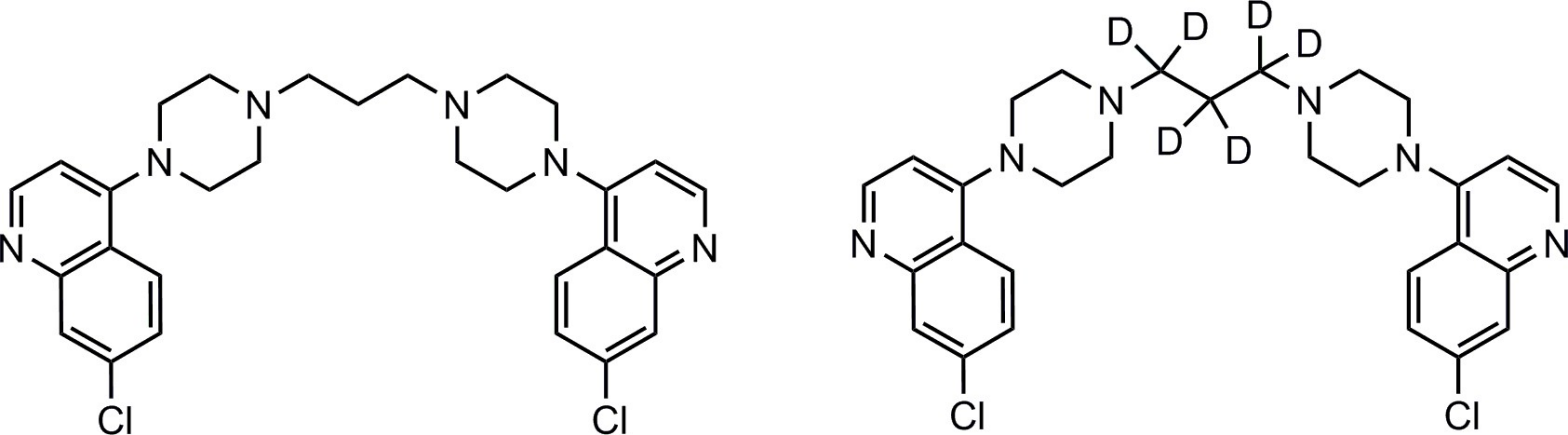
Chemical structures of piperaquine (left) and piperaquine-d6 (right).

Piperaquine (PQ) is a long-acting antimalarial drug currently used in combination with dihydroartemisinin for malaria treatment, and also studied for chemoprevention [1, 2]. PQ (Fig 1) is a weak base with pKa values around 8.6 and 6.5 and high lipophilicity (LogP = 6.2) at neutral and alkaline pH [3]. The free base form of PQ is poorly soluble in water, methanol (MeOH), and acetonitrile (MeCN), but very hydrophilic at low pH, and easily soluble in acidified solvents.

Adsorption of PQ to a glass surface occurs if the solution is stored in a glass container, and loss of PQ signal is observed when a metal injection needle or metal tubing is used in an analytical system. Our group found over 50% PQ loss after storage in a glass tube overnight (S1 Table). The extent of adsorption is in the following order: silanized glass > glass > polypropylene plastic tube > Thermo Scientific™ low retention tubes. In plasma samples, over 97% of PQ binds to proteins [4, 5]. These properties present daunting challenges for method development. A considerable challenge is carryover between assays. Carryover could be due to the column (tailing peak), precolumn tubing, or autosampler. Numerous methods have been published for the quantification of PQ in plasma, including high performance liquid chromatography-ultraviolet/visible spectrophotometry (HPLC-UV) [5–7] and liquid chromatography tandem mass spectrometry (LC-MS/MS) [8–12]. Carryover was observed in nearly all LC-MS/MS methods. Here we report an LC-MS/MS method with negligible carryover and its application to venous and capillary samples simultaneously collected for correlation analysis.

Venous blood sampling via catheter remains to be the mainstream method for clinical sample collection. Capillary blood sample collection via finger or heel stick could be used as an alternative method, especially in rural areas and pediatric patients [2]. Current intensive pharmacokinetic designs are often relying on the merging of venous and capillary sampling [13, 14]. However, drug levels in capillary plasma may be different from those in venous plasma because of difference in matrix contents, such as oxygen and protein [15, 16]. Correlation between capillary and venous drug levels needs to be established in order to properly interpret and analyze results. A previous study has suggested capillary blood PQ concentrations are higher on average than venous blood PQ in malaria patients [17]. Here we report correlation of venous plasma and capillary plasma PQ concentrations in participants receiving dihydroartemisinin-PQ when enrolled in a malaria chemoprevention trial.

## Methods

### Materials

Piperaquine tetraphosphate tetrahydrate (MW 999.55, purity 99%) was purchased from A.K.Scientific Inc. (Union City, CA, USA). Piperaquine-d_6_ (PQ-d_6_, MW 541.55, isotopic purity ≥99%) was purchased from AlSAchim, SAS (IllKirch, France). Trichloroacetic acid (TCA, certified ACS reagent) and ammonium formate (NH_4_FA,certified ACS reagent), trifluoroacetic acid (TFA, Optima™ LC/MS grade), formic acid (FA, Optima™ LC/MS grade) and ammonium hydroxide (NH_4_OH) (Optima™ LC/MS grade), acetonitrile (MeCN, HPLC grade), methanol (MeOH, HPLC grade), and other common solvents (HPLC grade) were purchased from Fisher Scientific Co. (Fair Lawn, NJ, USA). Blank human plasma (K_3_EDTA added as anticoagulant) was obtained from Biological Specialty Co (Colmar, PA, USA).

### Quantitation method

Sciex API2000 tandem mass spectrometer was coupled with a Perkin Elmer 200 series micro LC system. The LC column was Gemini C_18_ (50×2.0 mm, 5μm) fitted with a guard column (4×2.0 mm, 5μm) (Phenomenex Inc., Torrance, CA, USA), eluted with 10 mM NH_4_OH (A) and MeCN (B) in a gradient mode at a flow rate of 0.6 mL/min. ElectroSpray ionization in positive mode (ESI^+^) was used as the ion source with multiple reaction monitoring (MRM) of *m/z* 535/288 for PQ and *m/z* 541/294 for the IS (PQ-d_6_) for quantitation. PQ stock solution was prepared in MeCN-water (1:9, v/v) containing 0.5% formic acid. Calibration standard samples (10, 25, 50, 100, 250, 500, and 1000 ng/mL) and QC samples (30, 200, and 800 ng/mL) were prepared in blank plasma from two different stock solutions. Plasma samples (25 μL) were mixed with 25 μL 30 ng/mL PQ-d_6_ in MeCN-water (1:9, v/v) containing 0.5%FA, added 150 μL MeOH, briefly vortex-mixed, and centrifuged at 25,000g for 5min. Transferred ~100μL supernatant to plastic sample vials or 96-well plate in autosampler. Injection volume was 10 μL.

### Validation

The method was validated based on the NIH-sponsored CPQA guidelines [18], which was adopted from the FDA guidelines [19]. A full validation includes precision and accuracy, matrix effect and recovery, dilution integrity and partial volume, stability, concomitant drug interference, and cross validation with a published method. Dilution integrity was evaluated by diluting the extra-high QC sample (3000 ng/mL) by 3-, 5-, and 10-fold with blank plasma. Partial volume was evaluated by mixing 12.5 μL QC samples with 12.5 μL blank plasma. Stability in plasma was evaluated at 4°C overnight (16hr) in glass vial and after 5 freeze-thaw cycles by comparing the treated samples with untreated samples in plastic microcentrifuge tubes. Long term stability in plasma at -70°C for 21 months was tested with the published method as the concentrations of old QC samples were made for the method [3]. To evaluate autosampler stability, the processed low and high QC samples were tested on the same day of processing (as control) and after 7 days in autosampler plastic vials. Stock solution stability was evaluated on UPLC-PDA system by diluting the stock to 10 ug/mL in MeCN-water (1:9) containing 0.5%FA. All measurements were performed in at least triplicates. Matrix effect was evaluated with 7 different lots of human plasma with K_3_EDTA as the anticoagulant. Set 1 samples were prepared by spiking both PQ and IS in 75% MeOH with final concentrations of 3.75, 25, and 100 ng/mL for PQ and 3.75 ng/mL for IS, corresponding to the final concentrations of PQ and IS from plasma samples after protein precipitation. Set 2 samples were spiked at the same concentration as Set 1 in extracted solutions from 7 lots of blank plasma, and Set 3 samples were prepared by spiking PQ in 7 lots of blank plasma with a final PQ concentration of 30, 200, and 800 ng/mL and then processing the plasma samples as described above.

### Clinical sample analysis

The method applied to capillary versus venous plasma PQ correlation study, which is part of a pharmacokinetic study within the larger trials for malaria chemoprevention in pregnant women and children (ClinicalTrials.gov number, NCT02163447) [13, 14, 20, 21]. The study was conducted in Tororo, Uganda from December 2014 to May 2017. Eligible participants were pregnant women with ultrasound-estimated gestational age of 12–20 weeks and their children. Complete entry criteria were summarized previously [13, 14, 22]. The studies were approved by the Uganda National Council of Science and Technology and institutional review boards of Makerere University and the University of California, San Francisco. Written informed consent was obtained from adult study participants, and, for children, from their parents or guardians. The reported method was used to analyze 150 pairs of plasma samples from capillary and venous blood simultaneously collected 24 hr post last dose. We also modified a previously published method in our group to a lower calibration range of 0.5–50 ng/mL [3], and performed the required partial validation for this modification (S1 File). The modified method was used for 65 pairs of plasma samples from capillary and venous blood simultaneously collected from 7 to 84 days post 1^st^ dose. Standard three-dose regimen over three days applied to all participants with three 40mg dihydroartemisinin/320mg PQ tablets per dose for adults [13] and weight-based dose for children [14].

### Correlation analysis

Using STATA SE12.1, the relationship between capillary and venous plasma PQ concentrations was modelled using a linear relationship with estimated intercept and slope. The linear least squares regression models were built using concentrations or logarithm transformed concentrations and the final models were selected based on maximal coefficient of determination (R^2^) and visual check.

## Results

### 1. Method development

**Fig 2:**
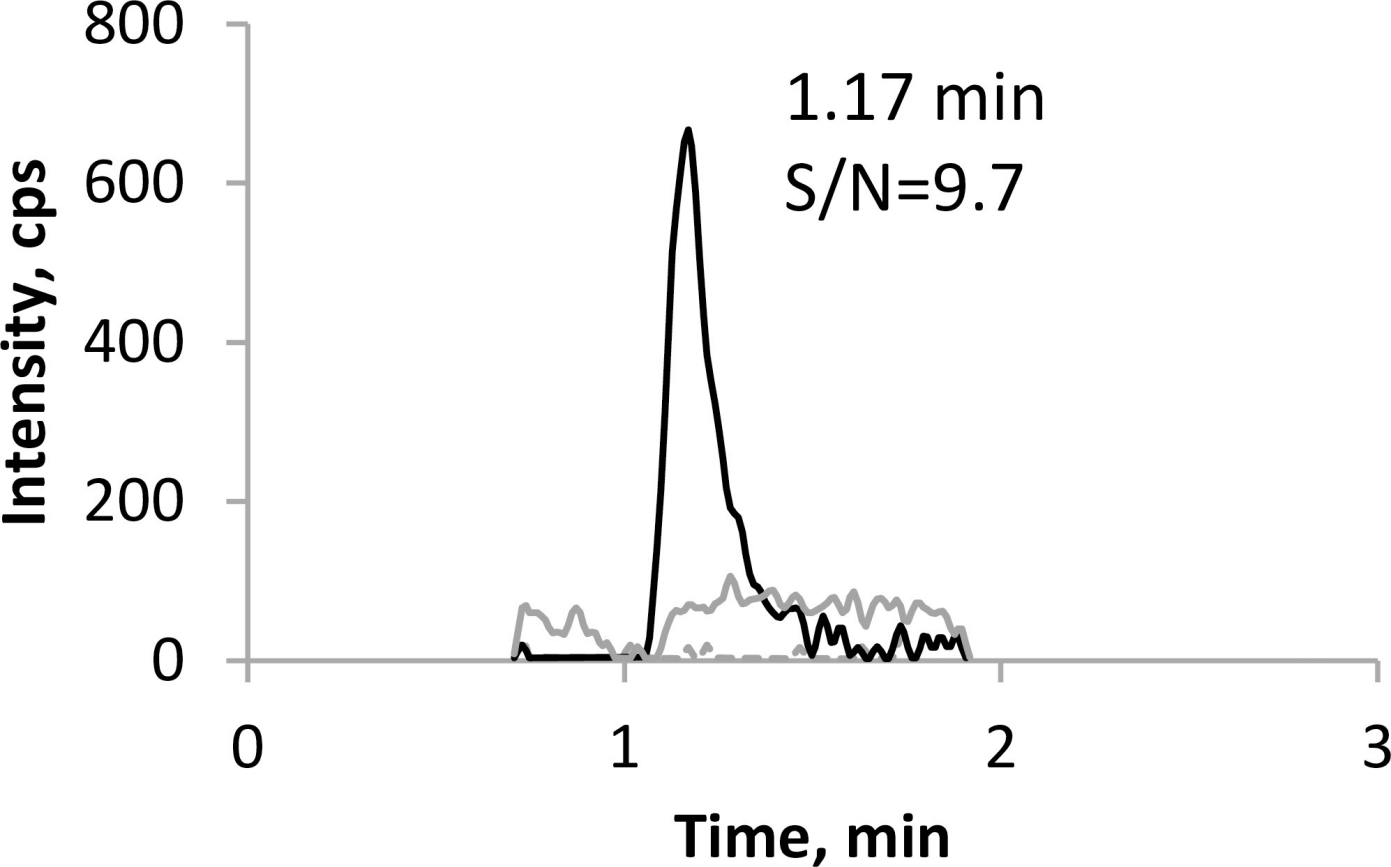
Chromatograms of PQ representing blank plasma (dashed gray line), the LLOQ (solid black line) and blank plasma after ULOQ, indicating carryover (solid gray line).

The optimized MS parameters were shown in Table 1. Most published methods for analysis of PQ have utilized acidic mobile phase solvents. Alternatively, basic mobile phase solvents can be used. Lindegardh’s group published a method using 2.5 mM ammonium bicarbonate (pH10.0)-acetonitrile (15:85, v/v) as the mobile phase solvents [8]. Based on this method, we developed a new method on an API2000 system. A tailing peak was observed when we tried to use the same solvents [23], so we decided to use ammonium hydroxide in the mobile phase. Higher NH_4_OH concentration led to better peak shape, but lower signal intensity. Optimal peaks were observed using 10 mM NH_4_OH as mobile phase A, and 100% acetonitrile as mobile phase B (Fig 2). The final LC gradient program consists of 55% solvent B (0 min), from 55 to 100% B (0–1.5 min), 100% B (1.5–2.0 min), 100%-55% B (2.0–2.1 min), and 55% B (2.1–2.5 min). Surprisingly, no carryover peaks were observed with this method, though occasionally a small carryover peak was found in some runs, probably due to pH variation of mobile phase A. Ammonia may evaporate and carbon dioxide in air may dissolve in mobile phase A to change the pH over time. Therefore, we recommend that mobile phase A be prepared freshly every day before use. Disappearance of carryover peak may also possibly because the API2000 system is less sensitive.

**Table 1:** Optimized MS parameters.

Source parameters	TEM,°C	IS, v	CAD, psi	CUR, psi	Gas1, psi	Gas2, psi	
	500	2000	11	30	40	70	
Compound parameters	DP, v	FP, v	EP, v	CE, v	CEP, v	CXP, v	Dwell time, ms
535/288 (PQ)	76	360	12	49	20	10	250
541/294 (PQ-d_6_, I.S.)	76	360	12	49	20	10	250

TEM, source temperature; IS, ionspray voltage; CUR, curtain gas, Gas1, nebulizer gas; Gas2, auxiliary gas; CAD, collision-activated dissociation; DP, declustering potential; FP, focusing potential; EP, entrance potential; CE, collision energy; CEP, collision cell entrance potential; CXP, collision cell exit potential.

### 2. Method validation

The lower limit of quantitation (LLOQ) for this assay was set at 10 ng/mL, with a S/N ratio = 9.7. The calibration range was 10–1000 ng/mL. The calibration curve was fitted with least square linear regression weighted by 1/x. The correlation coefficient (r) was typically > 0.9990. Representative chromatograms of PQ from blank plasma extract, LLOQ, and blank plasma extract injected after the upper limit of quantitation (ULOQ) are shown in Fig 2.

The intra-day precision (n = 6) over 3 days ranged from 1.2 to 4.0% at the three concentrations (30, 200, and 800 ng/mL), and inter-day precisions ranged from 2.7 to 4.6%, all of them within 15% deviation from mean values. The intra- and inter-day accuracy ranged from 0.4 to 9.1%and 5.1 to 5.3%, respectively, all within 15% deviation from the nominal values. At the LLOQ 10 ng/mL level, the precision and accuracy met the criteria of <20% (Table 2).

**Table 2:** Intra- and inter-day precision and accuracy.

Nominal,	Intra-day (n = 6)				Inter-day (n = 18)			
ng/mL	10.0	30.0	200	800	10.0	30.0	200	800
mean, ng/mL	9.97–10.2	30.1–32.7	202–217	828–853	10.1	31.5	211	842
RSD,%	4.8–8.3	2.4–4.0	2.2–3.6	1.2–3.3	6.2	4.6	4.2	2.7
%dev	(-0.32)-2.3	0.4–9.1	0.8–8.5	3.5–6.6	0.6	5.1	5.5	5.3

Absolute matrix effect was evaluated with mean peak areas from Set 1 (clean solution) and Set 2 (post-extraction spiked solution). A value of 100% indicated no matrix effect. If the value was >100%, ion enhancement was observed, and if <100%, ion suppression was observed. At low, medium, and high concentrations, the matrix effect for PQ was 107, 86.6, and 91.8%, respectively (Table 3). The IS normalized matrix effect (PQ/IS) was close to 100%. These results indicated that the matrix effect was not significant and compensated well by the deuterated IS.

**Table 3:** Matrix effect and recovery (n = 7).

Conc	PQ Peak Area, x10^4^			IS Peak Area, x10^4^			Matrix Effect			Recovery	
(ng/ml)	Set 1	Set 2	Set 3	Set 1	Set 2	Set 3	PQ	IS	PQ/IS	PQ	IS
Low (30)	4.13±1.38	4.40±0.62	3.01±0.43	4.30±0.15	4.26±0.36	3.28±0.33	107	99.1	108	68.4	77.0
Med (200)	29.0±2.5	25.1±2.7	21.4±1.2	4.41±0.36	3.88±0.42	3.23±0.23	86.6	88.0	98.4	85.5	83.3
High (800)	110±3	101±6	878±7	4.15±0.12	3.66±0.35	3,24±0.29	91.8	88.2	104	87.3	88.6

Set 1, MeOH-Water (3:1, v/v) solution; Set 2, post extraction spiked solution; Set 3, pre-extraction spiked solution.

The recovery of PQ at 30, 200, and 800 ng/mL was 68.4, 85.5, and 87.3% respectively. The mean recovery for the I.S. was 83.0%.

To determine dilution integrity, we diluted an extra high QC (3000 ng/mL) by 3-, 5-, and 10-fold. The accuracy (% deviation) for dilution samples ranged from -4.4 to 2.2 (S2 Table). We also tested partial volumes by taking half volume samples mixed with an equal volume of blank plasma. The % deviation for all partial volume samples ranged from 1.3 to 9.9 (S2 Table). These results confirm that plasma samples can be diluted by up to 10-fold.

Previously, we reported that PQ was stable in plasma at room temperature (21–24°C) for 9 days, at -70°C for 8 months, and after 3 freeze-thaw cycles [3]. In this report, we further tested and found that PQ is stable in plasma after 5 freeze-thaw cycles and at -70°C for at least 21 months. There was no difference in results for plasma samples prepared in glass versus plastic vials, likely due to PQ bound to plasma proteins. The processed samples were stable in autosampler plastic vials for at least 7 days, and stock solution was stable at -70°C for at least 3 years (Table 4). Noticeably, evaluation of stock solution stability with the API2000 yielded large variation from repeated injections; this is most likely due to the metal injection needle used for the API 2000 system. When switched to an Acquity UPLC-PDA system with a polyetheretherketone (PEEK) sample needle, reproducible results were obtained.

**Table 4:** Stability of PQ. Data represent mean (± SD).

Conditions	Untreated***	treated	% remained	n
Plasma, glass container, 4°C, 16 hr				
30 ng/mL	29.8±2.3	30.2±1.1	101	3
800 ng/mL	796±4	832±14	105	3
Autosampler plastic vials, 21–25°C, 7 days				
30 ng/mL	32.8±1.7	29.4±1.1	89.6	4
800 ng/mL	865±27	857±19	99.1	4
Five freeze-thaw cycles				
30 ng/mL	31.7±1.3	30.9±1.2	97.5	3
800 ng/mL	844±12	843±12	99.9	3
Plasma, -70°C, 21 months*				
3 ng/mL	2.76±0.15	2.78±0.04	101	3
200 ng/mL	200±6	209±5	104	3
Stock, -70°C, 40 months**				
	8170±605	8091±338	99	3

* Measured with our previously published method

**measured with UPLC-PDA with peek tubing injection needle.

*** untreated samples were prepared fresh in plastic vials.

To test potential concomitant drug interference, lumefantrine, artemether, dihydroartemisinin, nevirapine, efavirenz, zidovudine, lamivudine, stavudine (D4T), lopinavir, nelfinavir, indinavir, saquinavir, and amprenavir were spiked into the medium QC samples. The differences of the spiked samples compared to the control medium QC were within 5%, confirming no interference from potential concomitant drugs (S3 Table).

To further validate the API2000 method, we re-analyzed 116 samples that were analyzed with the previously published method [3]. The concentration of those samples ranged from 9.06 to 553 ng/mL. Three samples were measured as below LLOQ. Among the 113 quantifiable samples, 100 samples (88.5%) were within 20% of the reference value (S4 Table).

### 3. Carryover

Having 6 nitrogen atoms with different pKa values, PQ can be found in multiple forms in solution. Due to this property, peak tailing and carryover peaks were often observed during the analysis of PQ. The majority of published LC-MS/MS methods utilized acidic mobile phase solvents [3, 9–12], while two methods used basic mobile phase solvents [8, 23] (Table 5). Carryover peaks were observed in nearly all published LC-MS/MS methods. Most methods maintained the carryover peaks within 20% LLOQ. In the method published by Hodel et al, the carryover peak of PQ was >100% LLOQ [10], partially due to the wide calibration range. Recently, our group published a method on an API 5000 LC-MS/MS system [3] in which carryover from the column was removed, but carryover from the autosampler/injection was still present at ~0.08% of ULOQ (250 ng/mL). Accordingly, the LLOQ was raised to 1.5 ng/mL. In the modified method with a calibration range of 0.5-50ng/mL, the residual carryover peak was maintained within 20% LLOQ (S3 File). Our group also developed a method using a basic mobile phase [10], based on a method published by Lindegardh et al [2]. The peak tailing and carryover were significant (S3 File). In the current report, we changed the basic additive from 2.5 mM NH_4_HCO_3_ to 10 mM NH_4_OH, and changed the column from Zorbax Eclipse C_18_ (50x2.1mm, 5μm) to Gemini C_18_ (50x2.0, 5μm). With these changes, the carryover peak was reduced markedly. Although carryover peak was observed in some runs, in most cases no carryover peaks were observed with this method (S4 File). It might possibly be due to the less sensitivity of the instrument and/or the narrow calibration range spanning only 100-fold when compared to other methods. It is also likely due to the basic mobile phase condition. We speculate that the ionic PQ molecule causes the carryover, which could diminish at basic condition where PQ is uncharged. Considering PQ pka = 8.6, PQ will be uncharged if mobile phase pH >10.6, and analytical columns applicable at pH 11 or higher are now available with the advancement of column technology.

**Table 5:** Published methods for PQ quantification.

Reference	Instrument	Column	Mobile phase	Sample preparation	Calibration range	Carryover	Retention factor k
Hodel et al, 2009	TSQ Quantum (ESI+)	Atlantis dC18 (50x2.1mm)	20mM NH_4_FA 0.5% FA; MeCN 0.5%FA	PPT: 200uL plasma+700uL MeCN	2–4000 ng/mL	yes (>100% LLOQ)	9.7
Singhal et al, 2007	API4000 Q-trap (ESI+)	Chromolith SpeedROD RP-18e (50x4.6mm)	NH_4_AC-MeOH-FA-NH_3_	PPT: 50uL plasma+300uL MeOH	1–250 ng/mL	yes (not specified)	0.6
Lindegardh, et al, 2008	API5000 (ESI+)	Gemini C18 (50x2.0mm)	NH_4_HCO_3_-MeCN	SPE: 50 uL plasma	1.5–500 ng/mL	<15%LLOQ	2.4
Lee et al, 2011	API2000 (ESI+)	Zorbax C18 (50x2.1mm, 5um)	NH_4_HCO_3_-MeCN	Dilution: 50uL PBS samples +IS	20-1000ng/mL	yes (18–125%LLOQ) 4	
Kjellin, et al, 2014	API5000 (APCI+)	Pursuit PFP (50x2.0mm, 3um))	NH_4_FA-TFA-MeCN	PPT: 25uL plasma+100uL MeOH-TCA	1.5-250ng/mL	<20%LLOQ	4
Liu et al, 2017	API5500 Q-trap (ESI+)	Venusil XBP-C18 (50x2.1mm, 5um)	2mMNH4AC 0.15%FA 0.05%TFA-MeCN	PPT: 40uL plasma+HCl+160 uL MeCN	2–400 ng/mL	≤LLOQ	3
Aziz et al, 2017	API4000 (ESI+)	Express C18 (30x2.1mm, 5um)	0.1%FA in Water-MeCN	PPT: 100uL plasma+ 300uL 1%FA in water	3.9–2508 nM	Yes (not specified)	29
This report	API5000 (APCI+)	Pursuit PFP (50x2.0mm, 3um)	NH_4_FA-TFA-MeCN	PPT: 25uL plasma+100uL MeOH-TCA	0.5-50ng/mL	<20%LLOQ	4
This report	API2000 (ESI+)	Gemini C18 (50x2.0mm,5um)	10mMNH_4_OH-MeCN	PPT: 25uL plasma+25ul IS+150uL MeOH	10–1000 ng/mL	no	6

### 4. Analysis of clinical samples

A total of 215 pairs of plasma samples simultaneously collected from capillary and venous blood were analyzed. Five pairs of samples were below LLOQ (0.5ng/mL) for both capillary and venous PQ, 4 capillary samples were below LLOQ with measurable venous PQ, and 1 venous sample was below LLOQ with measurable capillary PQ, yielding a total of 205 pairs of quantifiable data points for correlation analysis.

### 5. Correlation of PQ in capillary versus venous plasma

**Fig 3:**
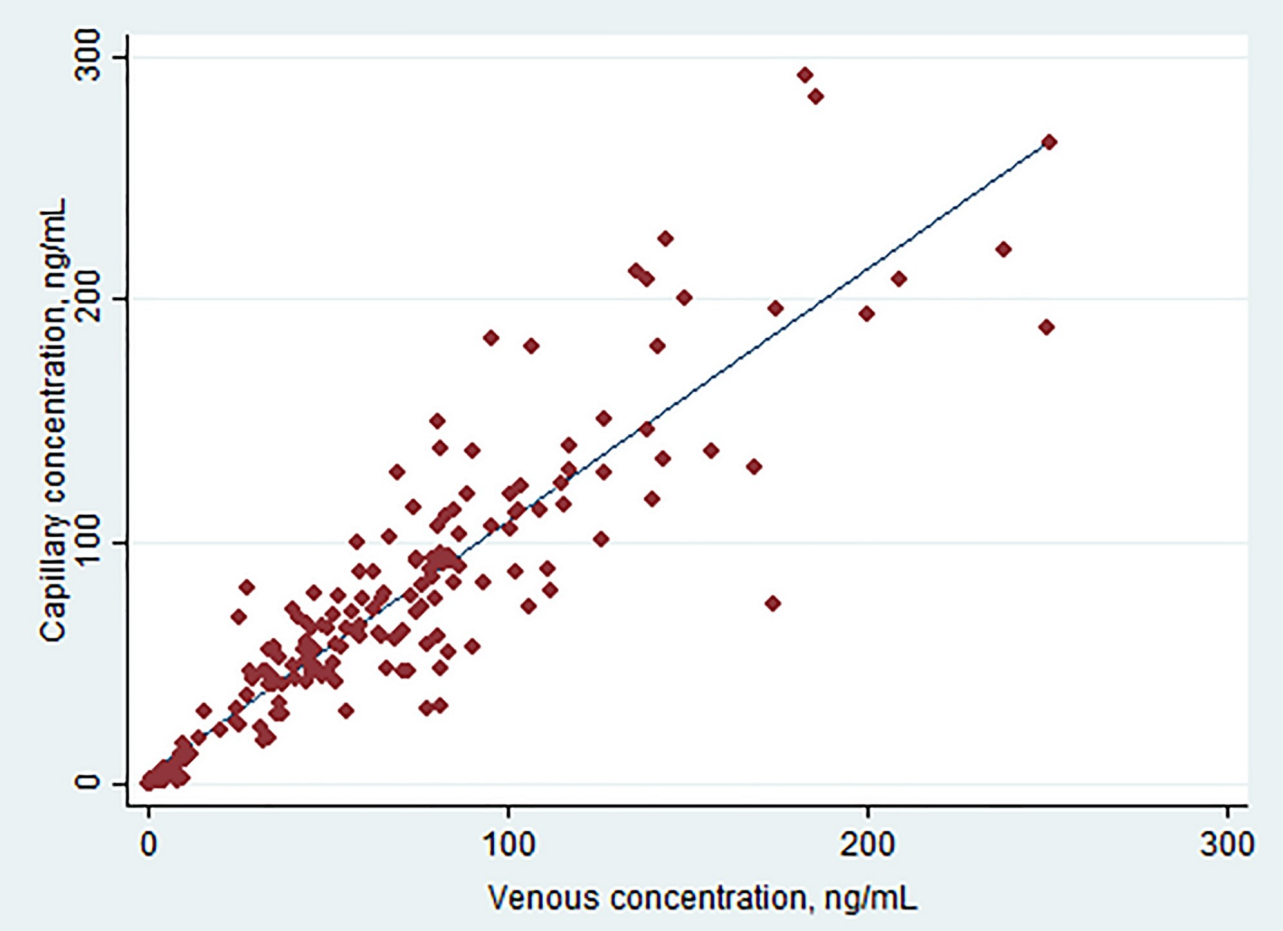
Linear regression of capillary versus venous plasma PQ.

A total of 205 pairs of data above LLOQ were obtained from samples collected from 24 hr to 82 days post last dose in children, pregnant women and non-pregnant adults. Simple linear regression yields an equation: C_cap_ = 1.04×C_ven_ + 4.20, R^2^ = 0.832 (Table 6). The 95% confidence intervals (CI) of the intercept included zero (-0.878, 9.27), and the p-value was 0.105, suggesting the difference from zero was not statistically significant. The slope was 1.04 with 95% CI (0.978, 1.11). The results suggest PQ concentrations in capillary and venous plasma appear to be correlated in a simple linear relationship. However, large variation led to a scattered correlation plot (Fig 3), a significant portion of samples could not be explained by the linear equation, making extrapolation of PQ concentrations from capillary to venous plasma complicate. With natural log-transformed data, improved correlation was obtained with R^2^ values of 0.945 and the equation lnC_cap_ = 1.01×lnC_ven_ + 0.013. In general, slightly higher PQ concentrations were found in capillary samples, with a median value of 56.3 ng/mL versus 50.4 ng/mL in venous samples (p<0.0001). The mean ratio (±SD) of C_cap_/C_ven_ was 1.13±0.42, and median with interquartile range (IQR) was 1.08 (0.917, 1.33). However, 78 of 205 capillary PQ values (38%) were lower than corresponding venous PQ values.

**Table 6:** Correlation of capillary and venous PQ plasma concentrations.

	Total (n = 205)	24 hr post last dose (n = 150)	≥ 7 days post dose (n = 55)
C_ven_, ng/mL	50.4 (0.504, 251)	70.8 (9.67, 251)	3.41 (0.504, 20.4)
C_cap_, ng/mL	56.3 (0.584, 292)	73.5 (16.7, 292)	2.84 (0.584, 22.6)
P value	<0.0001	<0.0001	0.38
Correlation equition: C_cap_ = a × C_ven_ + b			
a	1.04 (0.978, 1.11)	0.984 (0.886, 1.08)	0.965 (0.828, 1.10)
b	4.20 (-0.878, 9.27)	10.6 (1.68, 19.5)	-0.182 (-1.02, 0.661)
R^2^	0.832	0.729	0.789

Concentrations represent medians (range) and correlation parameters represent means (95%CI).

Considering the impact of pharmacokinetic stages, we performed sub-analysis with data from 24 hr post last dose and ≥ 7 days post dose. At 24 hr post last dose, we collected 150 pairs of data, 57 from pregnant women, 63 from children under 2 years old, and 30 from non-pregnant women. The correlation equation at this time interval in each population was published previously with natural log-transformed data, which yielded better correlation than non-transformed data [13, 14]. When all data at 24 hr post last dose were combined (n = 150), simple linear regression yielded a coefficient of determination (R^2^) of 0.729 (Table 6). We observed a slightly higher capillary PQ concentration on average, with the median value of 73.5 ng/mL versus 70.8 ng/mL for venous PQ (p<0.0001). The mean ratio (±SD) of C_cap_/C_ven_ was 1.17±0.37, and median (IQR) was 1.12 (0.952, 1.34). The data suggest that the capillary concentration is slightly higher at the distribution phase. However, 48 of the 150 capillary PQ concentrations (32%) were lower than venous PQ concentrations. Natural log-transformed data did not improve the correlation. At ≥ 7 days post dose, we collected 55 pairs of data from children, yielding a linear regression equation C_cap_ = 0.965×C_ven_− 0.182 and R^2^ = 0.789. Log-transformed data did not improve the correlation. The median PQ concentrations for venous and capillary plasma were 3.41ng/mL and 2.84ng/mL, respectively, and the difference was not statistically significant (p = 0.38). The mean ratio (±SD) of C_cap_/C_ven_ was 1.03±0.52, and median (IQR) ratio was 0.974 (0.793, 1.21). Over 50% of capillary PQ concentrations were lower than venous PQ concentrations, suggesting that at the terminal elimination phase venous PQ tends to be higher than capillary PQ. A study in Burkina Faso reported day 7 PQ concentrations in capillary plasma were higher than those in venous plasma with median (range) concentration at 67 (49–84) in capillary versus 41 (27–59) in venous plasma (n = 186, p<0.001) [24]. Our result is different, likely because 54 of the 55 pairs of samples were collected beyond 20 days after dose, which are better representatives of the terminal elimination phase.

A study in Thailand estimated correlation of dihydroartemisinin-piperaquine administration on malaria patients aged >2 years [17]. Venous blood PQ were nearly always higher than venous plasma PQ with a median (90% range) ratio of 2.15 (0.91, 5.26), suggesting PQ concentrated in red blood cells; while the difference between capillary blood and venous blood PQ concentrations was smaller with median (90% range) ratio of 1.66 (0.92, 3.03). After day 3 when parasitaemia had cleared, a simple relationship was found: the venous blood PQ concentration = (capillary blood PQ concentration)^0.9^. However, similar to what we observed here, there were also large variations in that study, leading to the conclusion that measurements of venous and capillary PQ concentration are not readily interchangeable. In contrast, another antimalarial, lumefantrine, showed good linear correlation at a 1:1 ratio between capillary and venous samples, with coefficient of determination R^2^>0.95 [21]. This is probably due to the complex pharmacokinetic profile of PQ, such as multiple peak concentrations, slow distribution of PQ requiring longer time to reach equilibrium, large distribution volume (up to 874L/kg), and long elimination half-life (up to 28 days) [25]. It is likely not feasible to find a linear correlation during early pharmacokinetic phases, as equilibrium between different compartments is not reached and varied among individuals. Correlation may be better accessed during the steady state and the elimination phase., e.g day 14 or 28.

## Conclusions

We reported a method for quantitation of PQ in plasma with a calibration range of 10–1000 ng/mL. Carryover was negligible in the method.

The concentrations of PQ in capillary and venous plasma were correlated in a linear relationship. However, due to large variations, exchange of PQ concentrations between capillary and venous plasma will compromise precision of the results. Correlation study accounting for disposition phases may be necessary.

## Supporting information

S1 Table Adsorption of PQ on container surface. (XLSX)

S2 Table Dilution and partial volume precision and accuracy for PQ. (XLSX)

S3 Table Interference of potential concomitant drugs. (XLSX)

S4 Table Cross validation using the previous API5000-based method as reference. (XLSX)

S1 File Partial validation of the modified PQ assay. (PDF)

S2 File (DTA)

S3 File (PDF)

S4 File (PDF)

## References

[1] WHO. Guidelines for the treatment of malaria. Third edition. https://www.who.int/malaria/publications/atoz/9789241549127/en/2015 [cited 2019 August 25]. 2015:[Available from: https://www.who.int/malaria/publications/atoz/9789241549127/en/.

[2] Bigira V, Kapisi J, Clark TD, Kinara S, Mwangwa F, Muhindo MK, et al Protective efficacy and safety of three antimalarial regimens for the prevention of malaria in young Ugandan children: a randomized controlled trial. PLoS Med. 2014;11(8):e1001689 10.1371/journal.pmed.1001689 25093754 PMC4122345

[3] Kjellin LL, Dorsey G, Rosenthal PJ, Aweeka F, Huang L. Determination of the antimalarial drug piperaquine in small volume pediatric plasma samples by LC-MS/MS. Bioanalysis. 2014;6(23):3081–9. 10.4155/bio.14.254 25529877 PMC4321809

[4] EMA. Eurartesim Assessment Report 2011 [cited 2019 February, 6]. Available from: https://www.ema.europa.eu/documents/assessment-report/eurartesim-epar-public-assessment-report_en.pdf.

[5] Hung TY, Davis TM, Ilett KF. Measurement of piperaquine in plasma by liquid chromatography with ultraviolet absorbance detection. Journal of chromatography B, Analytical technologies in the biomedical and life sciences. 2003;791(1–2):93–101. Epub 2003/06/12. 10.1016/s1570-0232(03)00209-5 . 12798169

[6] Lindegardh N, Ashton M, Bergqvist Y. Automated solid-phase extraction method for the determination of piperaquine in plasma by peak compression liquid chromatography. J Chromatogr Sci. 2003;41(1):44–9. 10.1093/chromsci/41.1.44 . 12597597

[7] Lindegardh N, White NJ, Day NP. High throughput assay for the determination of piperaquine in plasma. J Pharm Biomed Anal. 2005;39(3–4):601–5. 10.1016/j.jpba.2005.03.031 . 15905062

[8] Lindegardh N, Annerberg A, White NJ, Day NP. Development and validation of a liquid chromatographic-tandem mass spectrometric method for determination of piperaquine in plasma stable isotope labeled internal standard does not always compensate for matrix effects. Journal of chromatography B, Analytical technologies in the biomedical and life sciences. 2008;862(1–2):227–36. 10.1016/j.jchromb.2007.12.011 . 18191623

[9] Singhal P, Gaur A, Gautam A, Varshney B, Paliwal J, Batra V. Sensitive and rapid liquid chromatography/tandem mass spectrometric assay for the quantification of piperaquine in human plasma. Journal of chromatography B, Analytical technologies in the biomedical and life sciences. 2007;859(1):24–9. 10.1016/j.jchromb.2007.09.021 . 17923446

[10] Hodel EM, Zanolari B, Mercier T, Biollaz J, Keiser J, Olliaro P, et al A single LC-tandem mass spectrometry method for the simultaneous determination of 14 antimalarial drugs and their metabolites in human plasma. Journal of chromatography B, Analytical technologies in the biomedical and life sciences. 2009;877(10):867–86. 10.1016/j.jchromb.2009.02.006 . 19249251

[11] Liu H, Zang M, Yang A, Ji J, Xing J. Simultaneous determination of piperaquine and its N-oxidated metabolite in rat plasma using LC-MS/MS. Biomed Chromatogr. 2017;31(10). 10.1002/bmc.3974 . 28299804

[12] Aziz MY, Hoffmann KJ, Ashton M. LC-MS/MS quantitation of antimalarial drug piperaquine and metabolites in human plasma. Journal of chromatography B, Analytical technologies in the biomedical and life sciences. 2017;1063:253–8. 10.1016/j.jchromb.2017.06.035 . 28863865

[13] Kajubi R, Huang L, Jagannathan P, Chamankhah N, Were M, Ruel T, et al Antiretroviral Therapy With Efavirenz Accentuates Pregnancy-Associated Reduction of Dihydroartemisinin-Piperaquine Exposure During Malaria Chemoprevention. Clin Pharmacol Ther. 2017;102(3):520–8. 10.1002/cpt.664 28187497 PMC5546920

[14] Whalen ME, Kajubi R, Chamankhah N, Huang L, Orukan F, Wallender E, et al Reduced Exposure to Piperaquine, Compared to Adults, in Young Children Receiving Dihydroartemisinin-Piperaquine as Malaria Chemoprevention. Clin Pharmacol Ther. 2019 10.1002/cpt.1534 . 31173649 PMC6851416

[15] Mohammed BS, Cameron GA, Cameron L, Hawksworth GH, Helms PJ, McLay JS. Can finger-prick sampling replace venous sampling to determine the pharmacokinetic profile of oral paracetamol? British journal of clinical pharmacology. 2010;70(1):52–6. Epub 2010/07/21. 10.1111/j.1365-2125.2010.03668.x 20642547 PMC2909807

[16] Gordi T, Hai TN, Hoai NM, Thyberg M, Ashton M. Use of saliva and capillary blood samples as substitutes for venous blood sampling in pharmacokinetic investigations of artemisinin. European journal of clinical pharmacology. 2000;56(8):561–6. Epub 2001/01/11. 10.1007/s002280000179 . 11151745

[17] Ashley EA, Stepniewska K, Lindegardh N, Annerberg A, Tarning J, McGready R, et al Comparison of plasma, venous and capillary blood levels of piperaquine in patients with uncomplicated falciparum malaria. European journal of clinical pharmacology. 2010;66(7):705–12. Epub 2010/03/20. 10.1007/s00228-010-0804-7 20300743 PMC2883082

[18] CPQA. Clinical pharmacology quality assurance (CPQA) guidelines for bioanalytical chromatographic method development, validation, and application, v1.0. Apri 1, 2017.

[19] FDA. US Food and Drug Administration (FDA) Guidance for Industry–Bioanalytical Method Validation (BMV), May 2001.

[20] Wallender E, Vucicevic K, Jagannathan P, Huang L, Natureeba P, Kakuru A, et al Predicting Optimal Dihydroartemisinin-Piperaquine Regimens to Prevent Malaria During Pregnancy for Human Immunodeficiency Virus-Infected Women Receiving Efavirenz. J Infect Dis. 2018;217(6):964–72. 10.1093/infdis/jix660 29272443 PMC5853566

[21] Savic RM, Jagannathan P, Kajubi R, Huang L, Zhang N, Were M, et al Intermittent Preventive Treatment for Malaria in Pregnancy: Optimization of Target Concentrations of Dihydroartemisinin-Piperaquine. Clin Infect Dis. 2018;67(7):1079–88. 10.1093/cid/ciy218 29547881 PMC6137112

[22] Kakuru A, Jagannathan P, Muhindo MK, Natureeba P, Awori P, Nakalembe M, et al Dihydroartemisinin-Piperaquine for the Prevention of Malaria in Pregnancy. N Engl J Med. 2016;374(10):928–39. 10.1056/NEJMoa1509150 26962728 PMC4847718

[23] Lee TM, Huang L, Johnson MK, Lizak P, Kroetz D, Aweeka F, et al In vitro metabolism of piperaquine is primarily mediated by CYP3A4. Xenobiotica. 2012;42(11):1088–95. 10.3109/00498254.2012.693972 22671777 PMC5087332

[24] Zongo I, Some FA, Somda SA, Parikh S, Rouamba N, Rosenthal PJ, et al Efficacy and day 7 plasma piperaquine concentrations in African children treated for uncomplicated malaria with dihydroartemisinin-piperaquine. PLoS One. 2014;9(8):e103200 10.1371/journal.pone.0103200 25133389 PMC4136730

[25] Tarning J, Ashley EA, Lindegardh N, Stepniewska K, Phaiphun L, Day NP, et al Population pharmacokinetics of piperaquine after two different treatment regimens with dihydroartemisinin-piperaquine in patients with Plasmodium falciparum malaria in Thailand. Antimicrob Agents Chemother. 2008;52(3):1052–61. 10.1128/AAC.00955-07 18180343 PMC2258541

