# Supplementary material for: Determination of piperaquine concentration in human plasma and the correlation of capillary versus venous plasma concentrations
Source: PLoS One. 2020 May 29;15(5):e0233893. doi: 10.1371/journal.pone.0233893 (PMC7259774; doi:10.1371/journal.pone.0233893)
Supplement: S1 File — (PDF) [file pone.0233893.s005.pdf]

Determination of Piperaquine in Human EDTA Plasma by LC-MS/MS on API5000
Assay Modified for Lower Calibration Range

Assay Validation Report

Version 1.1
December 2, 2015

Vincent Cheah, B.S.

Liusheng Huang, Ph.D.

Summary of changes

This method with a lower calibration range of 0.5-50ng/mL is a modification of the previous CPQA approved LC/MS/MS method: both methods use the same sample preparation and API5000-LC-MS/MS system; both utilize PFP-based analytical column and the same mobile phase solvents.

In the modified method, calibrators consists of 0.5, 1.0, 1.5, 5, 10, 25, 50 ng/mL, and the low, medium, and high QC levels are 1.5, 20, and 40 ng/mL, respectively.

The changes are summarized in table 1

Table 1. Comparison of the two LC-MS/MS methods for determination of piperaquine.

	New method	Previous approved method
LLOQ	0.5ng/mL	1.5ng/mL
ULOQ	50 ng/mL	250 ng/mL

Determination of Piperaquine in Human Plasma by LC-MS/MS on API5000
Assay Modified for Lower Calibration Range

Summary of Assay Specifications

Drug: Piperaquine Formula $C_{29}H_{32}Cl_2N_6{\cdot}4H_3PO_4{\cdot}4H_2O$ MW: 999.55
MW (base form):535.52; EM (base form): 534.21
Other names: 4,4'-(1,3-propanediyldi-4,1-piperazinediyl)bis[7-chloro-quinoline], Piperaquinoline. CAS# 4085-31-8
AK Scientific, Inc. – Union City, CA. Lot #70101L (99%)

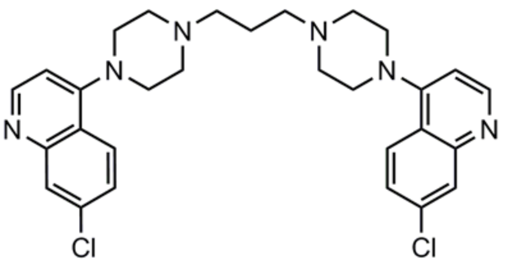

Piperaquine

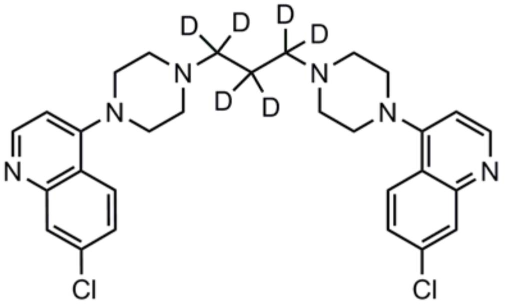

Piperaquine-d6

Internal Standard: piperaquine-d_6 Formula $\mathbf{C_{29}H_{26}D_6Cl_2N_6}$ MW: 541.55 EM: 540.24
Other names: 7-chloro-4-[4-[3-[4-(7-chloroquinolin-4-yl) piperazin-1-yl]-1,1,2,2,3,3-hexadeuteriopropyl]piperazin-1- yl]quinolone, piperaquinoline-d_6, PQ-d_6.
AlSAchim, SAS, IllKirch, France. Batch#JA-ALS-10-077, CAS# 1261394-71-1, Pale yellow.
Chemical purity, >99%; Isotopic purity, ≥99%.

Biological Matrix: Human plasma (EDTA as anticoagulant) Sample size 25 μL

Method of Assay: Reverse phase HPLC with tandem mass spectrometer as the detector ($APCI^+$, MRM).

Method of Integration: Peak area ratios Regression: Linear weighted by 1/x

Method of Sample Preparation: Protein precipitation with 50% methanol 5%TCA.

Range of Calibration Curve: 0.50-50.0ng/mL

Inter-assay precision	Conc. piperaquine (ng/mL)	CV%	
	1.50	6.4	
	20.0	5.1	
	40.0	4.8	(n =18 for each conc.)

LOQ 0.500ng/mL (CV % 8.1)

Intra-assay precision	Conc. piperaquine (ng/mL)	CV% [range]
	1.50	2.11-6.85
	20.0	2.93-5.67
	40.0	3.50-4.08

LOQ 0.500ng/mL (CV % 3.98-11.15)

Mean recovery of drug from human plasma: 91%

Storage stability at –70 °C: 21 months

Determination of Piperaquine in Human Plasma by LC-MS/MS on API5000
Assay Modified for Lower Calibration Range

Piperaquine (PQ) is an antimalarial drug used in preventive treatment and combination therapy, because of its long elimination half life ($t_{1/2}$ = 7days based on our unpublished data). It is a hydrophobic aromatic heterocyclic compound with multiple nitrogen atoms. PQ is not soluble in water and most organic solvents but soluble in acidified solvents[1].

Previously we developed a method to quantify PQ with API5000 LC-MS/MS system. The assay uses deuterated PQ (PQ-d_6) as the internal standard (IS), and 25 µL plasma sample was used for this analysis**.** The LLOQ could reach 0.5ng/mL. However, due to carryover issue, the LLOQ increased to 1.5ng/mL and calibration range was 1.5-250ng/mL[2]. During our initial analysis of 380 clinical samples from a malaria chemoprevention study with dihydroartemisinin-piperaquine, 76 samples (20%) were below the LLOQ 1.5ng/mL, while only one sample was above 50ng/mL. Thus we determine to modify the method to a new calibration range of 0.5-50ng/mL to support this specific study.

According to CPQA guidelines[3], a partial validation should be performed if the concentration range is changed. The required validations are as follows: 1), three inter-/intra-day precision and accuracy, including the new LLOQ in the validation QC samples. 2), Matrix effect and recovery.

Principle of the Method

PQ and the internal standard (IS) PQ-d_6 are extracted from EDTA plasma by protein precipitation with 5% TCA in MeOH-water (1:1, v/v). The processed sample is injected onto a Pursuit PFP column (2.0 $\times$ 50 mm, 3 µm) eluted with 20 mM NH_4FA 0.14% TFA (pH 2.96) and acetonitrile with 0.1% TFA in a gradient mode. APCI and multiple reaction monitoring (MRM) were used, and ion pairs 535/288 for PQ and 541/294 for the IS were selected for quantification, and 535/260 for PQ was used for confirmation. The retention times are typically 1.0(±0.2) min for PQ and 1.0(±0.2) min for the IS. Total run time is 3.0 min per sample. The LC-MS/MS system is operated at room temperature (25 ± 2 °C controlled by an air conditioner). Calibration curve standards and quality controls are prepared in blank EDTA human plasma from separately weighted and prepared PQ solutions. For calibration curves, spiked concentrations and peak area ratios of PQ to the IS are fitted by linear least squares regression, weighted by 1/x. Plastic sample vials should be used instead of glass due to absorption of PQ to glass surface.

PARTIAL VALIDATION

Calibration Curve

Calibration standards, prepared in EDTA human plasma, consisting of 0.500, 1.00, 1.50, 5.0, 10.0, 25.0 and 50.0 ng/mL of PQ, were used to establish the calibration curve for assay validation. A single curve was plotted using concentration vs. mean peak area ratio obtained by injecting a set of calibrators in the beginning of the batch run. Linear least squares regression with 1/ x weighting of the calibration plot resulted in correlation coefficients [r] greater than 0.995 for all three assays [Table 1]. The mean precision and percent deviation of the calibrations standards over 3 days, ranged from 2.4-14.6 % and (-8.0)-7.3%, respectively [Table 1]. A representative calibration curve is shown in Figure 1. Representative chromatograms for blank plasma, the lower limit of quantification (LLOQ), and blank plasma after ULOQ are shown in Figure 2.

Inter-assay (between day) and Intra-assay (within day) Precision and Accuracy

Precision is the degree of reproducibility; it characterizes the degree of agreement among a series of individual measurements. Precision is calculated as the coefficient of variation (%CV). Accuracy is the degree of correctness and is expressed as the percent deviation from the true concentration value. For inter-assay precision and accuracy, 6 replicates of validation samples, made from the same homogeneous matrix volume, at each of 3 different drug concentrations were analyzed on 3 separate days. A fresh calibration curve was used each day. The validation samples were made up with PQ concentrations of 1.50 ng/mL, 20.0 ng/mL, and 40.0 ng/mL. These were designated low, median, and high, respectively.

The inter-assay precision (CV %) of this method for PQ was 6.4, 5.1and 4.8 % for low, median, and high concentrations respectively. The overall accuracy (%dev) from nominal concentration value was 6.4,(-1.1), and 2.6% for low, median and high concentrations respectively [Table 2].

Intra-assay precision and accuracy were calculated from 6 replicate samples of low, median, and high concentrations analyzed on the same day for 3 unique days. The precision (%CV) of this method for low, median, and high concentrations ranged from 2.11-6.85%, 2.93-5.67%, and 3.50-4.08% and accuracy (percent deviation) ranged from 1.30-12.2%, (-5.7)-1.67% and 0.21-7.25%, respectively [Table 2].

Lower Limit of Quantification

Six replicates of validation samples at the lowest calibration concentration (0.500 ng/mL) were analyzed on 3 days to determine the inter- and intra-assay precision and accuracy of this lowest point on the calibration curve. The inter-assay precision (%CV) was 8.1 % and percent deviation was 6.8 %. The intra-assay %CV for the mean of these 3 replicate days ranged from 3.98-11.1% and the mean accuracy (%dev) from 2.30-10.0% [Table 2].

Matrix Effect, Recovery, and Process Efficiency

Matrix effect

According to CPQA guidelines Appendix F, five different lots of blank EDTA human plasma (#00023-35504, #00023-35491, #0002335479, #LS23-73766, #LS2356244) were used for matrix effect test. To evaluate matrix effect of hemolized plasma, 2 aliquots of plasma spiked with 1% and 5% whole blood (LS2356244 with 1% blood and LS2356244 with 5% blood) freeze and thaw for 2 cycles to lyse the red blood cells. Then matrix effect experiments were performed with the 5 normal plasma lots.

Four sets of samples at three concentration levels (low, median and high QC) were prepared and analyzed to determine matrix effect, recovery, and process efficiency.

Set 1a: Un-processed PQ without matrix and IS (drugs only): 10 µL PQ [6, 80, or 160ng/mL in ACN-water (1:1, v/v) with 0.5%FA] was spiked into 190 µL water-MeOH (1:1/v/v) containing 5%TCA. The final PQ concentration was 0.3ng/mL, 4ng/mL, and 8ng/mL for low, median and high QC levels, respectively. In addition, 10 µL PQ-d_6 [8ng/mL in ACN-water (1:1, v/v) with 0.5%FA] was spiked into 190 µL µL water-MeOH (1:1/v/v) containing 5%TCA to give final IS concentration at 0.4ng/mL.
Set 1b: Un-processed PQ without matrix but with IS (PQ-d_6): 10 µL PQ (6, 80, or 160ng/mL) and 10 µL PQ-d_6 (8ng/mL) were spiked into 180 µL water-MeOH (1:1/v/v) containing 5%TCA.
Set 2: Post extraction spike into extracted blank plasma (defines absolute and relative matrix effect). Each of the 7 lots of blank plasma samples (150 µL) was mixed with 600 µL water-MeOH (1:1/v/v) containing 5%TCA, vortexed for 10s and centrifuged for 3min at 25,000g. Aliquots of 180 µL supernatant were taken and spiked with PQ and IS (PQ-d_6) (10 µL each) to make the final concentration equal to those in set 1b.
Set 3: Pre extraction spike into plasma then extract (defines recovery and overall process efficiency). PQ was spiked into plasma at 1.5ng/mL, 20ng/ml, and 40ng/mL in each of 7 lots of plasma, then extracted as described in SOP.

Set 1a and 1b consist of 7 replicates of injections at each concentration. Set 2 and 3 consist of 7 different sources of plasma at each concentration level. Injection was performed in the order of set1a-L1, set1b-L1, set2-L1, set3-L1, then repeat the order for matrix #2, 3, 4, 5, 6, and 7. Repeat the process for median- and high-validation samples. Data and comparisons are presented in table 3A-3D.

Relative matrix effect of the method for PQ and the I.S. can be evaluated by comparing the %CV from set 1b and set 2 (Table 3A). The differences between CV% of peak areas from set 1b and 2 are -0.1, -3.3, and -1.7 at low, median, and high concentration levels, respectively; the corresponding values for IS are 0.2, -3.7, and -1.0, respectively. When comparing CV% from the peak area ratios (H-G), these values are 0.3, 0.1, and 0.9, all within 5%, suggesting that IS compensated for the variation. These results suggest no significant relative matrix effect.

Absolute matrix effect was evaluated with mean peak area values from set 1 and 2. A value of 100% means no matrix effect. At low, median, and high concentration levels, the matrix effect

for PQ is 122, 120, and 117%, respectively. However, The IS exhibits the same trend of matrix effect (122, 112, and 114%, respectively). The difference (H-G) is less than 8% (Table 3B), suggesting IS compensates matrix effect for PQ. These results indicate that matrix effect in the method is well compensated by the deuterated IS. [Table3B].

Furthermore, slopes of lines connecting low, median, and high samples from each lot of plasma were calculated. The CV% from set 3 is 2.88% (<5%), confirming absence of significant matrix effect on quantification. [Table3C].

In summary, matrix effect on quantification for this assay is not significant and compensated well by the deuterated internal standard PQ-d6. Hemolyzed plasma won't affect quantification of PQ either.

Recovery (RE)

The recovery of PQ from plasma following sample preparation was assessed by comparing the peak areas from set 3 and set 2 [Table 3B]. The recoveries for PQ are 107.2, 83.7 and 82.4% at low, median and high concentration, respectively, and the recovery for the IS ranged from 90.8-92.7%. The CV% of peak areas for recovery experiment was all within 10%. These results suggest the assay is highly reproducible across the concentration range. FDA guidelines state that recovery need not be 100% as long as it is consistent, precise, and reproducible.

Recovery, Process Efficiency and Matrix Effect were calculated with the following formulas:

$$\text{Recovery} = \frac{100 \times \text{peak area of pre-extraction spiked sample (set3)}}{\text{peak area of post-extraction spiked sample (set2)}}$$

$$\text{Matrix Effect} = \frac{100 \times \text{peak area of post-extraction spiked sample (set2)}}{\text{peak area of clean sample (set1b)}}$$

$$\text{Process Efficiency} = \frac{100 \times \text{peak area of pre-extraction spiked sample (set3)}}{\text{peak area of clean sample (set 1b)}}$$

Set 1a: un-processed PQ without matrix and IS (defines coelution effect, not necessary in this case because IS will be present in all samples)
Set 1b: Un-processed PQ without matrix but with IS
Set 2: Post-extraction spike into extracted blank plasma (defines absolute and relative matrix effects)
Set 3: Pre-extraction spike into blank plasma and then extracted (defines recovery and overall process efficiency)

Carryover evaluation

In the previously approved method, carryover from autosampler remained at a significant level: ~0.08% of ULOQ (250ng/mL), corresponding to ~0.2ng/mL PQ. To be conservative, the LLOQ for the assay was set at 1.5 ng/mL, meeting the criteria that blank signal should be <20% LLOQ signal. The calibration range was 1.5-250 ng/mL[2].

In this modified assay, since the LLOQ was lowered to 0.5 ng/mL, the ULOQ was lowered to 50 ng/mL accordingly. The residual peak in blank plasma after ULOQ was 17% LLOQ (Figure 2).

To further evaluate the effect of carryover on sample quantification. Calibrator #1 (LLOQ 0.5 ng/mL) was re-injected following calibrator #7 (ULOQ 50ng/mL), the determined concentration of the reinjected calibrator #1 has a mean accuracy (%deviation) of 6.1% and the percent difference from the control (calibrator#1) was 8.7% (Table 4). This result confirmed that carryover in this modified assay won't affect quantification of PQ.

Method

All samples were stored at –70 °C until prepared for analysis. Calibration standards and validation samples were prepared in plasma from separately weighed and prepared PQ solutions. Spiked concentrations and peak area ratios of PQ to IS for the calibration standards were fitted by linear regression with 1/x weighting to the equation. PQ concentrations were calculated from the regression parameters using peak area ratios.

$$\text{Analyte Concentration} = \frac{\text{Peak area ratio [analyte/IS] - y intercept}}{\text{Coefficient of x (slope)}}$$

$$\text{Accuracy (\% Dev)} = \frac{100 \times (\text{Calculated concentration} - \text{nominal concentration})}{\text{Nominal concentration}}$$

Reference:

1. Tarning J, Lindegardh N: Quantification of the antimalarial piperaquine in plasma. *Transactions of the Royal Society of Tropical Medicine and Hygiene* 102(5), 409-411 (2008).
2. Kjellin LL, Dorsey G, Rosenthal PJ, Aweeka F, Huang L: Determination of the antimalarial drug piperaquine in small volume pediatric plasma samples by LC-MS/MS. *Bioanalysis* 6(23), 3081-3089 (2014).

3. ACTG Network Clinical Pharmacology Quanlity Assurance (CPQA) Program: CPQA Guidelines for chromatographic method development and validation based on (and including) FDA guidelines dated May 2001, Version 4.0 (2012). 1-52 (2012).

Figure 1. A Representative Calibration Standard Curve.
Linear regression, 1/x weighted, Y = 0.624X + 0.0388, r = 0.9988

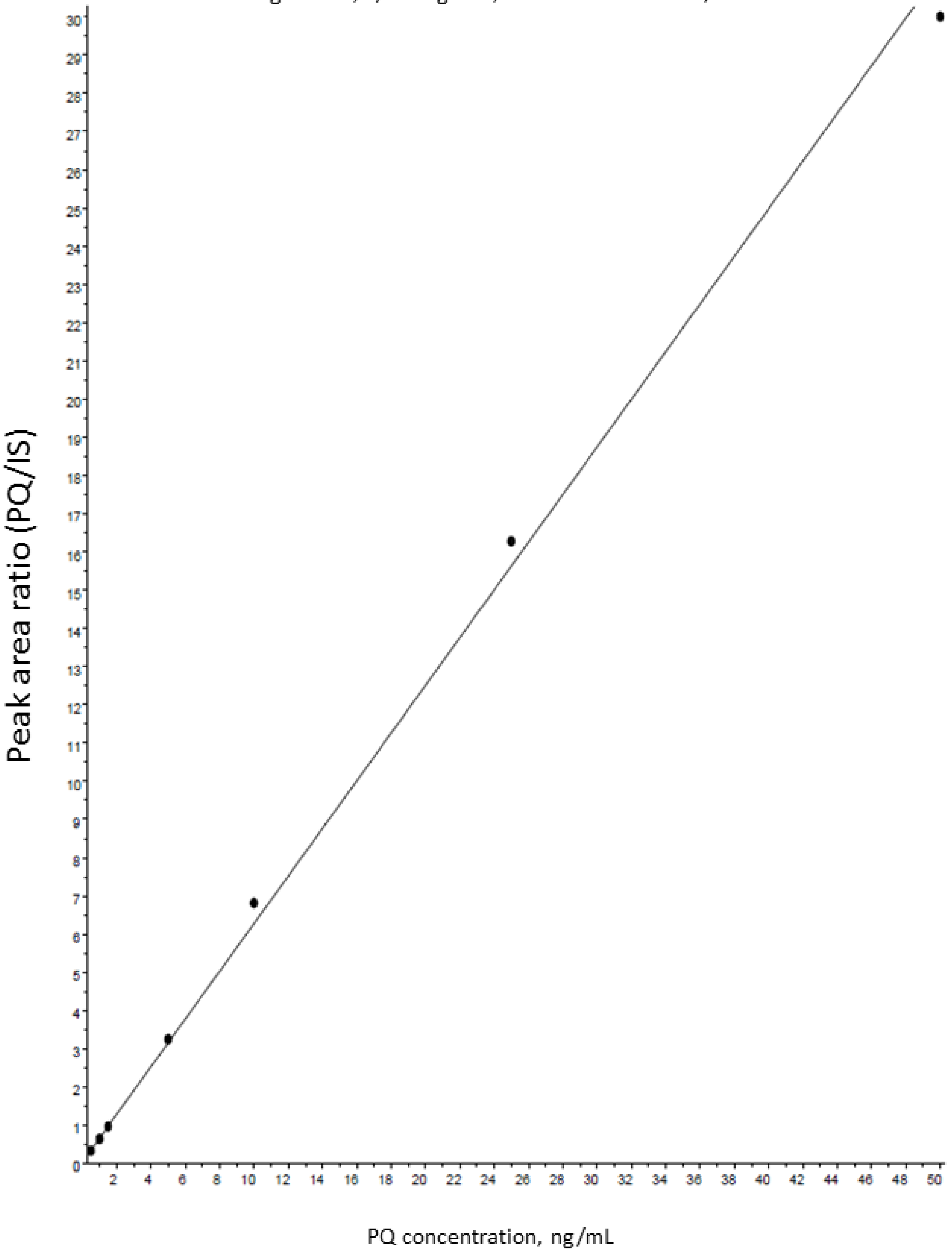

Figure 2. Chromatograms of PQ in a blank plasma (blk), the lower limit of quantification (LLOQ), and a blank plasma after the upper limit of quantification (ULOQ). The lower panel was a chromatogram of the internal standard (PQ-d_6).

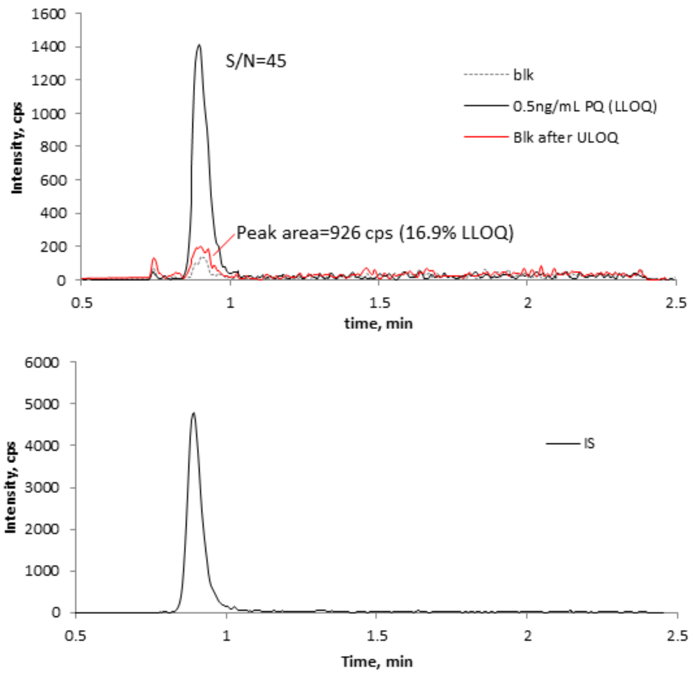

Table 1 Inter-day average back calculated calibrator concentrations

		Cal 1	% ACCU-RACY	Cal 2	% ACCU-RACY	Cal 3	% ACCU-RACY	Cal 4	% ACCU-RACY	Cal 5	% ACCU-RACY	Cal 6	% ACCU-RACY	Cal 7	% ACCU-RACY	slope	y-intercept	R
	Conc ng/ml	**0.500**		**1.00**		**1.50**		**5.00**		**10.0**		**25.0**		**50.0**				
	Run ID																	
	1	0.451	90	1.02	102.0	1.43	95.3	5.22	104	10.8	108.0	26.0	104	48.1	96	0.4270	0.0649	0.9988
	2	0.564	112.8	0.941	94.1	1.30	87	5.41	108	9.88	98.8	24.8	99	50.1	100	0.4670	0.0506	0.9995
	3	0.434	86.8	0.992	99	1.41	94.0	5.47	109	11.1	111.0	26.2	104.8	47.4	95	0.5010	0.0796	0.9977
**Theoretical**																		
**conc.**		**0.500**		**1.00**		**1.50**		**5.00**		**10.0**		**25.0**		**50.0**				
**Mean**		0.48		0.98		1.4		5.4		10.6		26		49		0.4650	0.0650	0.9987
**SD**		0.1		0.0		0.1		0.1		0.6		0.8		1.4		0.0370	0.0145	0.0009
**%CV**		14.6		4.1		5.1		2.4		6.0		3.0		2.9		7.97		0.09
**% dev**		-3.4		-1.6		-8.0		7.3		5.9		2.7		-2.9				
**n**		3		3		3		3		3		3		3				

Table 2 Inter- and intra-day accuracy and precision

		LLOQ	Low VS	Medium VS	High VS		Intra-assay	LLOQ	Low VS	Medium VS	High VS
Run ID	Sample #	**0.500ng/mL**	**1.50ng/mL**	**20.0ng/mL**	**40.0ng/mL**	**Run ID**	**statistics**	**0.500**	**1.50**	**20.0**	**40.0**
**1**	1	0.445	1.42	19.7	39.6	**1**	Mean	0.51	1.58	20.2	40.1
	2	0.441	1.56	20.3	41.3		SD	0.06	0.09	0.69	1.40
	3	0.528	1.65	21.0	41.2		%CV	11.1	5.46	3.40	3.50
	4	0.523	1.63	20.2	40.9		%dev	2.30	5.6	0.8	0.21
	5	0.582	1.60	20.7	39.9		n	6	6	6	6
	6	0.550	1.64	19.1	37.6						
2	1	0.560	1.39	19.6	40.4	**2**	Mean	0.55	1.52	18.9	40.1
	2	0.597	1.54	18.4	42.4		SD	0.04	0.10	1.07	1.64
	3	0.539	1.48	17.6	39.6		%CV	7.44	6.85	5.67	4.08
	4	0.560	1.54	18.7	41.5		%dev	10.0	1.3	-5.7	0.33
	5	0.476	1.70	20.6	38.0		n	6	6	6	6
	6	0.569	1.47	18.3	38.9						
**3**	1	0.570	1.75	20.0	41.4	**3**	Mean	0.54	1.68	20.3	42.9
	2	0.522	1.69	20.3	41.2		SD	0.02	0.04	0.60	1.52
	3	0.557	1.66	20.0	44.4		%CV	3.98	2.11	2.93	3.53
	4	0.539	1.68	19.9	44.9		%dev	7.93	12.2	1.67	7.25
	5	0.512	1.65	20.3	42.9		n	6	6	6	6
	6	0.538	1.67	21.5	42.6						
**Interassay Statistics**						**Intraassay Ranges**					
		**0.500**	**1.50**	**20.0**	**40.0**			**0.500**	**1.50**	**20.0**	**40.0**
	mean	0.5	2	20	41		Mean	0.51-0.55	1.52-1.68	18.9-20.3	40.1-42.9
	SD	0.0	0.1	1	2		SD	0.02-0.06	0.04-0.10	0.60-1.07	1.40-1.64
	%CV	8.1	6.4	5.1	4.8		%CV	3.98-11.1	2.11-6.85	2.93-5.67	3.50-4.08
	%dev	6.8	6.4	-1.1	2.6		%dev	2.30-10.0	1.3-12.2	(-5.7)-1.67	0.21-7.25
	n	18	18	18	18		n	6	6	6	6

Table 3A. Precision of Peak Areas and Peak Area Ratio in Set 1-3

	Precision (%CV, n=7)								
	Piperaquine Peak Area			Internal Standard Peak Area			Peak area ratio		
Conc (ng/ml)	Set 1b	Set 2	Set3	Set 1b	Set 2	Set3	set1b	set2	set3
Low VS (1.5)	2.8	2.7	2.2	2.8	2.9	2.5	1.7	2.0	3.3
Med VS (20)	4.6	1.3	4.2	8.8	5.2	1.8	4.9	5.0	4.6
High VS (40)	4.0	2.4	4.0	3.2	2.2	1.9	1.9	2.8	2.8
Column	A	B	C	D	E	F	G	H	I

Conc (ng/ml)	B-A	E-D	H-G	I-H
Low VS (1.5)	-0.1	0.2	0.3	1.2
Med VS (20)	-3.3	-3.7	0.1	-0.5
High VS (40)	-1.7	-1.0	0.9	0.0
	<5%	<5%	<5%	<5%

Table 3B, matrix effect(ME), recovery(RE) and process efficiency (PE) of PQ

	Mean Peak Area (n=7)						Matrix Effect		Recovery		PE	
	PQ Peak Area			IS Peak Area								
Conc (ng/ml)	Set 1b	Set 2	Set3	Set 1	Set 2	Set3	PQ	IS	PQ	IS	PQ	IS
Low VS (1.5)	11400	13929	14929	19729	23986	21814	122	122	107.2	90.9	131.0	110.6
Med VS (20)	194857	234286	196000	21914	24629	22357	120	112	83.7	90.8	100.6	102.0
High VS (40)	414286	485714	400429	21386	24357	22571	117	114	82.4	92.7	96.7	105.5
	A	B	C	D	E	F	G	H	I	J	K	L

Conc (ng/ml)	H-G
Low VS (1.5)	-0.6
Med VS (20)	-7.8
High VS (40)	-3.3

Table 3C Slopes of PQ standard lines for set 1, set 2, and set 3.

	slope		
lot#	set1	set2	set3
1	0.502	0.481	0.427
2	0.489	0.528	0.446
3	0.502	0.499	0.435
4	0.474	0.498	0.436
5	0.481	0.511	0.467
6	0.489	0.510	0.443
7	0.486	0.496	0.448
mean	0.489	0.50329	0.44314
SD	0.01030	0.01478	0.01277
%CV	2.11	2.94	2.88
	A	B	C

Table 3 D. Peak Area Data in Sets 1-3 (run date June 2, 2015)

		Piperaquine peak area				IS peak area				Peak area ratio		
Conc. (ng/ml)	Matrix Lot #	set1a	set1b	set2	set3	set1a	set1b	set2	set3	set1b	set2	set3
Low (1.5)	1	1.08E+04	1.19E+04	1.45E+04	1.50E+04	1.78E+04	2.03E+04	2.44E+04	2.20E+04	0.586	0.594	0.682
	2	1.20E+04	1.14E+04	1.43E+04	1.48E+04	1.81E+04	2.00E+04	2.52E+04	2.14E+04	0.570	0.567	0.692
	3	1.21E+04	1.17E+04	1.35E+04	1.46E+04	1.80E+04	2.04E+04	2.40E+04	2.26E+04	0.574	0.563	0.646
	4	1.18E+04	1.13E+04	1.38E+04	1.45E+04	1.80E+04	1.98E+04	2.34E+04	2.14E+04	0.571	0.590	0.678
	5	1.15E+04	1.13E+04	1.37E+04	1.49E+04	1.65E+04	1.94E+04	2.36E+04	2.14E+04	0.582	0.581	0.696
	6	1.12E+04	1.09E+04	1.36E+04	1.53E+04	1.70E+04	1.92E+04	2.31E+04	2.25E+04	0.568	0.589	0.680
	7	1.08E+04	1.13E+04	1.41E+04	1.54E+04	1.73E+04	1.90E+04	2.42E+04	2.14E+04	0.595	0.583	0.720
	mean	11457	11400	13929	14929	17529	19729	23986	21814	0.58	0.58	0.68
	STD	541	321	377	335	610	544	703	549	0.01	0.01	0.02
	CV%	4.7	2.8	2.7	2.2	3.5	2.8	2.9	2.5	1.7	2.0	3.3
Med (20)	1	2.11E+05	2.04E+05	2.33E+05	1.84E+05		2.55E+04	2.37E+04	2.24E+04	8.00	9.83	8.21
	2	2.09E+05	2.07E+05	2.35E+05	1.96E+05		2.31E+04	2.42E+04	2.30E+04	8.96	9.71	8.52
	3	2.04E+05	2.02E+05	2.32E+05	1.92E+05		2.24E+04	2.74E+04	2.18E+04	9.02	8.47	8.81
	4	1.97E+05	1.89E+05	2.36E+05	1.91E+05		2.09E+04	2.47E+04	2.25E+04	9.04	9.55	8.49
	5	2.00E+05	1.89E+05	2.29E+05	2.05E+05		2.13E+04	2.38E+04	2.26E+04	8.87	9.62	9.07
	6	1.99E+05	1.86E+05	2.37E+05	2.08E+05		2.02E+04	2.42E+04	2.21E+04	9.21	9.79	9.41
	7	1.93E+05	1.87E+05	2.38E+05	1.96E+05		2.00E+04	2.44E+04	2.21E+04	9.35	9.75	8.87
	mean	201857	194857	234286	196000		21914	24629	22357	8.9	9.5	8.8
	STD	6492	9045	3147	8266		1937	1268	395	0.44	0.48	0.40
	CV%	3.2	4.6	1.3	4.2		8.8	5.2	1.8	4.9	5.0	4.6
High(40.0)	1	4.29E+05	4.33E+05	4.70E+05	3.80E+05		2.18E+04	2.46E+04	2.22E+04	20	19	17
	2	4.28E+05	4.34E+05	4.99E+05	4.14E+05		2.24E+04	2.39E+04	2.32E+04	19	21	18
	3	4.22E+05	4.27E+05	4.80E+05	3.93E+05		2.16E+04	2.43E+04	2.26E+04	20	20	17
	4	4.19E+05	4.08E+05	4.80E+05	3.82E+05		2.17E+04	2.43E+04	2.19E+04	19	20	17
	5	4.09E+05	4.01E+05	4.78E+05	4.22E+05		2.10E+04	2.36E+04	2.26E+04	19	20	19
		4.00E+05	4.03E+05	4.95E+05	4.06E+05		2.08E+04	2.45E+04	2.29E+04	19	20	18
	6	4.01E+05	3.94E+05	4.98E+05	4.06E+05		2.04E+04	2.53E+04	2.26E+04	19	20	18
	mean	415429	414286	485714	400429		21386	24357	22571	19	20	18
	STD	12150	16610	11441	15935.88		684	541	427	0.37	0.56	0.51
	CV%	2.9	4.0	2.4	4.0		3.2	2.2	1.9	1.9	2.8	2.8

Table 4 Effect of carryover on quantification of LLOQ

	Control (calibrator #1)	After ULOQ
Nominal conc., ng/mL	0.5	0.5
1	0.465	0.490
2	0.496	0.567
3	0.503	0.534
mean	0.488	0.530
SD	0.020	0.039
CV	4.14	7.28
Accuracy, %	-2.4	6.1
%difference		8.7
