# Supplementary material for: Determination of piperaquine concentration in human plasma and the correlation of capillary versus venous plasma concentrations
Source: PLoS One. 2020 May 29;15(5):e0233893. doi: 10.1371/journal.pone.0233893 (PMC7259774; doi:10.1371/journal.pone.0233893)
Supplement: S3 File — (PDF) [file pone.0233893.s007.pdf]

Polarity/Scan Type: N/A **API2000 S/N: H0079805 Printing Time: 4:40:10 PM
*Post PM Printing Date: Friday, March 13, 2020

	Sample Name	Sa mp le T	File Name	Analyte Peak Area (counts)	Analyte Peak Height (cp	Anal yte Conc	Ana lyte Ret	IS Peak Area (counts)	IS Peak Height (cps)	U s e	R e c	Calc ulate d Co	Accu racy (%)
3	Blk2	Blan	60209PQVald	9.86e+003	9.95e+002	0.00	1.44	9.37e+006	3.14e+005		☑	N/A	N/A
4	STD1	Sta	60209PQVald	7.58e+004	7.22e+003	20.0	1.36	5.71e+006	2.01e+005	☑	☐	20.9	105.
5	STD2	Sta	60209PQVald	1.81e+005	1.78e+004	50.0	1.35	5.92e+006	2.03e+005	☑	☐	44.3	88.5
6	STD3	Sta	60209PQVald	3.94e+005	3.92e+004	100.	1.34	6.30e+006	2.19e+005	☑	☑	87.2	87.2
7	STD4	Sta	60209PQVald	8.69e+005	8.39e+004	200.	1.34	5.41e+006	2.02e+005	☑	☐	219.	110.
8	STD5	Sta	60209PQVald	2.19e+006	2.19e+005	500.	1.33	5.75e+006	2.10e+005	☑	☐	516.	103.
9	STD6	Sta	60209PQVald	3.28e+006	2.99e+005	750.	1.32	5.68e+006	2.04e+005	☑	☐	780.	104.
10	STD7	Sta	60209PQVald	4.05e+006	3.47e+005	1000.	1.31	5.87e+006	2.03e+005	☑	☐	930.	93.0
11	D blk	Dou	60209PQVald	1.02e+004	5.54e+002	0.00	1.44	1.58e+003	1.89e+002		☑	N/A	N/A
12	LOQ1	Qua	60209PQVald	7.74e+004	7.43e+003	20.0	1.36	5.82e+006	2.09e+005	☑	☐	20.9	105.
13	LOQ2	Qua	60209PQVald	7.94e+004	7.26e+003	20.0	1.36	5.82e+006	2.02e+005	☑	☐	21.4	107.
14	LOQ3	Qua	60209PQVald	7.97e+004	7.46e+003	20.0	1.35	5.56e+006	1.92e+005	☑	☐	22.3	112.
15	LOQ4	Qua	60209PQVald	7.92e+004	7.32e+003	20.0	1.36	5.59e+006	1.94e+005	☑	☐	22.1	111.
16	LOQ5	Qua	60209PQVald	7.47e+004	7.25e+003	20.0	1.35	5.26e+006	1.91e+005	☑	☐	22.2	111.
17	L1	Qua	60209PQVald	1.13e+005	1.05e+004	30.0	1.35	5.48e+006	1.96e+005	☑	☐	30.7	102.
18	L2	Qua	60209PQVald	1.10e+005	1.11e+004	30.0	1.36	5.55e+006	1.99e+005	☑	☐	29.8	99.4
19	L3	Qua	60209PQVald	1.10e+005	1.07e+004	30.0	1.36	5.61e+006	1.94e+005	☑	☐	29.5	98.4
20	L4	Qua	60209PQVald	1.13e+005	1.08e+004	30.0	1.35	5.69e+006	1.96e+005	☑	☐	29.7	99.1
21	L5	Qua	60209PQVald	1.11e+005	1.10e+004	30.0	1.35	5.58e+006	1.91e+005	☑	☐	29.8	99.4
22	M1	Qua	60209PQVald	1.23e+006	1.21e+005	300.	1.32	5.74e+006	1.95e+005	☑	☐	291.	97.0
23	M2	Qua	60209PQVald	1.23e+006	1.28e+005	300.	1.32	5.79e+006	1.98e+005	☑	☐	290.	96.5

Sample Name: "STD7" Sample ID: "" File: "60209PQVald2.wiff"
Peak Name: "PQ" Mass(es): "535.250/288.050 Da"
Comment: "" Annotation: ""

```
Sample Index:      18
Sample Type:       Standard
Concentration:     1000.    ng/mL
Calculated Conc:   930.     ng/mL
Acq. Date:         6/2/2009
Acq. Time:         1:19:44 PM

Modified:          No
Proc. Algorithm:   Analyst Classic
Bunching Factor:   4
Noise Threshold:   3.60     cps
Area Threshold:    18.01    cps
,Num. Smooths:     4
Sep. Width:        0.20
Sep. Height:       0.01
Exp. Peak Ratio:   5.00
Exp. Adj. Ratio:   4.00
Exp. Val. Ratio:   3.00
RT Window:         30.0     sec
Expected RT:       1.18     min
Use Relative RT:   No

Int. Type:         Base To Base
Retention Time:    1.31     min
Area:              4.05e+006  counts
Height:            3.47e+005  cps
Start Time:        1.16     min
End Time:          2.81     min
```

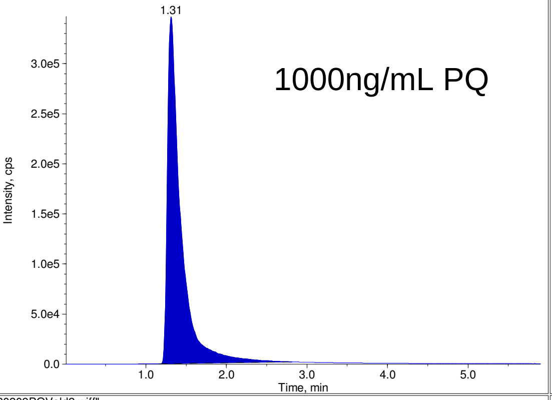

Sample Name: "STD7" Sample ID: "" File: "60209PQVald2.wiff"
Peak Name: "PQ-IS(IS)" Mass(es): "591.200/204.800 Da"
Comment: "" Annotation: ""

```
Sample Index:      18
Sample Type:       Standard
Concentration:     1.00     ng/mL
Calculated Conc:   N/A
Acq. Date:         6/2/2009
Acq. Time:         1:19:44 PM

Modified:          No
Proc. Algorithm:   Analyst Classic
Bunching Factor:   4
Noise Threshold:   5.22     cps
Area Threshold:    26.08    cps
,Num. Smooths:     4
Sep. Width:        0.20
Sep. Height:       0.01
Exp. Peak Ratio:   5.00
Exp. Adj. Ratio:   4.00
Exp. Val. Ratio:   3.00
RT Window:         30.0     sec
Expected RT:       4.27     min
Use Relative RT:   No

Int. Type:         Base To Base
Retention Time:    4.39     min
Area:              5.87e+006  counts
Height:            2.03e+005  cps
Start Time:        4.05     min
End Time:          5.90     min
```

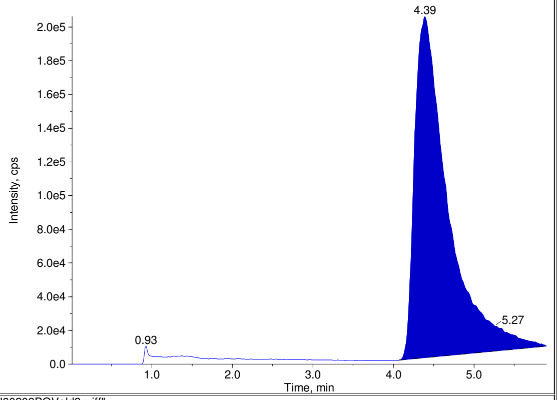

Sample Name: "D blk" Sample ID: "" File: "60209PQVald2.wiff"
Peak Name: "PQ" Mass(es): "535.250/288.050 Da"
Comment: "" Annotation: ""

```
Sample Index:      19
Sample Type:       Double Blank
Concentration:     0.00     ng/mL
Calculated Conc:   N/A
Acq. Date:         6/2/2009
Acq. Time:         1:26:56 PM

Modified:          Yes
Proc. Algorithm:   Analyst Classic
Bunching Factor:   4
Noise Threshold:   8.44     cps
Area Threshold:    42.19    cps
,Num. Smooths:     4
Sep. Width:        0.20
Sep. Height:       0.01
Exp. Peak Ratio:   5.00
Exp. Adj. Ratio:   4.00
Exp. Val. Ratio:   3.00
RT Window:         30.0     sec
Expected RT:       1.44     min
Use Relative RT:   No

Int. Type:         Valley
Retention Time:    1.44     min
Area:              1.02e+004  counts
Height:            5.54e+002  cps
Start Time:        1.21     min
End Time:          1.83     min
```

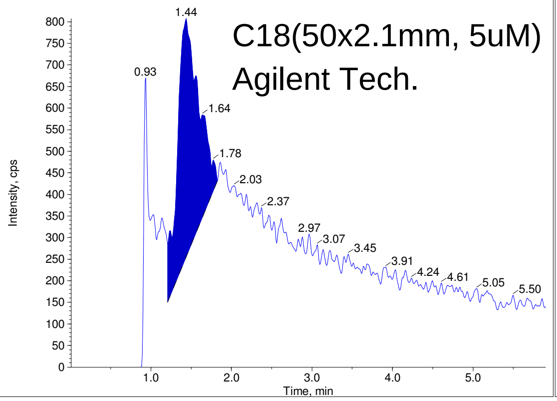

Sample Name: "D blk" Sample ID: "" File: "60209PQVald2.wiff"
Peak Name: "PQ-IS(IS)" Mass(es): "591.200/204.800 Da"
Comment: "" Annotation: ""

```
Sample Index:      19
Sample Type:       Double Blank
Concentration:     1.00     ng/mL
Calculated Conc:   N/A
Acq. Date:         6/2/2009
Acq. Time:         1:26:56 PM

Modified:          Yes
Proc. Algorithm:   Analyst Classic
Bunching Factor:   4
Noise Threshold:   5.22     cps
Area Threshold:    26.08    cps
,Num. Smooths:     4
Sep. Width:        0.20
Sep. Height:       0.01
Exp. Peak Ratio:   5.00
Exp. Adj. Ratio:   4.00
Exp. Val. Ratio:   3.00
RT Window:         30.0     sec
Expected RT:       4.27     min
Use Relative RT:   No

Int. Type:         Base To Base
Retention Time:    4.19     min
Area:              1.58e+003  counts
Height:            1.89e+002  cps
Start Time:        4.14     min
End Time:          4.41     min
```

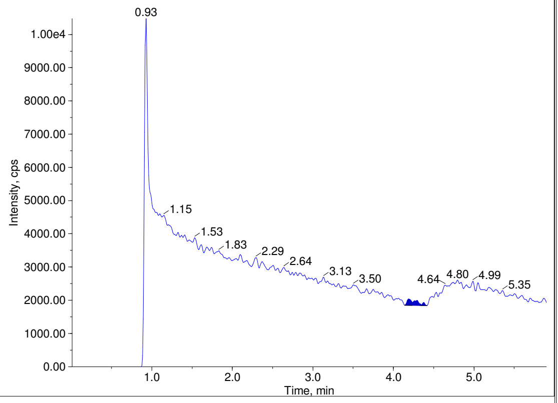

Mobile phase solvent: 2.5mM NH_4HCO_3-MeOH (1:4, v/v); ref: Lee et al. Xenobotica, 2012, 42(11):1088-95.

	Sample Name	Sampl e T	Acquisition Date	Vial Po	File Name	Analyte Peak	Analyte Peak Area (counts)	Analyte Concentration	IS Peak Area (counts)	Use	Record	Calculated Concen	Relative Retention Tim	Accuracy (%)
7	blk	Blan	9/13/2013 5:26:57 P	2	2013 913PQvalid2.wiff	PQ1	2.67e+003	0.00	1.39e+004		☐	N/A	1.01	N/A
8	STD0.5ng	Unk	9/13/2013 5:30:21 P	3	2013 913PQvalid2.wiff	PQ1	7.45e+003	N/A	1.44e+004		☐	0.502	1.01	N/A
9	STD1ng	Stan	9/13/2013 5:33:45 P	4	2013 913PQvalid2.wiff	PQ1	1.40e+004	1.00	1.41e+004	☑	☐	1.01	1.01	101.
10	STD1.5ng	Stan	9/13/2013 5:37:08 P	5	2013 913PQvalid2.wiff	PQ1	2.12e+004	1.50	1.47e+004	☑	☐	1.48	1.01	98.5
11	STD5ng	Stan	9/13/2013 5:40:33 P	6	2013 913PQvalid2.wiff	PQ1	6.74e+004	5.00	1.43e+004	☑	☐	4.91	1.01	98.3
12	STD10ng	Stan	9/13/2013 5:43:57 P	7	2013 913PQvalid2.wiff	PQ1	1.41e+005	10.0	1.44e+004	☑	☐	10.2	1.00	102.
13	STD25ng	Stan	9/13/2013 5:47:21 P	8	2013 913PQvalid2.wiff	PQ1	3.55e+005	25.0	1.43e+004	☑	☐	26.0	1.01	104.
14	STD50ng	Stan	9/13/2013 5:50:45 P	9	2013 913PQvalid2.wiff	PQ1	6.89e+005	50.0	1.46e+004	☑	☐	49.6	1.01	99.2
15	STD100ng	Stan	9/13/2013 5:54:10 P	10	2013 913PQvalid2.wiff	PQ1	1.45e+006	100.	1.45e+004	☑	☐	105.	1.01	105.
16	STD250ng	Stan	9/13/2013 5:57:34 P	11	2013 913PQvalid2.wiff	PQ1	3.60e+006	250.	1.52e+004	☑	☐	250.	1.01	99.9
17	Dblk	Unk	9/13/2013 6:00:57 P	1	2013 913PQvalid2.wiff	PQ1	2.81e+003	N/A	8.81e+001		☐	33.6	0.823	N/A
18	1ng1	Qual	9/13/2013 6:04:18 P	12	2013 913PQvalid2.wiff	PQ1	1.67e+004	1.00	1.43e+004	☑	☐	1.18	1.01	118.
19	1ng2	Qual	9/13/2013 6:07:42 P	13	2013 913PQvalid2.wiff	PQ1	1.34e+004	1.00	1.44e+004	☑	☐	0.938	1.00	93.8
20	1ng3	Qual	9/13/2013 6:11:06 P	14	2013 913PQvalid2.wiff	PQ1	1.45e+004	1.00	1.47e+004	☑	☐	0.997	1.01	99.7
21	1ng4	Qual	9/13/2013 6:14:30 P	15	2013 913PQvalid2.wiff	PQ1	1.40e+004	1.00	1.40e+004	☑	☐	1.01	1.00	101.

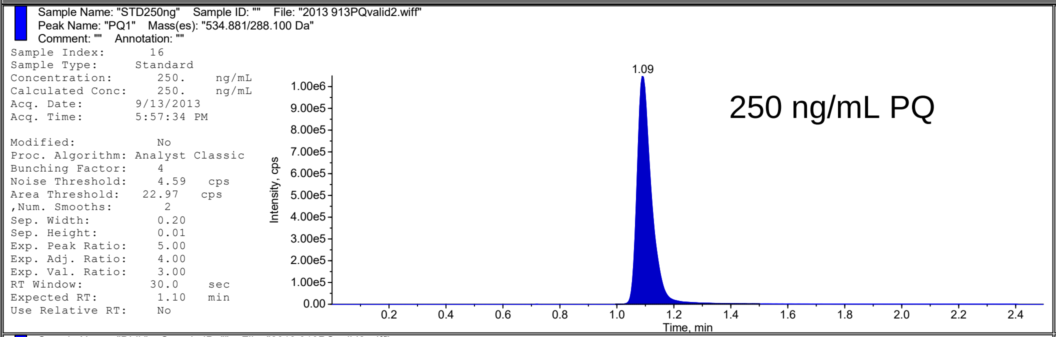

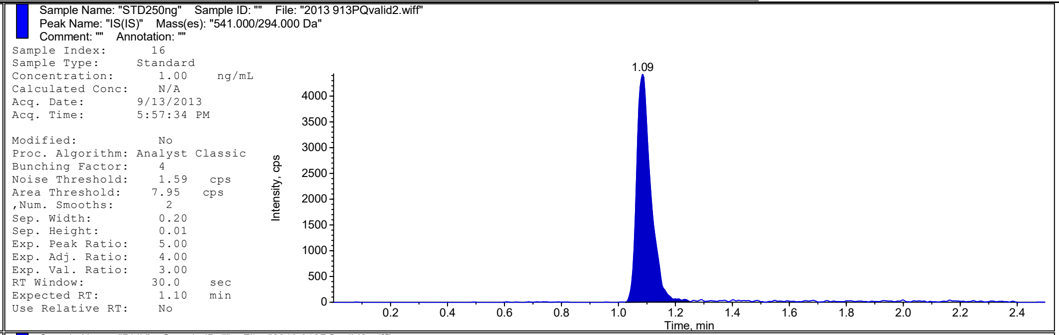

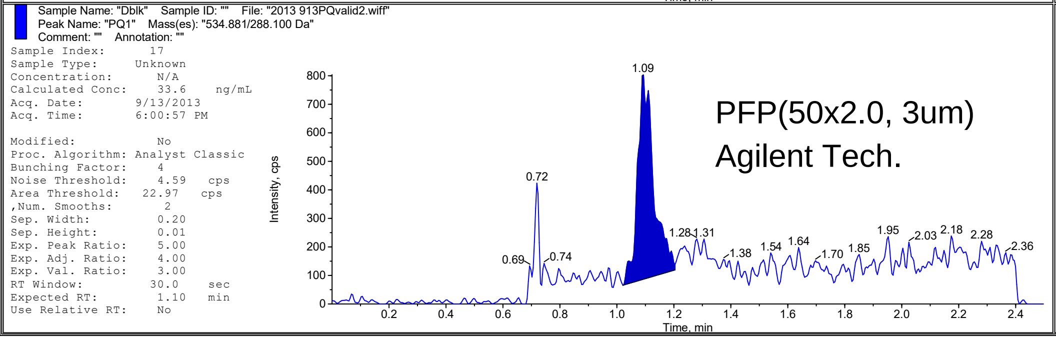

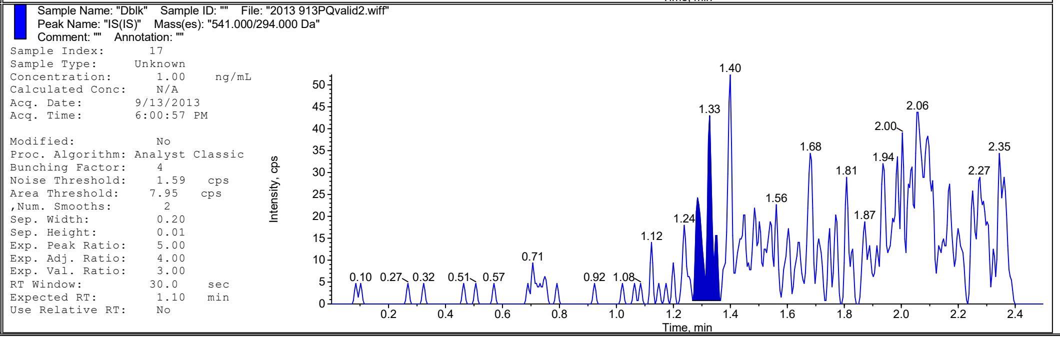

Collected by: N/A
Electronic Signature: no
Operator: Drug Research Unit

MPA: 20mMNH4FA 0.14%TFA,MPB: 0.1%TFA in MeCN; Ref: Kjellin et al. Bioanalysis, 2014, 6(23): 3081-89.

	Sample Name	Sample T	Acquisition Date	Vial Po	File Name	Analyte Peak	Analyte Peak Area (counts)	Analyte Concentration	IS Peak Area (counts)	Use	Record	Calculated Concen	Relative Retention Tim	Accuracy (%)
26	STD2	Stan	5/29/2015 11:51:28 A	9	2015 5_29newrang	PQ1	8.66e+003	1.00	1.50e+004	☑	☐	0.992	1.01	99.2
27	STD3	Stan	5/29/2015 11:55:04 A	10	2015 5_29newrang	PQ1	1.30e+004	1.50	1.65e+004	☑	☐	1.41	1.00	93.9
28	STD4	Stan	5/29/2015 11:58:47 A	11	2015 5_29newrang	PQ1	4.66e+004	5.00	1.65e+004	☑	☐	5.47	0.996	109.
29	STD5	Stan	5/29/2015 12:02:26 P	12	2015 5_29newrang	PQ1	9.23e+004	10.0	1.63e+004	☑	☐	11.1	1.01	111.
30	STD6	Stan	5/29/2015 12:06:06 P	13	2015 5_29newrang	PQ1	2.25e+005	25.0	1.71e+004	☑	☐	26.2	1.01	105.
31	STD7	Stan	5/29/2015 12:09:44 P	14	2015 5_29newrang	PQ1	4.33e+005	50.0	1.82e+004	☑	☐	47.4	1.01	94.8
32	Dblk1	Dou	5/29/2015 12:13:19 P	6	2015 5_29newrang	PQ1	9.75e+002	0.00	7.98e+001		☐	N/A	1.01	N/A
33	LOQ1	Qual	5/29/2015 12:16:59 P	15	2015 5_29newrang	PQ1	6.05e+003	0.500	1.66e+004	☑	☐	0.570	1.01	114.
34	LOQ2	Qual	5/29/2015 12:20:38 P	16	2015 5_29newrang	PQ1	5.64e+003	0.500	1.65e+004	☑	☐	0.522	1.01	104.

Sample Name: "STD7" Sample ID: "" File: "2015 5_29newrangeValid3_VC.wiff"
Peak Name: "PQ1" Mass(es): "534.881/288.100 Da"
Comment: "" Annotation: ""

```
Sample Index:       31
Sample Type:       Standard
Concentration:       50.0    ng/mL
Calculated Conc:     47.4    ng/mL
Acq. Date:         5/29/2015
Acq. Time:         12:09:44 PM

Modified:            No
Proc. Algorithm: Analyst Classic
Bunching Factor:     6
Noise Threshold:     5.40   cps
Area Threshold:    26.98   cps
,Num. Smooths:        2
Sep. Width:          0.20
Sep. Height:         0.01
Exp. Peak Ratio:     5.00
Exp. Adj. Ratio:     4.00
Exp. Val. Ratio:     3.00
RT Window:          30.0    sec
Expected RT:        0.940   min
Use Relative RT:     No

Int. Type:         Base To Base
Retention Time:     0.942   min
Area:          4.33e+005   counts
Height:          1.13e+005  cps
Start Time:         0.859   min
End Time:            1.16   min
```

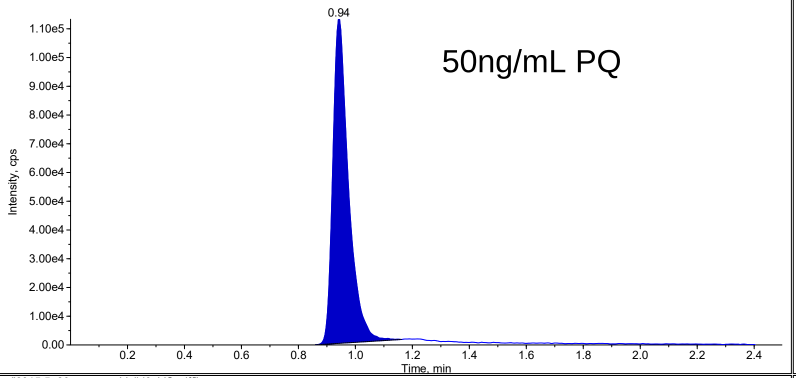

Sample Name: "STD7" Sample ID: "" File: "2015 5_29newrangeValid3_VC.wiff"
Peak Name: "IS(IS)" Mass(es): "541.000/294.000 Da"
Comment: "" Annotation: ""

```
Sample Index:       31
Sample Type:       Standard
Concentration:       1.00    ng/mL
Calculated Conc:     N/A
Acq. Date:         5/29/2015
Acq. Time:         12:09:44 PM

Modified:            No
Proc. Algorithm: Analyst Classic
Bunching Factor:     6
Noise Threshold:     5.39   cps
Area Threshold:    26.94   cps
,Num. Smooths:        2
Sep. Width:          0.20
Sep. Height:         0.01
Exp. Peak Ratio:     5.00
Exp. Adj. Ratio:     4.00
Exp. Val. Ratio:     3.00
RT Window:          30.0    sec
Expected RT:        0.910   min
Use Relative RT:     No

Int. Type:         Base To Base
Retention Time:     0.938   min
Area:          1.82e+004   counts
Height:          4.91e+003  cps
Start Time:         0.855   min
End Time:            1.20   min
```

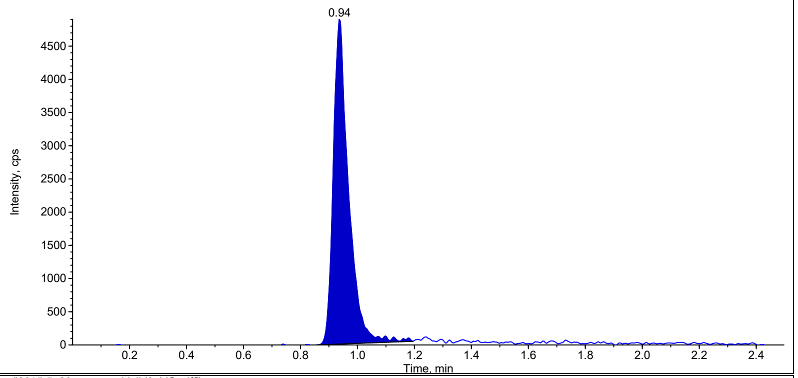

Sample Name: "Dblk1" Sample ID: "" File: "2015 5_29newrangeValid3_VC.wiff"
Peak Name: "PQ1" Mass(es): "534.881/288.100 Da"
Comment: "" Annotation: ""

```
Sample Index:       32
Sample Type:       Double Blank
Concentration:       0.00    ng/mL
Calculated Conc:     N/A
Acq. Date:         5/29/2015
Acq. Time:         12:13:19 PM

Modified:            No
Proc. Algorithm: Analyst Classic
Bunching Factor:     6
Noise Threshold:     5.40   cps
Area Threshold:    26.98   cps
,Num. Smooths:        2
Sep. Width:          0.20
Sep. Height:         0.01
Exp. Peak Ratio:     5.00
Exp. Adj. Ratio:     4.00
Exp. Val. Ratio:     3.00
RT Window:          30.0    sec
Expected RT:        0.940   min
Use Relative RT:     No

Int. Type:         Base To Base
Retention Time:     0.948   min
Area:          9.75e+002   counts
Height:          2.40e+002  cps
Start Time:         0.838   min
End Time:            1.07   min
```

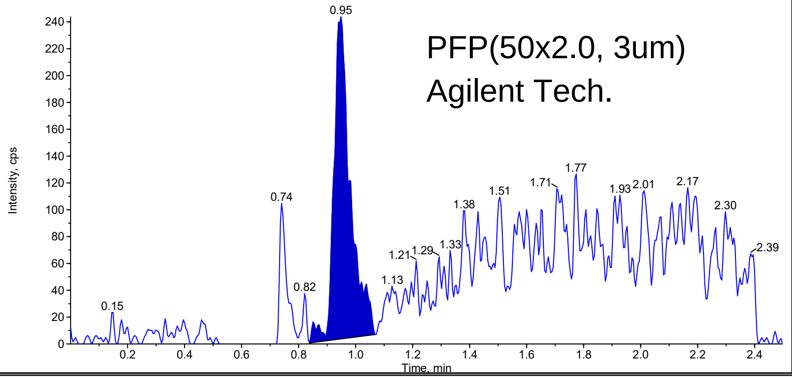

Sample Name: "Dblk1" Sample ID: "" File: "2015 5_29newrangeValid3_VC.wiff"
Peak Name: "IS(IS)" Mass(es): "541.000/294.000 Da"
Comment: "" Annotation: ""

```
Sample Index:       32
Sample Type:       Double Blank
Concentration:       1.00    ng/mL
Calculated Conc:     N/A
Acq. Date:         5/29/2015
Acq. Time:         12:13:19 PM

Modified:            No
Proc. Algorithm: Analyst Classic
Bunching Factor:     6
Noise Threshold:     5.39   cps
Area Threshold:    26.94   cps
,Num. Smooths:        2
Sep. Width:          0.20
Sep. Height:         0.01
Exp. Peak Ratio:     5.00
Exp. Adj. Ratio:     4.00
Exp. Val. Ratio:     3.00
RT Window:          30.0    sec
Expected RT:        0.910   min
Use Relative RT:     No

Int. Type:         Base To Base
Retention Time:     0.942   min
Area:          7.98e+001   counts
Height:          4.82e+001  cps
Start Time:         0.898   min
End Time:            1.04   min
```

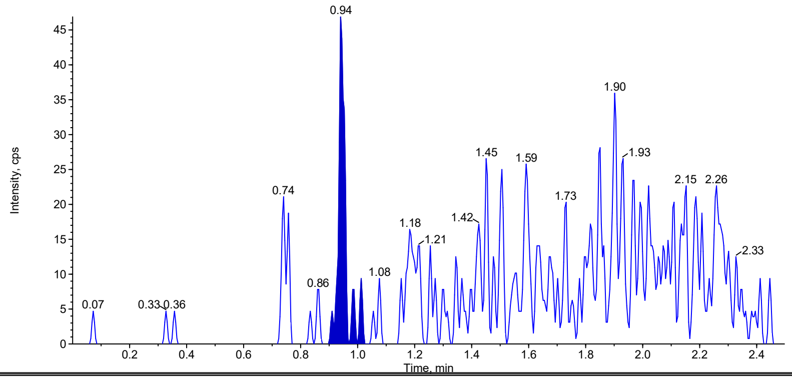

Collected by: N/A
Electronic Signature: no
Operator: Drug Research Unit

MPA: 20mMNH4FA 0.14%TFA,MPB: 0.1%TFA in MeCN; Ref: Kjellin et al. Bioanalysis, 2014, 6(23): 3081-89.
