# Supplementary material for: Determination of piperaquine concentration in human plasma and the correlation of capillary versus venous plasma concentrations
Source: PLoS One. 2020 May 29;15(5):e0233893. doi: 10.1371/journal.pone.0233893 (PMC7259774; doi:10.1371/journal.pone.0233893)

	Sample Name	Sa mp le T	Acquisition Date	Vial Pos itio	File Name	Analyte Peak Area (counts)	Analyt e Conce	IS Peak Area (counts)	U se R	Rec ord Mo	Calcul ated Conce	Relativ e Retenti
19	STD1	Sta	4/8/2015 3:43:37 PM	13	2015 4_07PQvalid3_V	1.29e+004	10.0	3.73e+00	☑	☐	9.94	1.04
20	STD2	Sta	4/8/2015 3:47:12 PM	14	2015 4_07PQvalid3_V	2.63e+004	25.0	3.45e+00	☑	☐	24.9	1.05
21	STD3	Sta	4/8/2015 3:50:47 PM	15	2015 4_07PQvalid3_V	5.06e+004	50.0	3.48e+00	☑	☐	49.8	1.04
22	STD4	Sta	4/8/2015 3:54:22 PM	16	2015 4_07PQvalid3_V	9.82e+004	100.	3.56e+00	☑	☐	96.5	1.05
23	STD5	Sta	4/8/2015 3:57:57 PM	17	2015 4_07PQvalid3_V	2.54e+005	250.	3.46e+00	☑	☐	261.	1.04
24	STD6	Sta	4/8/2015 4:01:37 PM	18	2015 4_07PQvalid3_V	5.35e+005	500.	3.72e+00	☑	☐	513.	1.04
25	STD7	Sta	4/8/2015 4:05:17 PM	19	2015 4_07PQvalid3_V	9.77e+005	1000.	3.57e+00	☑	☐	980.	1.04
26	Dblk	Unk	4/8/2015 4:08:57 PM	11	2015 4_07PQvalid3_V	5.26e+003	N/A	0.00e+00		☐	#DIV/0	0.00
27	LOQ1	Qua	4/8/2015 4:12:32 PM	20	2015 4_07PQvalid3_V	1.50e+004	10.0	3.81e+00	☑	☐	11.7	1.04
28	LOQ2	Qua	4/8/2015 4:16:12 PM	21	2015 4_07PQvalid3_V	1.38e+004	10.0	3.71e+00	☑	☐	10.9	1.04
29	LOQ3	Qua	4/8/2015 4:19:48 PM	22	2015 4_07PQvalid3_V	1.30e+004	10.0	3.51e+00	☑	☐	10.8	1.05
30	LOQ4	Qua	4/8/2015 4:23:23 PM	23	2015 4_07PQvalid3_V	1.32e+004	10.0	3.87e+00	☑	☐	9.76	1.04
31	LOQ5	Qua	4/8/2015 4:26:58 PM	24	2015 4_07PQvalid3_V	1.24e+004	10.0	3.77e+00	☑	☐	9.33	1.04
32	LOQ6	Qua	4/8/2015 4:30:33 PM	25	2015 4_07PQvalid3_V	1.28e+004	10.0	3.65e+00	☑	☐	10.2	1.05
33	L1	Qua	4/8/2015 4:34:08 PM	26	2015 4_07PQvalid3_V	3.52e+004	30.0	3.82e+00	☑	☐	30.6	1.05

Sample Name: "STD7" Sample ID: "" File: "2015 4_07PQvalid3_VC_LH.wiff"
Peak Name: "PQ1" Mass(es): "535.000/288.000 Da"
Comment: "" Annotation: ""

```
Sample Index:       31
Sample Type:      Standard
Concentration:      1000.    ng/mL
Calculated Conc:     980.    ng/mL
Acq. Date:        4/8/2015
Acq. Time:        4:05:17 PM

Modified:            No
Proc. Algorithm: Analyst Classic
Bunching Factor:     6
Noise Threshold:    10.07   cps
Area Threshold:    50.33   cps
,Num. Smooths:        2
Sep. Width:          0.20
Sep. Height:         0.01
Exp. Peak Ratio:     5.00
Exp. Adj. Ratio:     4.00
Exp. Val. Ratio:     3.00
RT Window:          30.0    sec
Expected RT:         1.14   min
Use Relative RT:     No

Int. Type:        Base To Base
Retention Time:      1.15   min
Area:           9.77e+005   counts
Height:           1.23e+005  cps
Start Time:         0.666   min
End Time:            1.99   min
```

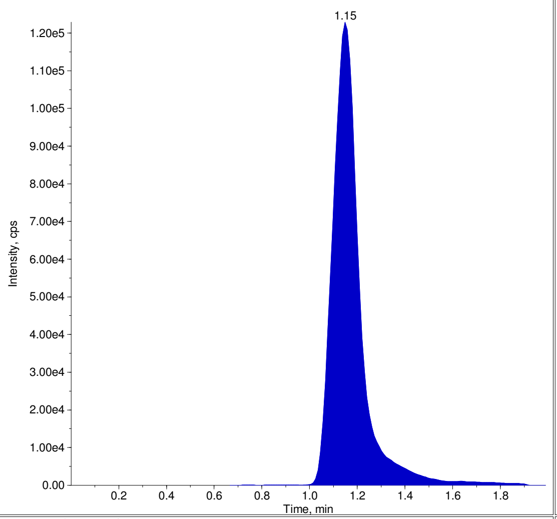

Sample Name: "STD7" Sample ID: "" File: "2015 4_07PQvalid3_VC_LH.wiff"
Peak Name: "PQ_d6(IS)" Mass(es): "541.000/294.000 Da"
Comment: "" Annotation: ""

```
Sample Index:       31
Sample Type:      Standard
Concentration:       1.00    ng/mL
Calculated Conc:     N/A
Acq. Date:        4/8/2015
Acq. Time:        4:05:17 PM

Modified:            No
Proc. Algorithm: Analyst Classic
Bunching Factor:     6
Noise Threshold:     1.68   cps
Area Threshold:     8.40   cps
,Num. Smooths:        2
Sep. Width:          0.20
Sep. Height:         0.01
Exp. Peak Ratio:     5.00
Exp. Adj. Ratio:     4.00
Exp. Val. Ratio:     3.00
RT Window:          30.0    sec
Expected RT:         1.29   min
Use Relative RT:     No

Int. Type:          Valley
Retention Time:      1.11   min
Area:           3.57e+004   counts
Height:           5.24e+003  cps
Start Time:         0.964   min
End Time:            1.60   min
```

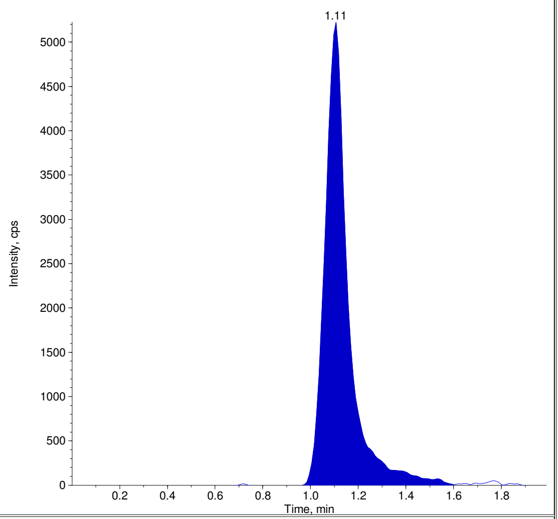

Sample Name: "Dblk" Sample ID: "" File: "2015 4_07PQvalid3_VC_LH.wiff"
Peak Name: "PQ1" Mass(es): "535.000/288.000 Da"
Comment: "" Annotation: ""

```
Sample Index:       32
Sample Type:      Unknown
Concentration:       N/A
Calculated Conc: No Intercept
Acq. Date:        4/8/2015
Acq. Time:        4:08:57 PM

Modified:            No
Proc. Algorithm: Analyst Classic
Bunching Factor:     6
Noise Threshold:    10.07   cps
Area Threshold:    50.33   cps
,Num. Smooths:        2
Sep. Width:          0.20
Sep. Height:         0.01
Exp. Peak Ratio:     5.00
Exp. Adj. Ratio:     4.00
Exp. Val. Ratio:     3.00
RT Window:          30.0    sec
Expected RT:         1.14   min
Use Relative RT:     No

Int. Type:        Base To Base
Retention Time:      1.14   min
Area:           5.26e+003   counts
Height:           4.02e+002  cps
Start Time:         0.984   min
End Time:            1.56   min
```

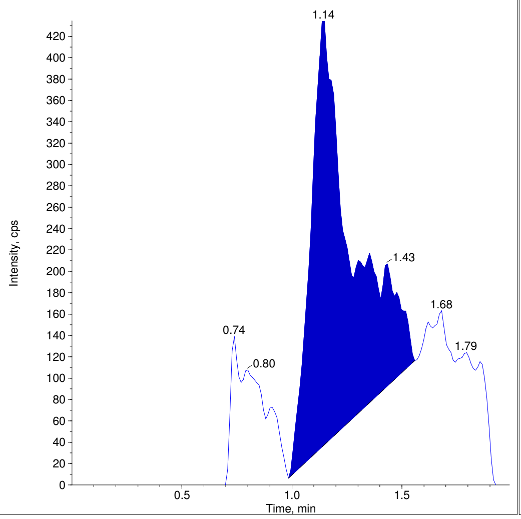

Sample Name: "Dblk" Sample ID: "" File: "2015 4_07PQvalid3_VC_LH.wiff"
Peak Name: "PQ_d6(IS)" Mass(es): "541.000/294.000 Da"
Comment: "" Annotation: ""

```
Sample Index:       32
Sample Type:      Unknown
Concentration:       1.00    ng/mL
Calculated Conc:     N/A
Acq. Date:        4/8/2015
Acq. Time:        4:08:57 PM

Modified:            No
```

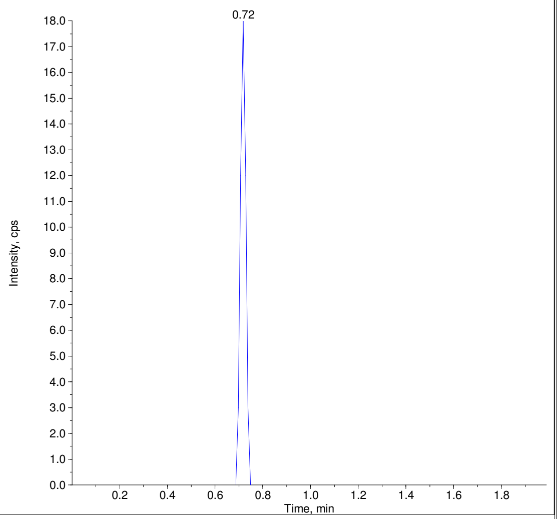

	Sample Name	Sample T	Acquisition Date	Vial Po	File Name	Diluti	Analyte Peak Area (counts)	Analyte Conc	IS Peak Area (counts)	Use	Record	Calculated Co
1	Dblk	Dou	4/14/2015 12:04:59 P	1	2015 4_14crossvalid2_L	1.	1.43e+002	0.00	0.00e+00		☐	N/A
2	Blk	Blan	4/14/2015 12:08:34 P	2	2015 4_14crossvalid2_L	1.	0.00e+000	0.00	2.80e+00		☐	N/A
3	STD1	Sta	4/14/2015 12:12:09 P	3	2015 4_14crossvalid2_L	1.	8.12e+003	10.0	2.51e+00	☑	☐	9.36
4	STD2	Sta	4/14/2015 12:15:44 P	4	2015 4_14crossvalid2_L	1.	1.87e+004	25.0	2.35e+00	☑	☐	25.5
5	STD3	Sta	4/14/2015 12:19:19 P	5	2015 4_14crossvalid2_L	1.	3.61e+004	50.0	2.33e+00	☑	☑	51.2
6	STD4	Sta	4/14/2015 12:22:54 P	6	2015 4_14crossvalid2_L	1.	7.31e+004	100.	2.45e+00	☑	☐	100.
7	STD5	Sta	4/14/2015 12:26:34 P	7	2015 4_14crossvalid2_L	1.	1.71e+005	250.	2.28e+00	☑	☐	255.
8	STD6	Sta	4/14/2015 12:30:14 P	8	2015 4_14crossvalid2_L	1.	3.46e+005	500.	2.35e+00	☑	☐	503.
9	STD7	Sta	4/14/2015 12:33:54 P	9	2015 4_14crossvalid2_L	1.	6.66e+005	1000.	2.29e+00	☑	☐	991.
10	Dblk	Unk	4/14/2015 12:37:34 P	1	2015 4_14crossvalid2_L	1.	2.19e+002	N/A	0.00e+00		☑	#DIV/
11	LOQ1	Qua	4/14/2015 12:41:09 P	10	2015 4_14crossvalid2_L	1.	6.63e+003	10.0	2.23e+00	☑	☐	8.47
12	L1	Qua	4/14/2015 12:44:49 P	11	2015 4_14crossvalid2_L	1.	2.34e+004	30.0	2.34e+00	☑	☐	32.5
13	M1	Qua	4/14/2015 12:48:24 P	12	2015 4_14crossvalid2_L	1.	1.45e+005	200.	2.32e+00	☑	☐	212.
14	H1	Qua	4/14/2015 12:51:59 P	13	2015 4_14crossvalid2_L	1.	5.81e+005	800.	2.40e+00	☑	☐	827.
15	H1_12.5ul	Qua	4/14/2015 12:55:34 P	14	2015 4_14crossvalid2_L	2.	3.16e+005	800.	2.50e+00	☑	☐	859.
16	sample9	Unk	4/14/2015 12:59:09 P	15	2015 4_14crossvalid2_L	1.	6.75e+004	N/A	2.57e+00		☐	88.1

Sample Name: "STD7" Sample ID: "" File: "2015 4_14crossvalid2_LH.wiff"
Peak Name: "PQ1" Mass(es): "535.000/288.000 Da"
Comment: "" Annotation: ""

```
Sample Index:        9
Sample Type:      Standard
Concentration:      1000.    ng/mL
Calculated Conc:     991.    ng/mL
Acq. Date:         4/14/2015
Acq. Time:         12:33:54 PM

Modified:             No
Proc. Algorithm: Analyst Classic
Bunching Factor:      5
Noise Threshold:     3.66   cps
Area Threshold:    18.29   cps
,Num. Smooths:        2
Sep. Width:          0.20
Sep. Height:         0.01
Exp. Peak Ratio:     5.00
Exp. Adj. Ratio:     4.00
Exp. Val. Ratio:     3.00
RT Window:          30.0     sec
Expected RT:         1.31    min
Use Relative RT:     No

Int. Type:        Base To Base
Retention Time:      1.15    min
Area:            6.66e+005   counts
Height:            5.93e+004  cps
Start Time:         0.769    min
End Time:            1.99    min
```

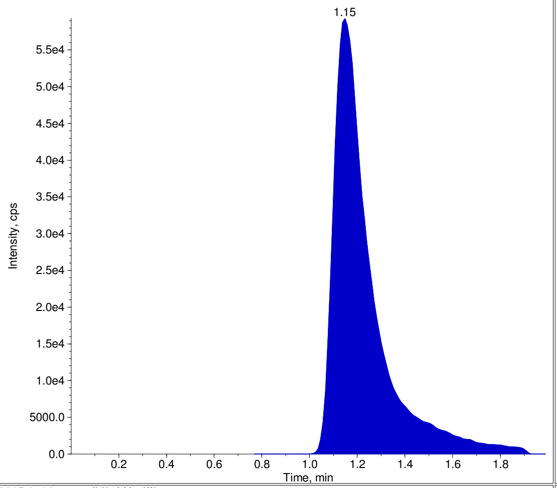

Sample Name: "STD7" Sample ID: "" File: "2015 4_14crossvalid2_LH.wiff"
Peak Name: "PQ_d6(IS)" Mass(es): "541.000/294.000 Da"
Comment: "" Annotation: ""

```
Sample Index:        9
Sample Type:      Standard
Concentration:      1.00     ng/mL
Calculated Conc:     N/A
Acq. Date:         4/14/2015
Acq. Time:         12:33:54 PM

Modified:             No
Proc. Algorithm: Analyst Classic
Bunching Factor:      7
Noise Threshold:     1.68   cps
Area Threshold:     8.40   cps
,Num. Smooths:        2
Sep. Width:          0.20
Sep. Height:         0.01
Exp. Peak Ratio:     5.00
Exp. Adj. Ratio:     4.00
Exp. Val. Ratio:     3.00
RT Window:          30.0     sec
Expected RT:         1.29    min
Use Relative RT:     No

Int. Type:        Base To Base
Retention Time:      1.10    min
Area:            2.29e+004   counts
Height:            1.99e+003  cps
Start Time:         0.923    min
End Time:            1.99    min
```

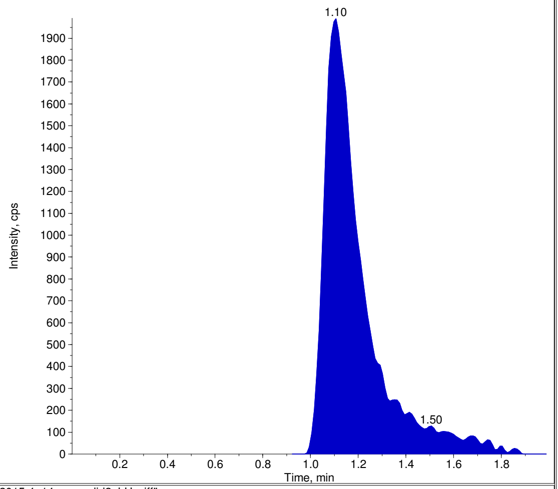

Sample Name: "Dblk" Sample ID: "" File: "2015 4_14crossvalid2_LH.wiff"
Peak Name: "PQ1" Mass(es): "535.000/288.000 Da"
Comment: "" Annotation: ""

```
Sample Index:        10
Sample Type:      Unknown
Concentration:       N/A
Calculated Conc: No Intercept
Acq. Date:         4/14/2015
Acq. Time:         12:37:34 PM

Modified:             Yes
Proc. Algorithm: Analyst Classic
Bunching Factor:      1
Noise Threshold:     3.66   cps
Area Threshold:    18.29   cps
,Num. Smooths:        2
Sep. Width:          0.20
Sep. Height:         0.01
Exp. Peak Ratio:     5.00
Exp. Adj. Ratio:     4.00
Exp. Val. Ratio:     3.00
RT Window:          30.0     sec
Expected RT:         1.23    min
Use Relative RT:     No

Int. Type:          Valley
Retention Time:      1.23    min
Area:            2.19e+002   counts
Height:            4.69e+001  cps
Start Time:          1.20    min
End Time:            1.32    min
```

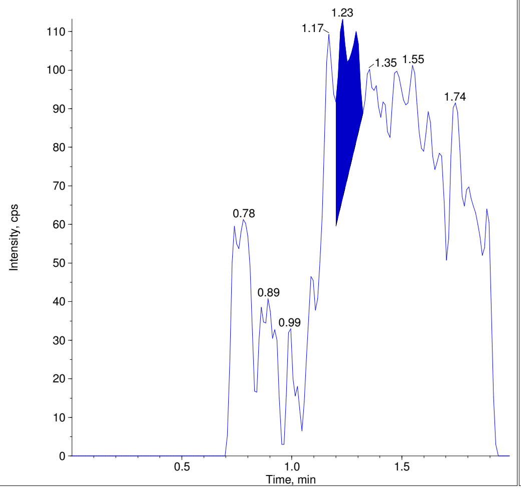

Sample Name: "Dblk" Sample ID: "" File: "2015 4_14crossvalid2_LH.wiff"
Peak Name: "PQ_d6(IS)" Mass(es): "541.000/294.000 Da"
Comment: "" Annotation: ""

```
Sample Index:        10
Sample Type:      Unknown
Concentration:      1.00     ng/mL
Calculated Conc:     N/A
Acq. Date:         4/14/2015
Acq. Time:         12:37:34 PM

Modified:             Yes
```

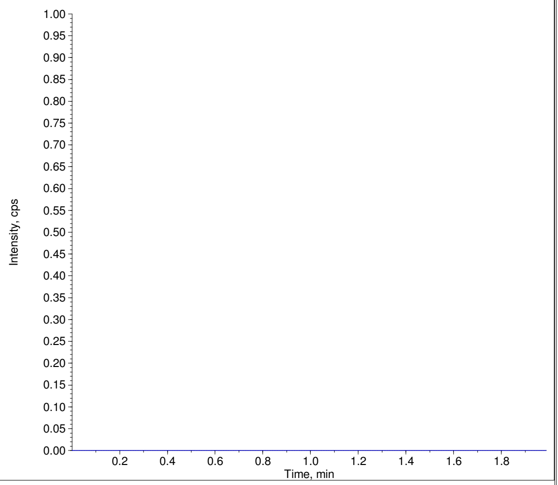

	Sample Name	Sample T	Acquisition Date	File Name
6	BLANK	Blan	6/5/2015 4:03:07 PM	2015 06 05_BC1 PQ SAMPLES_56-65_84-91_315
7	DOUBLE BLANK	Dou	6/5/2015 4:06:42 PM	2015 06 05_BC1 PQ SAMPLES_56-65_84-91_315
8	PQ STD 1	Sta	6/5/2015 4:10:17 PM	2015 06 05_BC1 PQ SAMPLES_56-65_84-91_315
9	PQ STD 2	Sta	6/5/2015 4:13:52 PM	2015 06 05_BC1 PQ SAMPLES_56-65_84-91_315
10	PQ STD 3	Sta	6/5/2015 4:17:28 PM	2015 06 05_BC1 PQ SAMPLES_56-65_84-91_315
11	PQ STD 4	Sta	6/5/2015 4:21:03 PM	2015 06 05_BC1 PQ SAMPLES_56-65_84-91_315
12	PQ STD 5	Sta	6/5/2015 4:24:42 PM	2015 06 05_BC1 PQ SAMPLES_56-65_84-91_315
13	PQ STD 6	Sta	6/5/2015 4:28:22 PM	2015 06 05_BC1 PQ SAMPLES_56-65_84-91_315
14	PQ STD 7	Sta	6/5/2015 4:32:03 PM	2015 06 05_BC1 PQ SAMPLES_56-65_84-91_315
15	DOUBLE BLANK	Dou	6/5/2015 4:35:43 PM	2015 06 05_BC1 PQ SAMPLES_56-65_84-91_315
16	PQ QC LOW 1	Qua	6/5/2015 4:39:18 PM	2015 06 05_BC1 PQ SAMPLES_56-65_84-91_315
17	PQ QC MEDIUM 1	Qua	6/5/2015 4:42:53 PM	2015 06 05_BC1 PQ SAMPLES_56-65_84-91_315
18	PQ QC HIGH 1	Qua	6/5/2015 4:46:28 PM	2015 06 05_BC1 PQ SAMPLES_56-65_84-91_315
19	DOUBLE BLANK	Dou	6/5/2015 4:50:03 PM	2015 06 05_BC1 PQ SAMPLES_56-65_84-91_315
20	56__283__IMWZT__Day 2, predose, pk	Unk	6/5/2015 4:53:38 PM	2015 06 05_BC1 PQ SAMPLES_56-65_84-91_315
21	57__283__I36ZX__Day 2, 30min, pk DP	Unk	6/5/2015 4:57:13 PM	2015 06 05_BC1 PQ SAMPLES_56-65_84-91_315
22	58__283__IJ625__Day 2, 1hr, pk DP	Unk	6/5/2015 5:00:48 PM	2015 06 05_BC1 PQ SAMPLES_56-65_84-91_315

Sample Name: "PQ STD 7" Sample ID: "" File: "2015 06 05_BC1 PQ SAMPLES_56-65_84-91_315_FM_VC.wiff"
Peak Name: "PQ1" Mass(es): "535.000/288.000 Da"
Comment: "" Annotation: ""

```
Sample Index:       43
Sample Type:        Standard
Concentration:      1000.     ng/mL
Calculated Conc:    1010.     ng/mL
Acq. Date:          6/5/2015
Acq. Time:          4:32:03 PM

Modified:              No
Proc. Algorithm: Analyst Classic
Bunching Factor:       6
Noise Threshold: 1.00e-6    cps
Area Threshold: 1.00e-6    cps
,Num. Smooths:          2
Sep. Width:            0.20
Sep. Height:           0.01
Exp. Peak Ratio:       5.00
Exp. Adj. Ratio:       4.00
Exp. Val. Ratio:       3.00
RT Window:            30.0     sec
Expected RT:           1.33    min
Use Relative RT:       No

Int. Type:          Base To Base
Retention Time:        1.33    min
Area:             7.30e+005    counts
Height:             7.12e+004  cps
Start Time:            1.04    min
End Time:              1.99    min
```

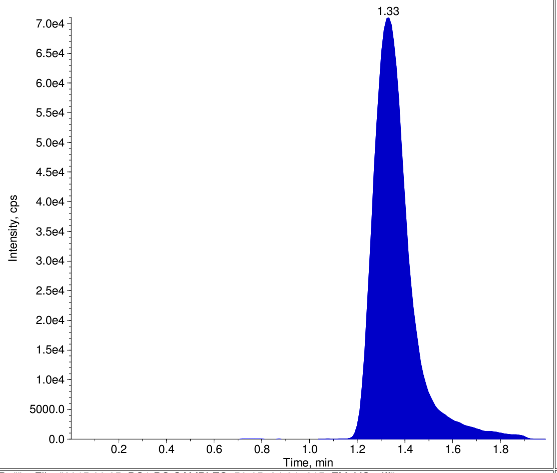

Sample Name: "PQ STD 7" Sample ID: "" File: "2015 06 05_BC1 PQ SAMPLES_56-65_84-91_315_FM_VC.wiff"
Peak Name: "PQ_d6(IS)" Mass(es): "541.000/294.000 Da"
Comment: "" Annotation: ""

```
Sample Index:       43
Sample Type:        Standard
Concentration:      1.00      ng/mL
Calculated Conc:    N/A
Acq. Date:          6/5/2015
Acq. Time:          4:32:03 PM

Modified:              No
Proc. Algorithm: Analyst Classic
Bunching Factor:       6
Noise Threshold: 1.00e-6    cps
Area Threshold: 1.00e-6    cps
,Num. Smooths:          2
Sep. Width:            0.20
Sep. Height:           0.01
Exp. Peak Ratio:       5.00
Exp. Adj. Ratio:       4.00
Exp. Val. Ratio:       3.00
RT Window:            30.0     sec
Expected RT:           1.26    min
Use Relative RT:       No

Int. Type:          Base To Base
Retention Time:        1.24    min
Area:             2.05e+004    counts
Height:             2.06e+003  cps
Start Time:            1.12    min
End Time:              1.77    min
```

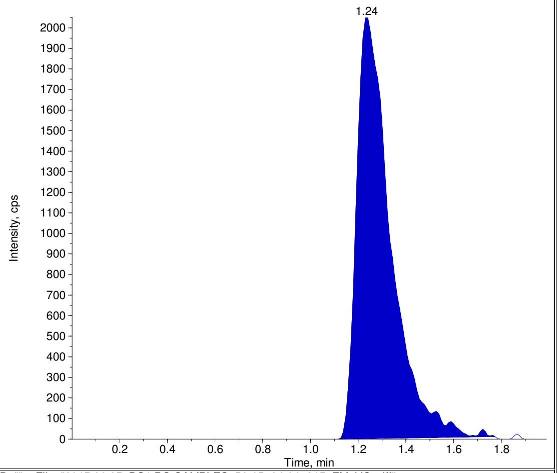

Sample Name: "DOUBLE BLANK" Sample ID: "" File: "2015 06 05_BC1 PQ SAMPLES_56-65_84-91_315_FM_VC.wiff"
Peak Name: "PQ1" Mass(es): "535.000/288.000 Da"
Comment: "" Annotation: ""

```
Sample Index:       44
Sample Type:        Double Blank
Concentration:      0.00      ng/mL
Calculated Conc:    N/A
Acq. Date:          6/5/2015
Acq. Time:          4:35:43 PM

Modified:              No
Proc. Algorithm: Analyst Classic
Bunching Factor:       6
Noise Threshold: 1.00e-6    cps
Area Threshold: 1.00e-6    cps
,Num. Smooths:          2
Sep. Width:            0.20
Sep. Height:           0.01
Exp. Peak Ratio:       5.00
Exp. Adj. Ratio:       4.00
Exp. Val. Ratio:       3.00
RT Window:            30.0     sec
Expected RT:           1.33    min
Use Relative RT:       No

Int. Type:          Base To Base
Retention Time:        1.36    min
Area:             4.67e+002    counts
Height:             6.31e+001  cps
Start Time:            1.25    min
End Time:              1.48    min
```

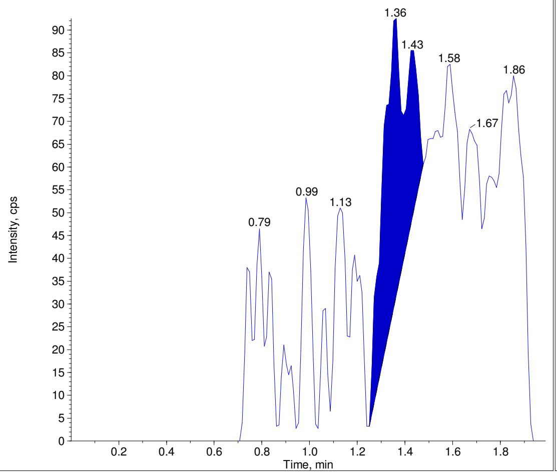

Sample Name: "DOUBLE BLANK" Sample ID: "" File: "2015 06 05_BC1 PQ SAMPLES_56-65_84-91_315_FM_VC.wiff"
Peak Name: "PQ_d6(IS)" Mass(es): "541.000/294.000 Da"
Comment: "" Annotation: ""

```
Sample Index:       44
Sample Type:        Double Blank
Concentration:      1.00      ng/mL
Calculated Conc:    N/A
Acq. Date:          6/5/2015
Acq. Time:          4:35:43 PM

Modified:              No
```

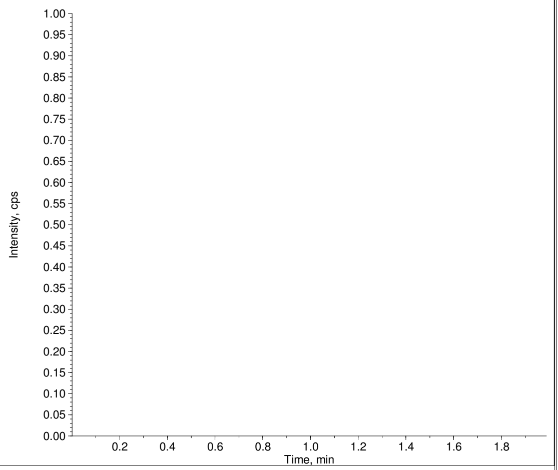

	Sample Name	Sample T	Acquisition Date	File Name	Analyte Peak Area (co	Analyte Conc
10	DOUBLE BLANK	Dou	7/30/2015	2015 07 30_BC2 PK Samples_29-42_VC.	0.00	0.00
11	DOUBLE BLANK	Dou	7/30/2015	2015 07 30_BC2 PK Samples_29-42_VC.	0.00	0.00
12	BLANK	Blan	7/30/2015	2015 07 30_BC2 PK Samples_29-42_VC.	0.00	0.00
13	BLANK	Blan	7/30/2015	2015 07 30_BC2 PK Samples_29-42_VC.	0.00	0.00
14	BLANK	Blan	7/30/2015	2015 07 30_BC2 PK Samples_29-42_VC.	0.00	0.00
15	DOUBLE BLANK	Dou	7/30/2015	2015 07 30_BC2 PK Samples_29-42_VC.	0.00	0.00
16	PQ STD 1	Sta	7/30/2015	2015 07 30_BC2 PK Samples_29-42_VC.	4870.	10.0
17	PQ STD 2	Sta	7/30/2015	2015 07 30_BC2 PK Samples_29-42_VC.	12000.	25.0
18	PQ STD 3	Sta	7/30/2015	2015 07 30_BC2 PK Samples_29-42_VC.	25200.	50.0
19	PQ STD 4	Sta	7/30/2015	2015 07 30_BC2 PK Samples_29-42_VC.	53300.	100.
20	PQ STD 5	Sta	7/30/2015	2015 07 30_BC2 PK Samples_29-42_VC.	127000.	250.
21	PQ STD 6	Sta	7/30/2015	2015 07 30_BC2 PK Samples_29-42_VC.	259000.	500.
22	PQ STD 7	Sta	7/30/2015	2015 07 30_BC2 PK Samples_29-42_VC.	503000.	1000.
23	DOUBLE BLANK	Dou	7/30/2015	2015 07 30_BC2 PK Samples_29-42_VC.	0.00	0.00
24	PQ QC LOW 1	Qua	7/30/2015	2015 07 30_BC2 PK Samples_29-42_VC.	16500.	30.0
25	PQ QC MEDIUM 1	Qua	7/30/2015	2015 07 30_BC2 PK Samples_29-42_VC.	99800.	200.
26	PQ QC HIGH 1	Qua	7/30/2015	2015 07 30_BC2 PK Samples_29-42_VC.	413000.	800.
27	DOUBLE BLANK	Dou	7/30/2015	2015 07 30_BC2 PK Samples_29-42_VC.	490.	0.00
28	29__B2-0013-AR__Day 2, predose, pk DP__I	Unk	7/30/2015	2015 07 30_BC2 PK Samples_29-42_VC.	24000.	N/A
29	30__B2-0013-AR__Day 2, 30min, pk DP__I2S	Unk	7/30/2015	2015 07 30_BC2 PK Samples_29-42_VC.	25300.	N/A
30	31__B2-0013-AR__Day 2, 1hr, pk DP__ISNP	Unk	7/30/2015	2015 07 30_BC2 PK Samples_29-42_VC.	23900.	N/A

Sample Name: "PQ STD 7" Sample ID: "" File: "2015 07 30_BC2 PK Samples_29-42_VC.wiff"
Peak Name: "PQ1" Mass(es): "535.000/288.000 Da"
Comment: "" Annotation: ""

```
Sample Index:       24
Sample Type:      Standard
Concentration:      1000.    ng/mL
Calculated Conc:     981.    ng/mL
Acq. Date:        7/30/2015
Acq. Time:        5:56:49 PM

Modified:            No
Proc. Algorithm: Analyst Classic
Bunching Factor:     5
Noise Threshold:     3.66   cps
Area Threshold:    18.29   cps
,Num. Smooths:        2
Sep. Width:          0.20
Sep. Height:         0.01
Exp. Peak Ratio:     5.00
Exp. Adj. Ratio:     4.00
Exp. Val. Ratio:     3.00
RT Window:          30.0    sec
Expected RT:         1.31   min
Use Relative RT:     No

Int. Type:        Base To Base
Retention Time:      1.20   min
Area:           503000.   counts
Height:         6.36e+004  cps
Start Time:          1.07   min
End Time:            1.99   min
```

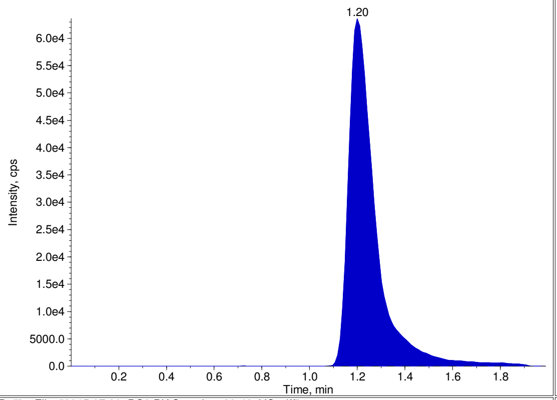

Sample Name: "PQ STD 7" Sample ID: "" File: "2015 07 30_BC2 PK Samples_29-42_VC.wiff"
Peak Name: "PQ_d6(IS)" Mass(es): "541.000/294.000 Da"
Comment: "" Annotation: ""

```
Sample Index:       24
Sample Type:      Standard
Concentration:       1.00    ng/mL
Calculated Conc:     N/A
Acq. Date:        7/30/2015
Acq. Time:        5:56:49 PM

Modified:            No
Proc. Algorithm: Analyst Classic
Bunching Factor:     6
Noise Threshold: 1.00e-6   cps
Area Threshold: 1.00e-6   cps
,Num. Smooths:        2
Sep. Width:          0.20
Sep. Height:         0.01
Exp. Peak Ratio:     5.00
Exp. Adj. Ratio:     4.00
Exp. Val. Ratio:     3.00
RT Window:          30.0    sec
Expected RT:         1.13   min
Use Relative RT:     No

Int. Type:        Base To Base
Retention Time:      1.15   min
Area:            12700.   counts
Height:         1.66e+003  cps
Start Time:          1.04   min
End Time:            1.99   min
```

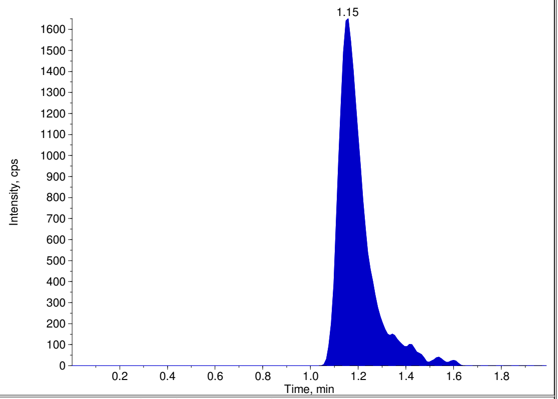

Sample Name: "DOUBLE BLANK" Sample ID: "" File: "2015 07 30_BC2 PK Samples_29-42_VC.wiff"
Peak Name: "PQ1" Mass(es): "535.000/288.000 Da"
Comment: "" Annotation: ""

```
Sample Index:       25
Sample Type:      Double Blank
Concentration:       0.00    ng/mL
Calculated Conc:     N/A
Acq. Date:        7/30/2015
Acq. Time:        6:00:29 PM

Modified:            No
```

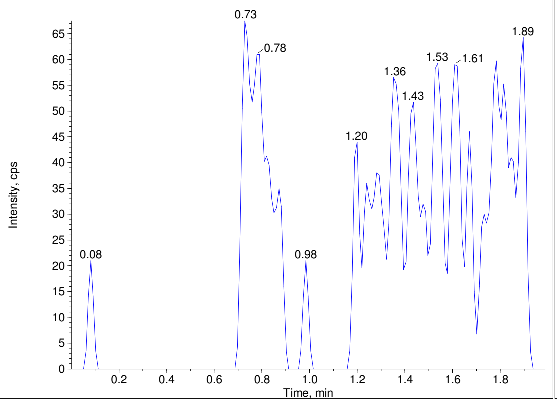

Sample Name: "DOUBLE BLANK" Sample ID: "" File: "2015 07 30_BC2 PK Samples_29-42_VC.wiff"
Peak Name: "PQ_d6(IS)" Mass(es): "541.000/294.000 Da"
Comment: "" Annotation: ""

```
Sample Index:       25
Sample Type:      Double Blank
Concentration:       1.00    ng/mL
Calculated Conc:     N/A
Acq. Date:        7/30/2015
Acq. Time:        6:00:29 PM

Modified:            No
```

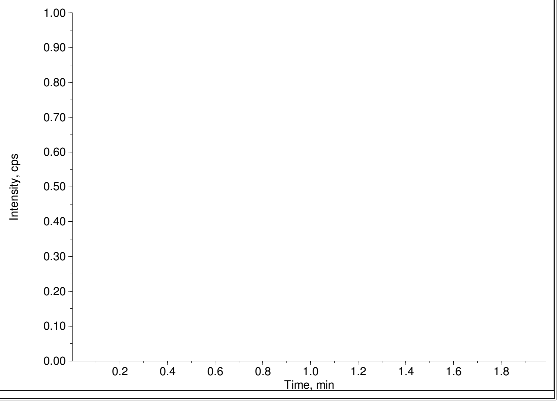

	Sample Name	Sample T	Acquisition Date	File Name	Analyte Peak Area (co	Analyte Conce	IS Peak Area (	Use R	Record Mo
15	BLANK	Blan	8/3/2015	2015 08 03_BC2 PK Samples_85-239_FM	0.00	0.00	16100.		☐
16	BLANK	Blan	8/3/2015	2015 08 03_BC2 PK Samples_85-239_FM	0.00	0.00	16300.		☐
17	DOUBLE BLANK	Dou	8/3/2015	2015 08 03_BC2 PK Samples_85-239_FM	0.00	0.00	0.00		☐
18	PQ STD 1	Sta	8/3/2015	2015 08 03_BC2 PK Samples_85-239_FM	6190.	10.0	23900.	☑	☐
19	PQ STD 2	Sta	8/3/2015	2015 08 03_BC2 PK Samples_85-239_FM	16100.	25.0	23300.	☑	☐
20	PQ STD 3	Sta	8/3/2015	2015 08 03_BC2 PK Samples_85-239_FM	32700.	50.0	23500.	☑	☐
21	PQ STD 4	Sta	8/3/2015	2015 08 03_BC2 PK Samples_85-239_FM	67200.	100.	24600.	☑	☐
22	PQ STD 5	Sta	8/3/2015	2015 08 03_BC2 PK Samples_85-239_FM	156000.	250.	22800.	☑	☐
23	PQ STD 6	Sta	8/3/2015	2015 08 03_BC2 PK Samples_85-239_FM	320000.	500.	22900.	☑	☐
24	PQ STD 7	Sta	8/3/2015	2015 08 03_BC2 PK Samples_85-239_FM	634000.	1000.	23300.	☑	☐
25	DOUBLE BLANK	Dou	8/3/2015	2015 08 03_BC2 PK Samples_85-239_FM	431.	0.00	0.00		☐
26	PQ QC LOW 1	Qua	8/3/2015	2015 08 03_BC2 PK Samples_85-239_FM	21300.	30.0	24600.	☑	☐
27	PQ QC MEDIUM 1	Qua	8/3/2015	2015 08 03_BC2 PK Samples_85-239_FM	132000.	200.	22600.	☑	☐
28	PQ QC HIGH 1	Qua	8/3/2015	2015 08 03_BC2 PK Samples_85-239_FM	517000.	800.	22400.	☑	☐

Sample Name: "PQ STD 7" Sample ID: "" File: "2015 08 03_BC2 PK Samples_85-239_FM.wiff"
Peak Name: "PQ1" Mass(es): "535.000/288.000 Da"
Comment: "" Annotation: ""

```
Sample Index:       24
Sample Type:        Standard
Concentration:      1000.    ng/mL
Calculated Conc:    991.     ng/mL
Acq. Date:          8/3/2015
Acq. Time:          12:13:29 PM

Modified:           No
Proc. Algorithm: Analyst Classic
Bunching Factor:    7
Noise Threshold: 1.00e-6   cps
Area Threshold: 1.00e-6    cps
,Num. Smooths:      2
Sep. Width:         0.20
Sep. Height:        0.01
Exp. Peak Ratio:    5.00
Exp. Adj. Ratio:    4.00
Exp. Val. Ratio:    3.00
RT Window:          30.0     sec
Expected RT:        1.21     min
Use Relative RT:    Yes

Int. Type:          Base To Base
Retention Time:     1.23     min
Area:               634000.  counts
Height:             8.00e+004 cps
Start Time:         1.07     min
End Time:           1.99     min
```

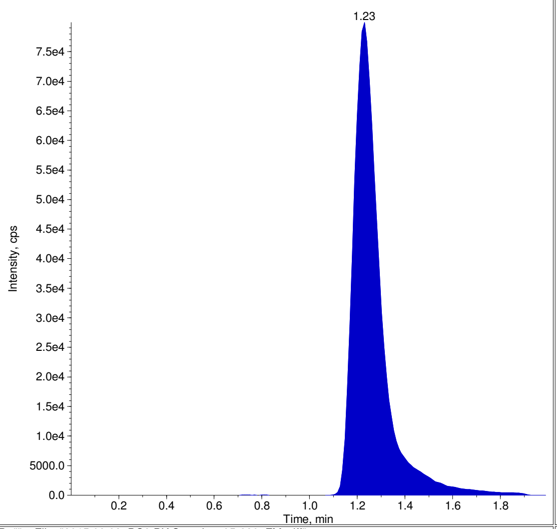

Sample Name: "PQ STD 7" Sample ID: "" File: "2015 08 03_BC2 PK Samples_85-239_FM.wiff"
Peak Name: "PQ_d6(IS)" Mass(es): "541.000/294.000 Da"
Comment: "" Annotation: ""

```
Sample Index:       24
Sample Type:        Standard
Concentration:      1.00     ng/mL
Calculated Conc:    N/A
Acq. Date:          8/3/2015
Acq. Time:          12:13:29 PM

Modified:           No
Proc. Algorithm: Analyst Classic
Bunching Factor:    7
Noise Threshold: 1.00e-6   cps
Area Threshold: 1.00e-6    cps
,Num. Smooths:      2
Sep. Width:         0.20
Sep. Height:        0.01
Exp. Peak Ratio:    5.00
Exp. Adj. Ratio:    4.00
Exp. Val. Ratio:    3.00
RT Window:          30.0     sec
Expected RT:        1.16     min
Use Relative RT:    No

Int. Type:          Base To Base
Retention Time:     1.17     min
Area:               23300.   counts
Height:             2.92e+003 cps
Start Time:         1.06     min
End Time:           1.99     min
```

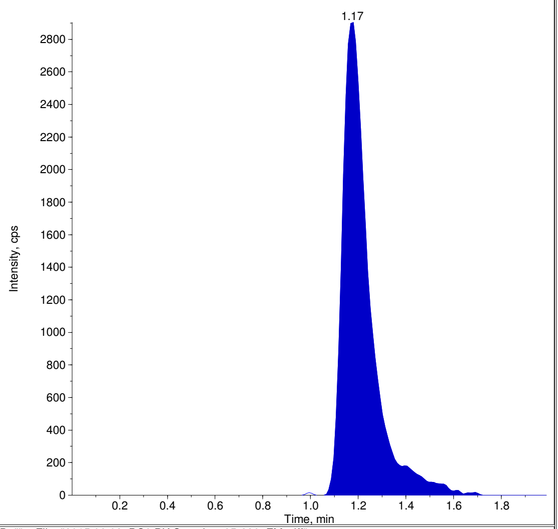

Sample Name: "DOUBLE BLANK" Sample ID: "" File: "2015 08 03_BC2 PK Samples_85-239_FM.wiff"
Peak Name: "PQ1" Mass(es): "535.000/288.000 Da"
Comment: "" Annotation: ""

```
Sample Index:       25
Sample Type:        Double Blank
Concentration:      0.00     ng/mL
Calculated Conc:    N/A
Acq. Date:          8/3/2015
Acq. Time:          12:17:09 PM

Modified:           No
Proc. Algorithm: Analyst Classic
Bunching Factor:    7
Noise Threshold: 1.00e-6   cps
Area Threshold: 1.00e-6    cps
,Num. Smooths:      2
Sep. Width:         0.20
Sep. Height:        0.01
Exp. Peak Ratio:    5.00
Exp. Adj. Ratio:    4.00
Exp. Val. Ratio:    3.00
RT Window:          30.0     sec
Expected RT:        1.21     min
Use Relative RT:    Yes

Int. Type:          Base To Base
Retention Time:     1.26     min
Area:               431.     counts
Height:             5.90e+001 cps
Start Time:         0.994    min
End Time:           1.36     min
```

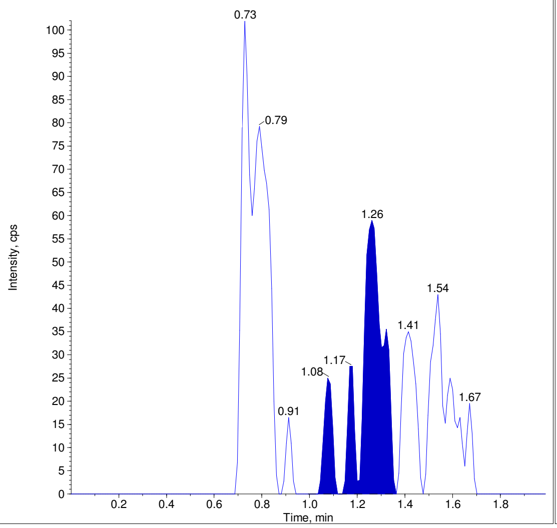

Sample Name: "DOUBLE BLANK" Sample ID: "" File: "2015 08 03_BC2 PK Samples_85-239_FM.wiff"
Peak Name: "PQ_d6(IS)" Mass(es): "541.000/294.000 Da"
Comment: "" Annotation: ""

```
Sample Index:       25
Sample Type:        Double Blank
Concentration:      1.00     ng/mL
Calculated Conc:    N/A
Acq. Date:          8/3/2015
Acq. Time:          12:17:09 PM

Modified:           No
```

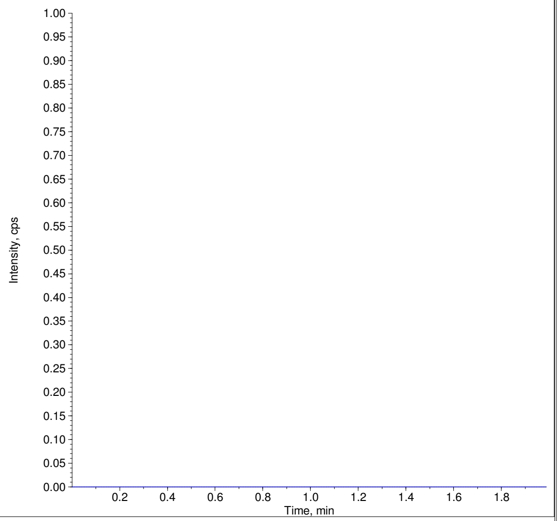

	Sample Name	Sample T	Acquisition Date	File Name	Analyte Peak Area (counts)	Analyte Conce	IS Peak Area (counts)	Use	Rec
13	PQ STD 4	Sta	1/26/2016 4:37:32 P	2016 01 26_BC1 Children_3-39_VC.	7.47e+004	100.	9.11e+003	☑	☐
14	PQ STD 5	Sta	1/26/2016 4:41:12 P	2016 01 26_BC1 Children_3-39_VC.	1.83e+005	250.	8.58e+003	☑	☐
15	PQ STD 6	Sta	1/26/2016 4:44:52 P	2016 01 26_BC1 Children_3-39_VC.	3.62e+005	500.	8.77e+003	☑	☐
16	PQ STD 7	Sta	1/26/2016 4:48:32 P	2016 01 26_BC1 Children_3-39_VC.	6.77e+005	1000.	8.35e+003	☑	☐
17	DOUBLE BLANK	Dou	1/26/2016 4:52:12 P	2016 01 26_BC1 Children_3-39_VC.	7.80e+002	0.00	0.00e+000		☐
18	PQ QC LOW 1	Qua	1/26/2016 4:55:47 P	2016 01 26_BC1 Children_3-39_VC.	2.28e+004	30.0	8.97e+003	☑	☐
19	PQ QC MEDIUM 1	Qua	1/26/2016 4:59:22 P	2016 01 26_BC1 Children_3-39_VC.	1.43e+005	200.	8.63e+003	☑	☐
20	PQ QC HIGH 1	Qua	1/26/2016 5:02:57 P	2016 01 26_BC1 Children_3-39_VC.	5.57e+005	800.	8.18e+003	☑	☐
21	DOUBLE BLANK	Dou	1/26/2016 5:06:32 P	2016 01 26_BC1 Children_3-39_VC.	4.26e+002	0.00	0.00e+000		☐
22	BC1 Children - 3	Unk	1/26/2016 5:10:07 P	2016 01 26_BC1 Children_3-39_VC.	1.57e+004	N/A	7.37e+003		☐
23	BC1 Children - 4	Unk	1/26/2016 5:13:42 P	2016 01 26_BC1 Children_3-39_VC.	1.85e+004	N/A	7.13e+003		☐
24	BC1 Children - 5	Unk	1/26/2016 5:17:21 P	2016 01 26_BC1 Children_3-39_VC.	5.72e+004	N/A	7.02e+003		☐
25	BC1 Children - 6	Unk	1/26/2016 5:20:57 P	2016 01 26_BC1 Children_3-39_VC.	7.85e+004	N/A	6.87e+003		☐

Sample Name: "PQ STD 7"   Sample ID: ""   File: "2016 01 26_BC1 Children_3-39_VC.wiff"
Peak Name: "PQ1"   Mass(es): "535.000/288.000 Da"
Comment: ""   Annotation: ""

```
Sample Index:       16
Sample Type:        Standard
Concentration:      1000.     ng/mL
Calculated Conc:    988.      ng/mL
Acq. Date:          1/26/2016
Acq. Time:          4:48:32 PM

Modified:           No
Proc. Algorithm: Analyst Classic
Bunching Factor:    7
Noise Threshold: 1.00e-6   cps
Area Threshold: 1.00e-6   cps
,Num. Smooths:      2
Sep. Width:         0.20
Sep. Height:        0.01
Exp. Peak Ratio:    5.00
Exp. Adj. Ratio:    4.00
Exp. Val. Ratio:    3.00
RT Window:          30.0    sec
Expected RT:        1.21    min
Use Relative RT:    Yes

Int. Type:          Base To Base
Retention Time:     1.19    min
Area:          6.77e+005    counts
Height:        9.88e+004    cps
Start Time:         0.994   min
End Time:           1.99    min
```

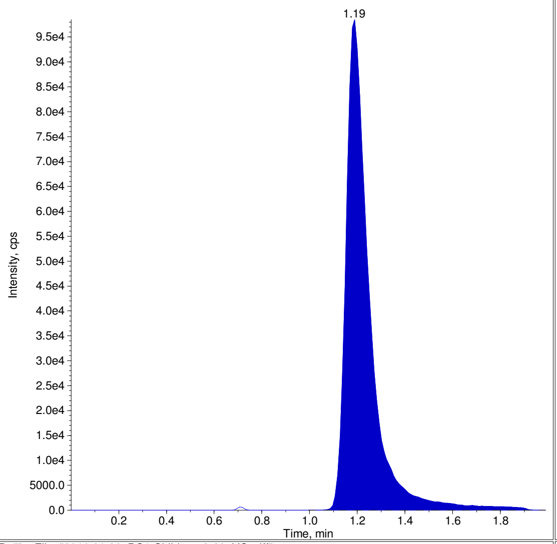

Sample Name: "PQ STD 7"   Sample ID: ""   File: "2016 01 26_BC1 Children_3-39_VC.wiff"
Peak Name: "PQ_d6(IS)"   Mass(es): "541.000/294.000 Da"
Comment: ""   Annotation: ""

```
Sample Index:       16
Sample Type:        Standard
Concentration:      1.00      ng/mL
Calculated Conc:    N/A
Acq. Date:          1/26/2016
Acq. Time:          4:48:32 PM

Modified:           No
Proc. Algorithm: Analyst Classic
Bunching Factor:    7
Noise Threshold: 1.00e-6   cps
Area Threshold: 1.00e-6   cps
,Num. Smooths:      2
Sep. Width:         0.20
Sep. Height:        0.01
Exp. Peak Ratio:    5.00
Exp. Adj. Ratio:    4.00
Exp. Val. Ratio:    3.00
RT Window:          30.0    sec
Expected RT:        1.16    min
Use Relative RT:    No

Int. Type:          Base To Base
Retention Time:     1.15    min
Area:          8.35e+003    counts
Height:        1.27e+003    cps
Start Time:         0.994   min
End Time:           1.99    min
```

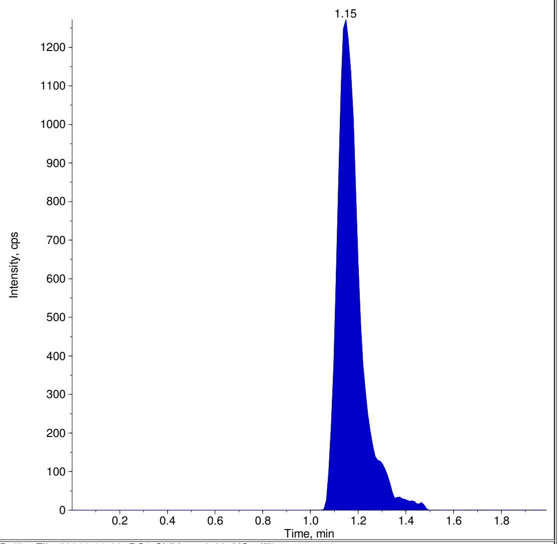

Sample Name: "DOUBLE BLANK"   Sample ID: ""   File: "2016 01 26_BC1 Children_3-39_VC.wiff"
Peak Name: "PQ1"   Mass(es): "535.000/288.000 Da"
Comment: ""   Annotation: ""

```
Sample Index:       17
Sample Type:        Double Blank
Concentration:      0.00      ng/mL
Calculated Conc:    N/A
Acq. Date:          1/26/2016
Acq. Time:          4:52:12 PM

Modified:           No
Proc. Algorithm: Analyst Classic
Bunching Factor:    7
Noise Threshold: 1.00e-6   cps
Area Threshold: 1.00e-6   cps
,Num. Smooths:      2
Sep. Width:         0.20
Sep. Height:        0.01
Exp. Peak Ratio:    5.00
Exp. Adj. Ratio:    4.00
Exp. Val. Ratio:    3.00
RT Window:          30.0    sec
Expected RT:        1.21    min
Use Relative RT:    Yes

Int. Type:          Base To Base
Retention Time:     1.21    min
Area:          7.80e+002    counts
Height:        6.87e+001    cps
Start Time:         1.07    min
End Time:           1.57    min
```

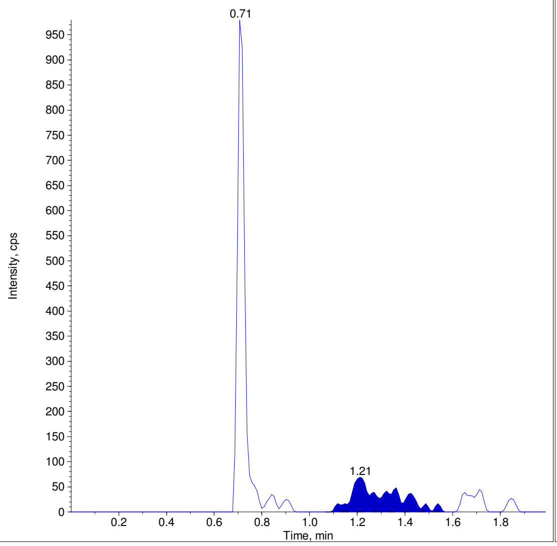

Sample Name: "DOUBLE BLANK"   Sample ID: ""   File: "2016 01 26_BC1 Children_3-39_VC.wiff"
Peak Name: "PQ_d6(IS)"   Mass(es): "541.000/294.000 Da"
Comment: ""   Annotation: ""

```
Sample Index:       17
Sample Type:        Double Blank
Concentration:      1.00      ng/mL
Calculated Conc:    N/A
Acq. Date:          1/26/2016
Acq. Time:          4:52:12 PM

Modified:           No
```

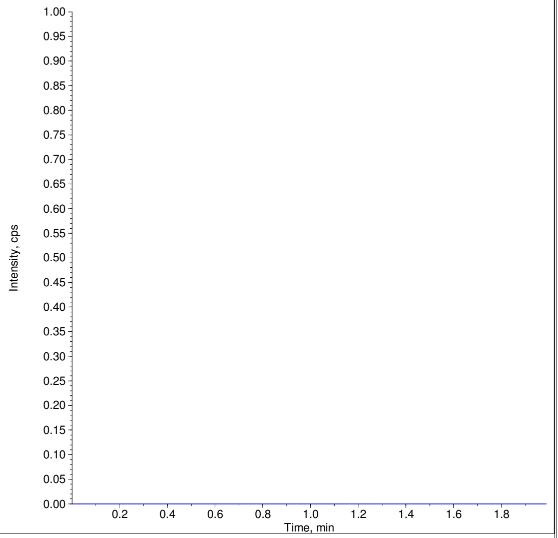

	Sample Name	Sample T	Acquisition Date	File Name	Analyte Peak Area (counts)	Analyte Conc	IS Peak Area (counts)	Use	Rec
1	SYSTEM SUITABILITY 1	Unk	1/28/2016 11:52:28 A	2016 01 27_BC1 Children_47-130_VC	3.30e+005	N/A	7.46e+003		☐
2	SYSTEM SUITABILITY 2	Unk	1/28/2016 11:56:03 A	2016 01 27_BC1 Children_47-130_VC	2.97e+005	N/A	6.12e+003		☐
3	SYSTEM SUITABILITY 3	Unk	1/28/2016 11:59:38 A	2016 01 27_BC1 Children_47-130_VC	2.73e+005	N/A	6.20e+003		☐
4	SYSTEM SUITABILITY 4	Unk	1/28/2016 12:03:13 P	2016 01 27_BC1 Children_47-130_VC	3.26e+005	N/A	6.69e+003		☐
5	SYSTEM SUITABILITY 5	Unk	1/28/2016 12:06:48 P	2016 01 27_BC1 Children_47-130_VC	3.31e+005	N/A	7.06e+003		☐
6	SYSTEM SUITABILITY 6	Dou	1/28/2016 12:10:23 P	2016 01 27_BC1 Children_47-130_VC	3.12e+005	0.00	6.87e+003		☐
7	DOUBLE BLANK	Dou	1/28/2016 12:13:59 P	2016 01 27_BC1 Children_47-130_VC	6.93e+002	0.00	0.00e+000		☐
8	BLANK	Blan	1/28/2016 12:17:36 P	2016 01 27_BC1 Children_47-130_VC	0.00e+000	0.00	5.93e+003		☐
9	DOUBLE BLANK	Dou	1/28/2016 12:21:13 P	2016 01 27_BC1 Children_47-130_VC	0.00e+000	0.00	0.00e+000		☐
10	PQ STD 1	Sta	1/28/2016 12:24:49 P	2016 01 27_BC1 Children_47-130_VC	5.07e+003	10.0	5.85e+003	☑	☐
11	PQ STD 2	Sta	1/28/2016 12:28:26 P	2016 01 27_BC1 Children_47-130_VC	1.43e+004	25.0	6.06e+003	☑	☐
12	PQ STD 3	Sta	1/28/2016 12:32:04 P	2016 01 27_BC1 Children_47-130_VC	2.66e+004	50.0	6.04e+003	☑	☐
13	PQ STD 4	Sta	1/28/2016 12:35:44 P	2016 01 27_BC1 Children_47-130_VC	5.28e+004	100.	6.12e+003	☑	☐
14	PQ STD 5	Sta	1/28/2016 12:39:24 P	2016 01 27_BC1 Children_47-130_VC	1.31e+005	250.	5.79e+003	☑	☐
15	PQ STD 6	Sta	1/28/2016 12:43:04 P	2016 01 27_BC1 Children_47-130_VC	2.60e+005	500.	5.85e+003	☑	☐
16	PQ STD 7	Sta	1/28/2016 12:46:44 P	2016 01 27_BC1 Children_47-130_VC	4.58e+005	1000.	5.49e+003	☑	☐
17	DOUBLE BLANK	Dou	1/28/2016 12:50:24 P	2016 01 27_BC1 Children_47-130_VC	0.00e+000	0.00	0.00e+000		☐
18	PQ QC LOW 1	Qua	1/28/2016 12:53:59 P	2016 01 27_BC1 Children_47-130_VC	1.72e+004	30.0	6.09e+003	☑	☐
19	PQ QC MEDIUM 1	Qua	1/28/2016 12:57:34 P	2016 01 27_BC1 Children_47-130_VC	1.15e+005	200.	6.18e+003	☑	☐
20	PQ QC HIGH 1	Qua	1/28/2016 1:01:09 P	2016 01 27_BC1 Children_47-130_VC	4.11e+005	800.	5.50e+003	☑	☐
21	DOUBLE BLANK	Dou	1/28/2016 1:04:44 P	2016 01 27_BC1 Children_47-130_VC	8.86e+002	0.00	0.00e+000		☐

Sample Name: "PQ STD 7" Sample ID: "" File: "2016 01 27_BC1 Children_47-130_VC.wiff"
Peak Name: "PQ1" Mass(es): "535.000/288.000 Da"
Comment: "" Annotation: ""

```
Sample Index:      114
Sample Type:       Standard
Concentration:     1000.    ng/mL
Calculated Conc:   971.     ng/mL
Acq. Date:         1/28/2016
Acq. Time:         12:46:44 PM

Modified:          No
Proc. Algorithm: Analyst Classic
Bunching Factor:   7
Noise Threshold: 1.00e-6   cps
Area Threshold: 1.00e-6   cps
,Num. Smooths:     2
Sep. Width:        0.20
Sep. Height:       0.01
Exp. Peak Ratio:   5.00
Exp. Adj. Ratio:   4.00
Exp. Val. Ratio:   3.00
RT Window:         30.0     sec
Expected RT:       1.21     min
Use Relative RT:   Yes

Int. Type:         Base To Base
Retention Time:    1.09     min
Area:              4.58e+005   counts
Height:            1.01e+005   cps
Start Time:        0.943    min
End Time:          1.70     min
```

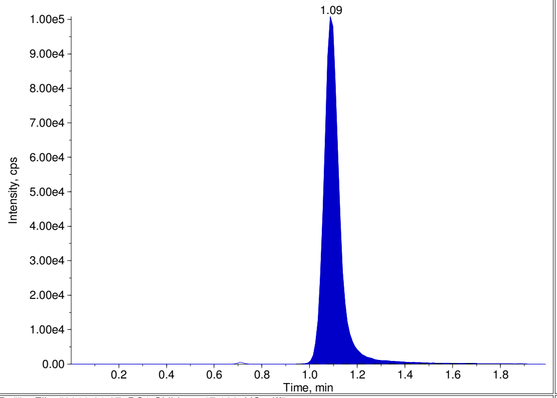

Sample Name: "PQ STD 7" Sample ID: "" File: "2016 01 27_BC1 Children_47-130_VC.wiff"
Peak Name: "PQ_d6(IS)" Mass(es): "541.000/294.000 Da"
Comment: "" Annotation: ""

```
Sample Index:      114
Sample Type:       Standard
Concentration:     1.00     ng/mL
Calculated Conc:   N/A
Acq. Date:         1/28/2016
Acq. Time:         12:46:44 PM

Modified:          No
Proc. Algorithm: Analyst Classic
Bunching Factor:   7
Noise Threshold: 1.00e-6   cps
Area Threshold: 1.00e-6   cps
,Num. Smooths:     2
Sep. Width:        0.20
Sep. Height:       0.01
Exp. Peak Ratio:   5.00
Exp. Adj. Ratio:   4.00
Exp. Val. Ratio:   3.00
RT Window:         30.0     sec
Expected RT:       1.16     min
Use Relative RT:   No

Int. Type:         Base To Base
Retention Time:    1.04     min
Area:              5.49e+003   counts
Height:            1.12e+003   cps
Start Time:        0.923    min
End Time:          1.99     min
```

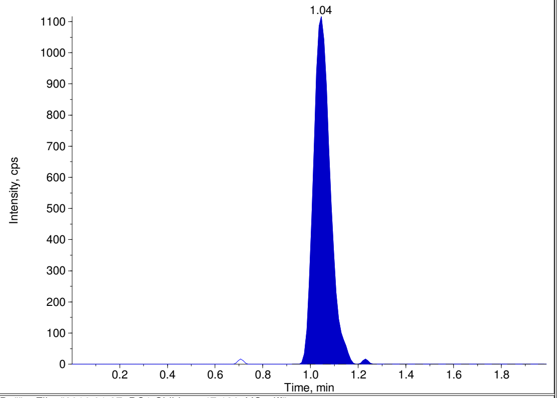

Sample Name: "DOUBLE BLANK" Sample ID: "" File: "2016 01 27_BC1 Children_47-130_VC.wiff"
Peak Name: "PQ1" Mass(es): "535.000/288.000 Da"
Comment: "" Annotation: ""

```
Sample Index:      115
Sample Type:       Double Blank
Concentration:     0.00     ng/mL
Calculated Conc:   N/A
Acq. Date:         1/28/2016
Acq. Time:         12:50:24 PM

Modified:          No
```

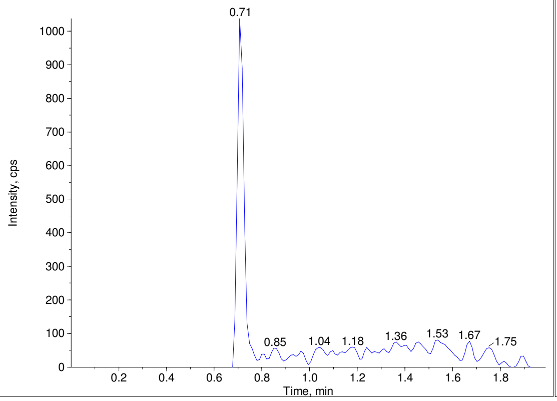

Sample Name: "DOUBLE BLANK" Sample ID: "" File: "2016 01 27_BC1 Children_47-130_VC.wiff"
Peak Name: "PQ_d6(IS)" Mass(es): "541.000/294.000 Da"
Comment: "" Annotation: ""

```
Sample Index:      115
Sample Type:       Double Blank
Concentration:     1.00     ng/mL
Calculated Conc:   N/A
Acq. Date:         1/28/2016
Acq. Time:         12:50:24 PM

Modified:          No
```

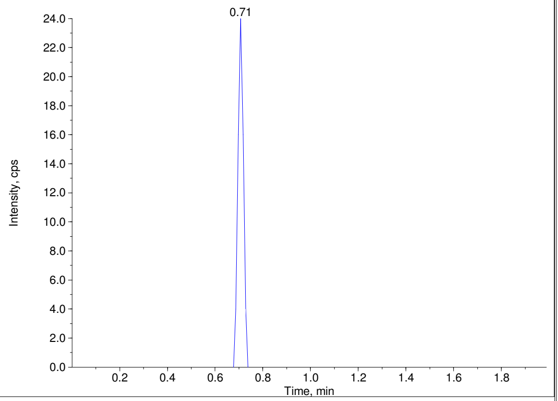

Polarity/Scan Type: N/A **API2000 S/N: H0079805 Printing Time: 10:05:52 AM
*Post PM Printing Date: Thursday, March 12, 2020

	Sample Name	Sa mp le T	Acquisition Date	File Name	Analyte Peak Area (counts)	Analy te Conc
1	SYSTEM SUITABILITY 1	Unk	2/4/2016 12:22:26 P	2016 02 04_BC1 Children_Nov Shipment_1-81_V	2.00e+005	N/A
2	SYSTEM SUITABILITY 2	Unk	2/4/2016 12:25:59 P	2016 02 04_BC1 Children_Nov Shipment_1-81_V	1.95e+005	N/A
3	SYSTEM SUITABILITY 3	Unk	2/4/2016 12:29:34 P	2016 02 04_BC1 Children_Nov Shipment_1-81_V	2.40e+005	N/A
4	SYSTEM SUITABILITY 4	Unk	2/4/2016 12:33:09 P	2016 02 04_BC1 Children_Nov Shipment_1-81_V	2.35e+005	N/A
5	SYSTEM SUITABILITY 5	Unk	2/4/2016 12:36:44 P	2016 02 04_BC1 Children_Nov Shipment_1-81_V	2.11e+005	N/A
6	SYSTEM SUITABILITY 6	Unk	2/4/2016 12:40:19 P	2016 02 04_BC1 Children_Nov Shipment_1-81_V	2.13e+005	N/A
7	DOUBLE BLANK	Dou	2/4/2016 12:43:54 P	2016 02 04_BC1 Children_Nov Shipment_1-81_V	4.77e+002	0.00
8	BLANK	Blan	2/4/2016 12:47:30 P	2016 02 04_BC1 Children_Nov Shipment_1-81_V	0.00e+000	0.00
9	DOUBLE BLANK	Dou	2/4/2016 12:51:04 P	2016 02 04_BC1 Children_Nov Shipment_1-81_V	0.00e+000	0.00
10	PQ STD 1	Sta	2/4/2016 12:54:39 P	2016 02 04_BC1 Children_Nov Shipment_1-81_V	3.35e+003	10.0
11	PQ STD 2	Sta	2/4/2016 12:58:15 P	2016 02 04_BC1 Children_Nov Shipment_1-81_V	9.98e+003	25.0
12	PQ STD 3	Sta	2/4/2016 1:01:50 PM	2016 02 04_BC1 Children_Nov Shipment_1-81_V	2.15e+004	50.0
13	PQ STD 4	Sta	2/4/2016 1:05:25 PM	2016 02 04_BC1 Children_Nov Shipment_1-81_V	4.98e+004	100.
14	PQ STD 5	Sta	2/4/2016 1:09:05 PM	2016 02 04_BC1 Children_Nov Shipment_1-81_V	1.27e+005	250.
15	PQ STD 6	Sta	2/4/2016 1:12:45 PM	2016 02 04_BC1 Children_Nov Shipment_1-81_V	2.71e+005	500.
16	PQ STD 7	Sta	2/4/2016 1:16:25 PM	2016 02 04_BC1 Children_Nov Shipment_1-81_V	5.20e+005	1000.
17	DOUBLE BLANK	Dou	2/4/2016 1:20:05 PM	2016 02 04_BC1 Children_Nov Shipment_1-81_V	3.69e+002	0.00
18	PQ QC LOW 1	Qua	2/4/2016 1:23:40 PM	2016 02 04_BC1 Children_Nov Shipment_1-81_V	1.49e+004	30.0
19	PQ QC MEDIUM 1	Qua	2/4/2016 1:27:15 PM	2016 02 04_BC1 Children_Nov Shipment_1-81_V	1.02e+005	200.
20	PQ QC HIGH 1	Qua	2/4/2016 1:30:50 PM	2016 02 04_BC1 Children_Nov Shipment_1-81_V	4.15e+005	800.

Sample Name: "PQ STD 7" Sample ID: "" File: "2016 02 04_BC1 Children_Nov Shipment_1-81_VC.wiff"
Peak Name: "PQ1" Mass(es): "535.000/288.000 Da"
Comment: "" Annotation: ""

```
Sample Index:       16
Sample Type:        Standard
Concentration:      1000.    ng/mL
Calculated Conc:    980.     ng/mL
Acq. Date:          2/4/2016
Acq. Time:          1:16:25 PM

Modified:           No
Proc. Algorithm: Analyst Classic
Bunching Factor:    7
Noise Threshold: 1.00e-6    cps
Area Threshold: 1.00e-6    cps
,Num. Smooths:      2
Sep. Width:         0.20
Sep. Height:        0.01
Exp. Peak Ratio:    5.00
Exp. Adj. Ratio:    4.00
Exp. Val. Ratio:    3.00
RT Window:          30.0     sec
Expected RT:        1.21     min
Use Relative RT:    Yes

Int. Type:          Base To Base
Retention Time:     1.12     min
Area:               5.20e+005    counts
Height:             9.80e+004    cps
Start Time:         0.923    min
End Time:           1.99     min
```

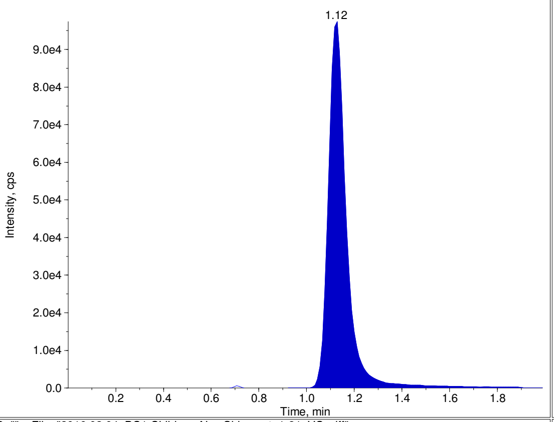

Sample Name: "PQ STD 7" Sample ID: "" File: "2016 02 04_BC1 Children_Nov Shipment_1-81_VC.wiff"
Peak Name: "PQ_d6(IS)" Mass(es): "541.000/294.000 Da"
Comment: "" Annotation: ""

```
Sample Index:       16
Sample Type:        Standard
Concentration:      1.00     ng/mL
Calculated Conc:    N/A
Acq. Date:          2/4/2016
Acq. Time:          1:16:25 PM

Modified:           No
Proc. Algorithm: Analyst Classic
Bunching Factor:    7
Noise Threshold: 1.00e-6    cps
Area Threshold: 1.00e-6    cps
,Num. Smooths:      2
Sep. Width:         0.20
Sep. Height:        0.01
Exp. Peak Ratio:    5.00
Exp. Adj. Ratio:    4.00
Exp. Val. Ratio:    3.00
RT Window:          30.0     sec
Expected RT:        1.16     min
Use Relative RT:    No

Int. Type:          Base To Base
Retention Time:     1.07     min
Area:               5.68e+003    counts
Height:             1.09e+003    cps
Start Time:         0.923    min
End Time:           1.99     min
```

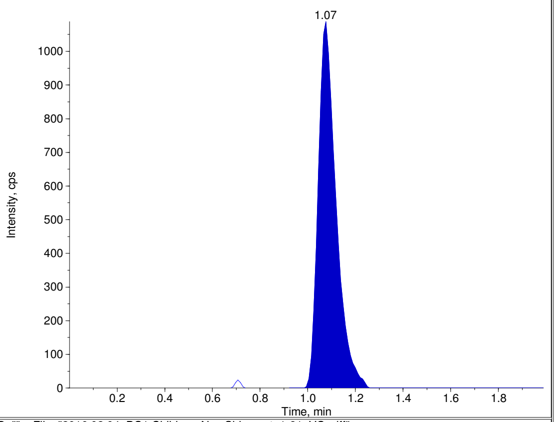

Sample Name: "DOUBLE BLANK" Sample ID: "" File: "2016 02 04_BC1 Children_Nov Shipment_1-81_VC.wiff"
Peak Name: "PQ1" Mass(es): "535.000/288.000 Da"
Comment: "" Annotation: ""

```
Sample Index:       17
Sample Type:        Double Blank
Concentration:      0.00     ng/mL
Calculated Conc:    N/A
Acq. Date:          2/4/2016
Acq. Time:          1:20:05 PM

Modified:           No
Proc. Algorithm: Analyst Classic
Bunching Factor:    7
Noise Threshold: 1.00e-6    cps
Area Threshold: 1.00e-6    cps
,Num. Smooths:      2
Sep. Width:         0.20
Sep. Height:        0.01
Exp. Peak Ratio:    5.00
Exp. Adj. Ratio:    4.00
Exp. Val. Ratio:    3.00
RT Window:          30.0     sec
Expected RT:        1.21     min
Use Relative RT:    Yes

Int. Type:          Valley
Retention Time:     1.15     min
Area:               3.69e+002    counts
Height:             6.30e+001    cps
Start Time:         1.06     min
End Time:           1.22     min
```

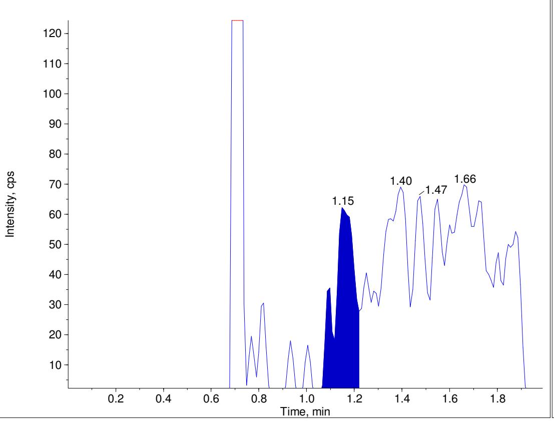

Sample Name: "DOUBLE BLANK" Sample ID: "" File: "2016 02 04_BC1 Children_Nov Shipment_1-81_VC.wiff"
Peak Name: "PQ_d6(IS)" Mass(es): "541.000/294.000 Da"
Comment: "" Annotation: ""

```
Sample Index:       17
Sample Type:        Double Blank
Concentration:      1.00     ng/mL
Calculated Conc:    N/A
Acq. Date:          2/4/2016
Acq. Time:          1:20:05 PM

Modified:           No
```

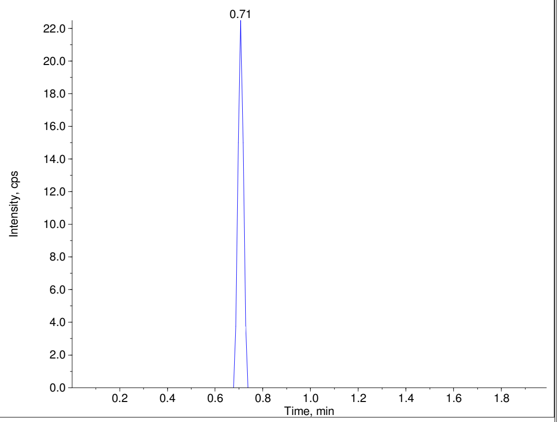

	Sample Name	Sample T	Acquisition Date	File Name	Analyte Peak Area (co	Analyte Conce	IS Peak Area (counts)
11	DOUBLE BLANK	Dou	3/1/2016 1	2016 03 01_BC1 PQ Control SAMPLES_211-278_FM.	0.00	0.00	0.00
12	DOUBLE BLANK	Dou	3/1/2016 1	2016 03 01_BC1 PQ Control SAMPLES_211-278_FM.	0.00	0.00	0.00
13	BLANK	Blan	3/1/2016 1	2016 03 01_BC1 PQ Control SAMPLES_211-278_FM.	0.00	0.00	19200.
14	BLANK	Blan	3/1/2016 1	2016 03 01_BC1 PQ Control SAMPLES_211-278_FM.	0.00	0.00	19300.
15	BLANK	Blan	3/1/2016 1	2016 03 01_BC1 PQ Control SAMPLES_211-278_FM.	0.00	0.00	19800.
16	DOUBLE BLANK	Dou	3/1/2016 1	2016 03 01_BC1 PQ Control SAMPLES_211-278_FM.	0.00	0.00	0.00
17	PQ STD 1	Sta	3/1/2016 1	2016 03 01_BC1 PQ Control SAMPLES_211-278_FM.	6350.	10.0	22700.
18	PQ STD 2	Sta	3/1/2016 1	2016 03 01_BC1 PQ Control SAMPLES_211-278_FM.	15700.	25.0	22300.
19	PQ STD 3	Sta	3/1/2016 1	2016 03 01_BC1 PQ Control SAMPLES_211-278_FM.	31200.	50.0	23000.
20	PQ STD 4	Sta	3/1/2016 1	2016 03 01_BC1 PQ Control SAMPLES_211-278_FM.	62400.	100.	23600.
21	PQ STD 5	Sta	3/1/2016 1	2016 03 01_BC1 PQ Control SAMPLES_211-278_FM.	155000.	250.	23700.
22	PQ STD 6	Sta	3/1/2016 1	2016 03 01_BC1 PQ Control SAMPLES_211-278_FM.	295000.	500.	22400.
23	PQ STD 7	Sta	3/1/2016 1	2016 03 01_BC1 PQ Control SAMPLES_211-278_FM.	619000.	1000.	23500.
24	DOUBLE BLANK	Dou	3/1/2016 1	2016 03 01_BC1 PQ Control SAMPLES_211-278_FM.	1070.	0.00	0.00
25	PQ QC LOW 1	Qua	3/1/2016 1	2016 03 01_BC1 PQ Control SAMPLES_211-278_FM.	20800.	30.0	24000.
26	PQ QC MEDIUM 1	Qua	3/1/2016 1	2016 03 01_BC1 PQ Control SAMPLES_211-278_FM.	114000.	200.	21100.
27	PQ QC HIGH 1	Qua	3/1/2016 1	2016 03 01_BC1 PQ Control SAMPLES_211-278_FM.	498000.	800.	22000.

Sample Name: "PQ STD 7" Sample ID: "" File: "2016 03 01_BC1 PQ Control SAMPLES_211-278_FM.wiff"
Peak Name: "PQ1" Mass(es): "535.000/288.000 Da"
Comment: "" Annotation: ""

```
Sample Index:       23
Sample Type:       Standard
Concentration:      1000.    ng/mL
Calculated Conc:    1000.    ng/mL
Acq. Date:         3/1/2016
Acq. Time:         11:36:41 AM

Modified:            No
Proc. Algorithm: Analyst Classic
Bunching Factor:     7
Noise Threshold: 1.00e-6   cps
Area Threshold: 1.00e-6   cps
,Num. Smooths:        2
Sep. Width:          0.20
Sep. Height:         0.01
Exp. Peak Ratio:     5.00
Exp. Adj. Ratio:     4.00
Exp. Val. Ratio:     3.00
RT Window:          30.0    sec
Expected RT:         1.21   min
Use Relative RT:     Yes

Int. Type:         Base To Base
Retention Time:      1.23   min
Area:           619000.   counts
Height:           9.07e+004  cps
Start Time:         0.994   min
End Time:            1.99   min
```

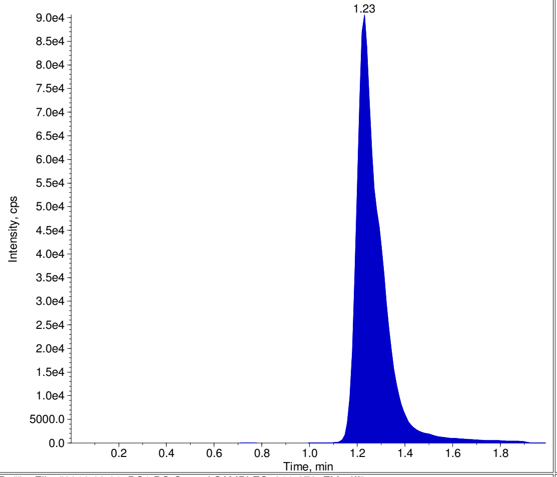

Sample Name: "PQ STD 7" Sample ID: "" File: "2016 03 01_BC1 PQ Control SAMPLES_211-278_FM.wiff"
Peak Name: "PQ_d6(IS)" Mass(es): "541.000/294.000 Da"
Comment: "" Annotation: ""

```
Sample Index:       23
Sample Type:       Standard
Concentration:       1.00    ng/mL
Calculated Conc:    N/A
Acq. Date:         3/1/2016
Acq. Time:         11:36:41 AM

Modified:            No
Proc. Algorithm: Analyst Classic
Bunching Factor:     7
Noise Threshold: 1.00e-6   cps
Area Threshold: 1.00e-6   cps
,Num. Smooths:        2
Sep. Width:          0.20
Sep. Height:         0.01
Exp. Peak Ratio:     5.00
Exp. Adj. Ratio:     4.00
Exp. Val. Ratio:     3.00
RT Window:          30.0    sec
Expected RT:         1.16   min
Use Relative RT:     No

Int. Type:         Base To Base
Retention Time:      1.18   min
Area:            23500.   counts
Height:           3.77e+003  cps
Start Time:          1.07   min
End Time:            1.64   min
```

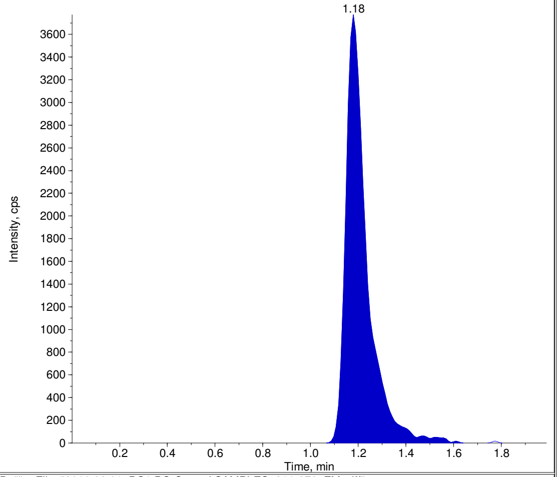

Sample Name: "DOUBLE BLANK" Sample ID: "" File: "2016 03 01_BC1 PQ Control SAMPLES_211-278_FM.wiff"
Peak Name: "PQ1" Mass(es): "535.000/288.000 Da"
Comment: "" Annotation: ""

```
Sample Index:       24
Sample Type:       Double Blank
Concentration:       0.00    ng/mL
Calculated Conc:    N/A
Acq. Date:         3/1/2016
Acq. Time:         11:40:21 AM

Modified:            No
Proc. Algorithm: Analyst Classic
Bunching Factor:     7
Noise Threshold: 1.00e-6   cps
Area Threshold: 1.00e-6   cps
,Num. Smooths:        2
Sep. Width:          0.20
Sep. Height:         0.01
Exp. Peak Ratio:     5.00
Exp. Adj. Ratio:     4.00
Exp. Val. Ratio:     3.00
RT Window:          30.0    sec
Expected RT:         1.21   min
Use Relative RT:     Yes

Int. Type:         Valley
Retention Time:      1.36   min
Area:             1070.   counts
Height:           6.40e+001  cps
Start Time:          1.14   min
End Time:            1.66   min
```

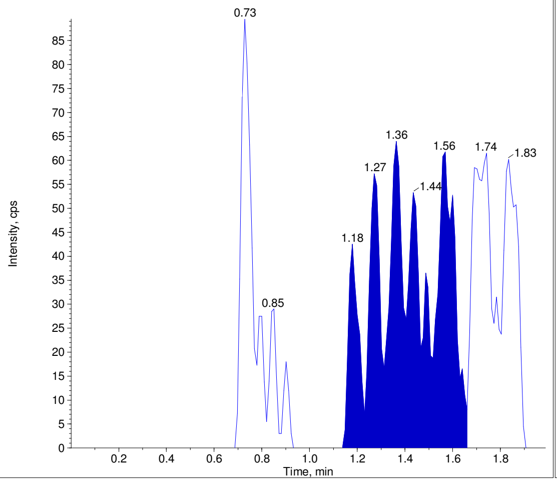

Sample Name: "DOUBLE BLANK" Sample ID: "" File: "2016 03 01_BC1 PQ Control SAMPLES_211-278_FM.wiff"
Peak Name: "PQ_d6(IS)" Mass(es): "541.000/294.000 Da"
Comment: "" Annotation: ""

```
Sample Index:       24
Sample Type:       Double Blank
Concentration:       1.00    ng/mL
Calculated Conc:    N/A
Acq. Date:         3/1/2016
Acq. Time:         11:40:21 AM

Modified:            No
```

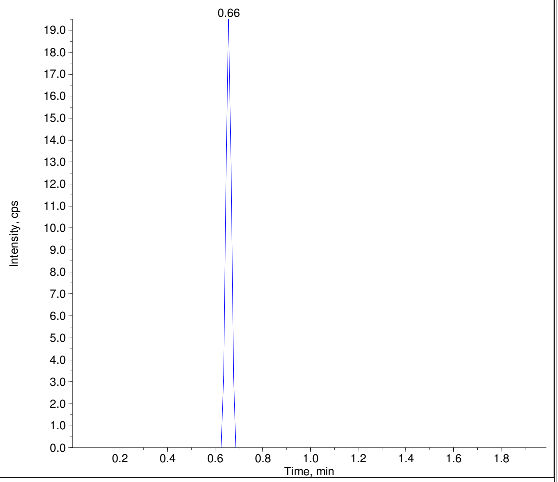

	Sample Name	Sample T	Acquisition Date	File Name	Analyte Peak Area (counts)	Analyte Conce	IS Peak Area (counts)	Use	Rec
5	BLANK	Blan	1/28/2016 6:02:22 P	2017 01 28_BC1 Children_134-214_VC	0.00e+000	0.00	5.64e+003		☐
6	DOUBLE BLANK	Dou	1/28/2016 6:05:57 P	2017 01 28_BC1 Children_134-214_VC	0.00e+000	0.00	0.00e+000		☐
7	PQ STD 1	Sta	1/28/2016 6:09:32 P	2017 01 28_BC1 Children_134-214_VC	5.16e+003	10.0	5.38e+003	☑	☐
8	PQ STD 2	Sta	1/28/2016 6:13:07 P	2017 01 28_BC1 Children_134-214_VC	1.42e+004	25.0	5.82e+003	☑	☐
9	PQ STD 3	Sta	1/28/2016 6:16:42 P	2017 01 28_BC1 Children_134-214_VC	2.60e+004	50.0	5.32e+003	☑	☐
10	PQ STD 4	Sta	1/28/2016 6:20:22 P	2017 01 28_BC1 Children_134-214_VC	5.02e+004	100.	5.66e+003	☑	☐
11	PQ STD 5	Sta	1/28/2016 6:24:02 P	2017 01 28_BC1 Children_134-214_VC	1.30e+005	250.	5.81e+003	☑	☐
12	PQ STD 6	Sta	1/28/2016 6:27:42 P	2017 01 28_BC1 Children_134-214_VC	2.46e+005	500.	5.41e+003	☑	☐
13	PQ STD 7	Sta	1/28/2016 6:31:22 P	2017 01 28_BC1 Children_134-214_VC	4.78e+005	1000.	5.53e+003	☑	☐
14	DOUBLE BLANK	Dou	1/28/2016 6:35:02 P	2017 01 28_BC1 Children_134-214_VC	4.26e+002	0.00	0.00e+000		☐
15	PQ QC LOW 1	Qua	1/28/2016 6:38:37 P	2017 01 28_BC1 Children_134-214_VC	1.62e+004	30.0	5.77e+003	☑	☐
16	PQ QC MEDIUM 1	Qua	1/28/2016 6:42:12 P	2017 01 28_BC1 Children_134-214_VC	1.11e+005	200.	5.77e+003	☑	☐
17	PQ QC HIGH 1	Qua	1/28/2016 6:45:47 P	2017 01 28_BC1 Children_134-214_VC	4.24e+005	800.	5.62e+003	☑	☐
18	DOUBLE BLANK	Dou	1/28/2016 6:49:22 P	2017 01 28_BC1 Children_134-214_VC	1.40e+002	0.00	0.00e+000		☐
19	BC1 Children - 134	Unk	1/28/2016 6:52:57 P	2017 01 28_BC1 Children_134-214_VC	2.04e+004	N/A	5.95e+003		☐
20	BC1 Children - 135	Unk	1/28/2016 6:56:32 P	2017 01 28_BC1 Children_134-214_VC	8.18e+004	N/A	6.15e+003		☐

Sample Name: "PQ STD 7" Sample ID: "" File: "2017 01 28_BC1 Children_134-214_VC.wiff"
Peak Name: "PQ1" Mass(es): "535.000/288.000 Da"
Comment: "" Annotation: ""

```
Sample Index:       13
Sample Type:        Standard
Concentration:      1000.    ng/mL
Calculated Conc:    980.     ng/mL
Acq. Date:          1/28/2016
Acq. Time:          6:31:22 PM

Modified:           No
Proc. Algorithm: Analyst Classic
Bunching Factor:    7
Noise Threshold: 1.00e-6   cps
Area Threshold: 1.00e-6   cps
,Num. Smooths:      2
Sep. Width:         0.20
Sep. Height:        0.01
Exp. Peak Ratio:    5.00
Exp. Adj. Ratio:    4.00
Exp. Val. Ratio:    3.00
RT Window:          30.0     sec
Expected RT:        1.21     min
Use Relative RT:    Yes

Int. Type:          Base To Base
Retention Time:     1.11     min
Area:          4.78e+005    counts
Height:        1.06e+005    cps
Start Time:         0.923    min
End Time:           1.99     min
```

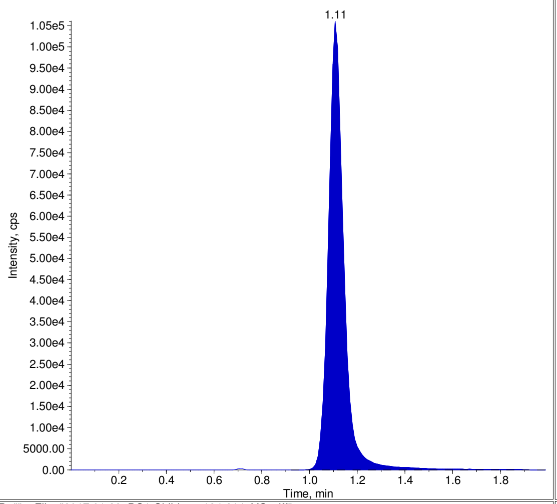

Sample Name: "PQ STD 7" Sample ID: "" File: "2017 01 28_BC1 Children_134-214_VC.wiff"
Peak Name: "PQ_d6(IS)" Mass(es): "541.000/294.000 Da"
Comment: "" Annotation: ""

```
Sample Index:       13
Sample Type:        Standard
Concentration:      1.00     ng/mL
Calculated Conc:    N/A
Acq. Date:          1/28/2016
Acq. Time:          6:31:22 PM

Modified:           No
Proc. Algorithm: Analyst Classic
Bunching Factor:    7
Noise Threshold: 1.00e-6   cps
Area Threshold: 1.00e-6   cps
,Num. Smooths:      2
Sep. Width:         0.20
Sep. Height:        0.01
Exp. Peak Ratio:    5.00
Exp. Adj. Ratio:    4.00
Exp. Val. Ratio:    3.00
RT Window:          30.0     sec
Expected RT:        1.16     min
Use Relative RT:    No

Int. Type:          Base To Base
Retention Time:     1.06     min
Area:          5.53e+003    counts
Height:        1.13e+003    cps
Start Time:         0.923    min
End Time:           1.99     min
```

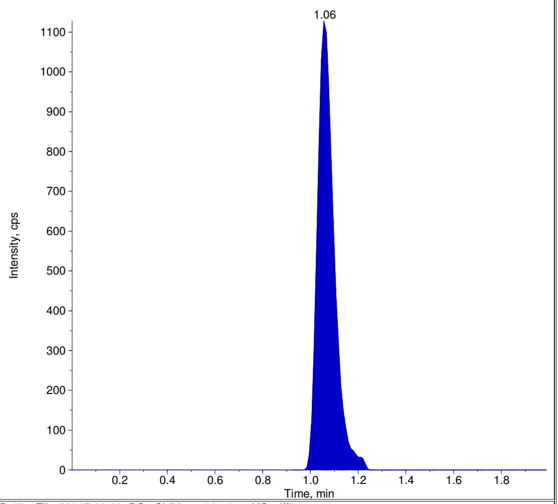

Sample Name: "DOUBLE BLANK" Sample ID: "" File: "2017 01 28_BC1 Children_134-214_VC.wiff"
Peak Name: "PQ1" Mass(es): "535.000/288.000 Da"
Comment: "" Annotation: ""

```
Sample Index:       14
Sample Type:        Double Blank
Concentration:      0.00     ng/mL
Calculated Conc:    N/A
Acq. Date:          1/28/2016
Acq. Time:          6:35:02 PM

Modified:           No
Proc. Algorithm: Analyst Classic
Bunching Factor:    7
Noise Threshold: 1.00e-6   cps
Area Threshold: 1.00e-6   cps
,Num. Smooths:      2
Sep. Width:         0.20
Sep. Height:        0.01
Exp. Peak Ratio:    5.00
Exp. Adj. Ratio:    4.00
Exp. Val. Ratio:    3.00
RT Window:          30.0     sec
Expected RT:        1.21     min
Use Relative RT:    Yes

Int. Type:          Base To Base
Retention Time:     1.15     min
Area:          4.26e+002    counts
Height:        4.83e+001    cps
Start Time:         0.994    min
End Time:           1.32     min
```

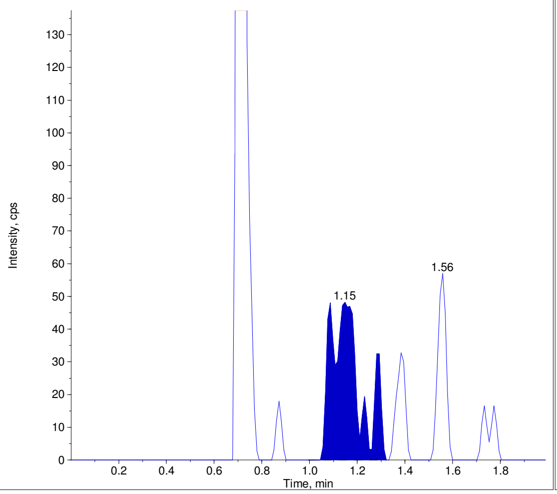

Sample Name: "DOUBLE BLANK" Sample ID: "" File: "2017 01 28_BC1 Children_134-214_VC.wiff"
Peak Name: "PQ_d6(IS)" Mass(es): "541.000/294.000 Da"
Comment: "" Annotation: ""

```
Sample Index:       14
Sample Type:        Double Blank
Concentration:      1.00     ng/mL
Calculated Conc:    N/A
Acq. Date:          1/28/2016
Acq. Time:          6:35:02 PM

Modified:           No
```

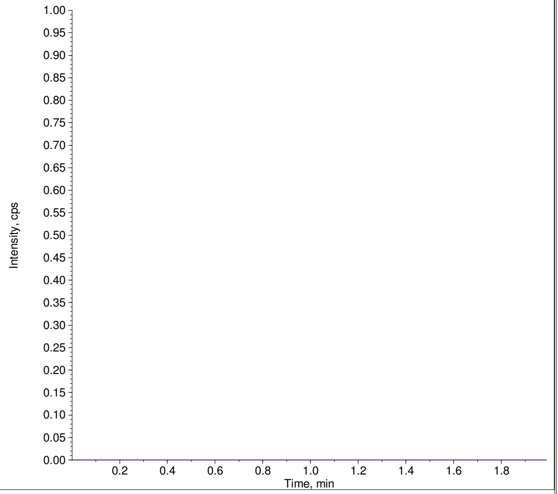

	Sample Name	Sample T	Acquisition Date	File Name	Analyte Peak Area (co	Analyte Conce	IS Peak Area (counts)	Use R
15	BLANK	Blan	3/7/2017 2:26:24 PM	2017 03 07_ PQ TEST_FM_AM.	0.00	0.00	24000.	
16	BLANK	Blan	3/7/2017 2:29:59 PM	2017 03 07_ PQ TEST_FM_AM.	0.00	0.00	23800.	
17	DOUBLE BLANK	Dou	3/7/2017 2:33:34 PM	2017 03 07_ PQ TEST_FM_AM.	0.00	0.00	0.00	
18	PQ STD 1	Unk	3/7/2017 2:37:09 PM	2017 03 07_ PQ TEST_FM_AM.	1680.	N/A	23900.	
19	PQ STD 2	Unk	3/7/2017 2:40:44 PM	2017 03 07_ PQ TEST_FM_AM.	4320.	N/A	23000.	
20	PQ STD 3	Unk	3/7/2017 2:44:19 PM	2017 03 07_ PQ TEST_FM_AM.	8470.	N/A	24300.	
21	PQ STD 4	Unk	3/7/2017 2:47:59 PM	2017 03 07_ PQ TEST_FM_AM.	17000.	N/A	24100.	
22	PQ STD 5	Unk	3/7/2017 2:51:39 PM	2017 03 07_ PQ TEST_FM_AM.	42900.	N/A	23100.	
23	PQ STD 6	Unk	3/7/2017 2:55:19 PM	2017 03 07_ PQ TEST_FM_AM.	90200.	N/A	24900.	
24	PQ STD 7	Unk	3/7/2017 2:58:59 PM	2017 03 07_ PQ TEST_FM_AM.	177000.	N/A	24700.	
25	DOUBLE BLANK	Unk	3/7/2017 3:02:39 PM	2017 03 07_ PQ TEST_FM_AM.	29.1	N/A	0.00	
26	DOUBLE BLANK	Unk	3/7/2017 3:06:14 PM	2017 03 07_ PQ TEST_FM_AM.	0.00	N/A	0.00	
27	PQ QC LOW 1	Unk	3/7/2017 3:09:50 PM	2017 03 07_ PQ TEST_FM_AM.	5770.	N/A	24100.	
28	PQ QC MEDIUM 1	Unk	3/7/2017 3:13:24 PM	2017 03 07_ PQ TEST_FM_AM.	38300.	N/A	24200.	
29	PQ QC HIGH 1	Unk	3/7/2017 3:16:59 PM	2017 03 07_ PQ TEST_FM_AM.	148000.	N/A	24400.	
30	DOUBLE BLANK	Dou	3/7/2017 3:20:34 PM	2017 03 07_ PQ TEST_FM_AM.	0.00	0.00	0.00	
31	DOUBLE BLANK	Dou	3/7/2017 3:24:10 PM	2017 03 07_ PQ TEST_FM_AM.	0.00	0.00	0.00	

Sample Name: "PQ STD 7" Sample ID: "" File: "2017 03 07_ PQ TEST_FM_AM.wiff"
Peak Name: "PQ1" Mass(es): "535.000/288.000 Da"
Comment: "" Annotation: ""

```
Sample Index:       27
Sample Type:      Unknown
Concentration:       N/A
Calculated Conc:   1010.    ng/mL
Acq. Date:        3/7/2017
Acq. Time:        2:58:59 PM

Modified:            No
Proc. Algorithm: Analyst Classic
Bunching Factor:     7
Noise Threshold: 1.00e-6   cps
Area Threshold: 1.00e-6   cps
,Num. Smooths:        2
Sep. Width:          0.20
Sep. Height:         0.01
Exp. Peak Ratio:     5.00
Exp. Adj. Ratio:     4.00
Exp. Val. Ratio:     3.00
RT Window:          30.0    sec
Expected RT:         1.21   min
Use Relative RT:     Yes

Int. Type:        Base To Base
Retention Time:      1.13   min
Area:           177000.   counts
Height:           3.14e+004  cps
Start Time:         0.994   min
End Time:            1.71   min
```

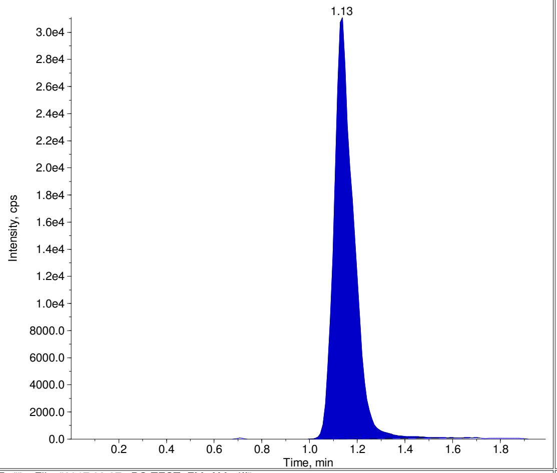

Sample Name: "PQ STD 7" Sample ID: "" File: "2017 03 07_ PQ TEST_FM_AM.wiff"
Peak Name: "PQ_d6(IS)" Mass(es): "541.000/294.000 Da"
Comment: "" Annotation: ""

```
Sample Index:       27
Sample Type:      Unknown
Concentration:       1.00    ng/mL
Calculated Conc:     N/A
Acq. Date:        3/7/2017
Acq. Time:        2:58:59 PM

Modified:            No
Proc. Algorithm: Analyst Classic
Bunching Factor:     7
Noise Threshold: 1.00e-6   cps
Area Threshold: 1.00e-6   cps
,Num. Smooths:        2
Sep. Width:          0.20
Sep. Height:         0.01
Exp. Peak Ratio:     5.00
Exp. Adj. Ratio:     4.00
Exp. Val. Ratio:     3.00
RT Window:          30.0    sec
Expected RT:         1.16   min
Use Relative RT:     No

Int. Type:        Base To Base
Retention Time:      1.08   min
Area:            24700.   counts
Height:           3.93e+003  cps
Start Time:         0.923   min
End Time:            1.42   min
```

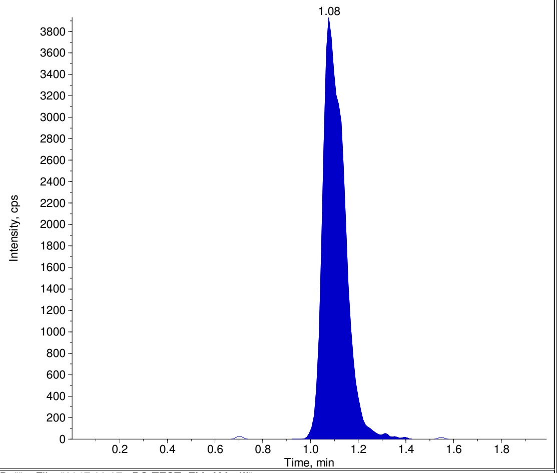

Sample Name: "DOUBLE BLANK" Sample ID: "" File: "2017 03 07_ PQ TEST_FM_AM.wiff"
Peak Name: "PQ1" Mass(es): "535.000/288.000 Da"
Comment: "" Annotation: ""

```
Sample Index:       28
Sample Type:      Unknown
Concentration:       N/A
Calculated Conc: No Intercept
Acq. Date:        3/7/2017
Acq. Time:        3:02:39 PM

Modified:            Yes
Proc. Algorithm: Analyst Classic
Bunching Factor:     7
Noise Threshold: 1.00e-6   cps
Area Threshold: 1.00e-6   cps
,Num. Smooths:        2
Sep. Width:          0.20
Sep. Height:         0.01
Exp. Peak Ratio:     5.00
Exp. Adj. Ratio:     4.00
Exp. Val. Ratio:     3.00
RT Window:          30.0    sec
Expected RT:         1.21   min
Use Relative RT:     Yes

Int. Type:         Manual
Retention Time:      1.17   min
Area:              29.1   counts
Height:          1.79e+001  cps
Start Time:          1.12   min
End Time:            1.21   min
```

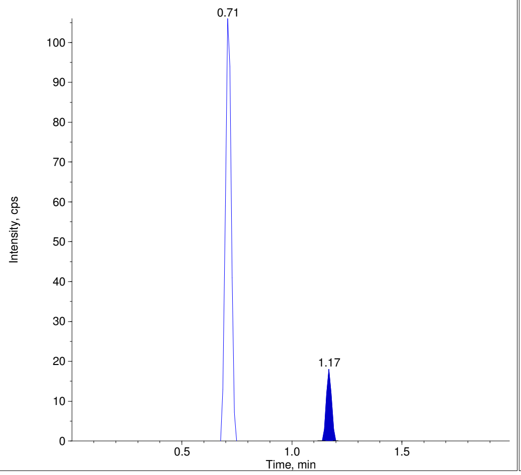

Sample Name: "DOUBLE BLANK" Sample ID: "" File: "2017 03 07_ PQ TEST_FM_AM.wiff"
Peak Name: "PQ_d6(IS)" Mass(es): "541.000/294.000 Da"
Comment: "" Annotation: ""

```
Sample Index:       28
Sample Type:      Unknown
Concentration:       1.00    ng/mL
Calculated Conc:     N/A
Acq. Date:        3/7/2017
Acq. Time:        3:02:39 PM

Modified:            Yes
```

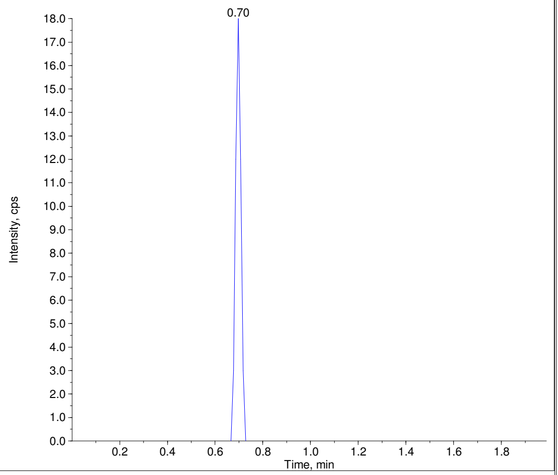

Supplement: S4 File — (PDF) [file pone.0233893.s008.pdf]
